# Supplementary material for: Comparative genomics-based development of a LAMP assay for rapid and reliable in-field detection of Fusarium oxysporum f. sp. cubense tropical race 4
Source: PLoS One. 2026 Apr 29;21(4):e0347645. doi: 10.1371/journal.pone.0347645 (PMC13127957; doi:10.1371/journal.pone.0347645)
Supplement: S1 Methods — (DOCX) [file pone.0347645.s001.docx]

**Comparative genomics-based development of a LAMP assay for rapid and reliable in-field detection of *Fusarium oxysporum* f. sp. *cubense* tropical race 4**

Mikel Arrieta Salgado, Diane Mostert, Sebastien Ravel, Samuel Rozsasi, Veronique Maillot-Lebon, A. Sutanto, C. Hernanto, Mouzdalifa Mmadi, Abdou Azali Hamza, Nadia Adjanoh- Lubin, Béatrice Fenelon, Henri Adreit, Sandrine Fabre, Beatrix Coetzee, Altus Viljoen, Jean Carlier, Yolande Chilin-Charles, Camilo Gianinazzi, Emmanuel Wicker and Isabelle Robène

**Supplementary Methods S1.** DNA extraction protocol on fungal cultures in CIRAD-PHIM

Fungal cultures were grown for five days at 25°C in agitated liquid Nitrate medium. Fungal mats were then vacuum-filtered, rinsed with sterile water, then transferred in 50 mL tubes, flash-freezed in liquid nitrogen, and stored at -80°C until use. For DNA extraction, fungal mycelium was first finely ground in liquid nitrogen, and the powder was transferred in a 5 mL tube (~1 mL powder). Lysis was first done by 2 mL of MATAB buffer (0.1M Tris-HCl pH8, 1.4M NaCl, 0.02M EDTA, 20g.L^-1^ Mixed AlkylTrimethyl Amonium Bromide (MATAB), 10 g. L^-1^ PEG6000, 5 g. L^-1^ Na_2_SO_3_), complemented with 50μL Proteinase K (10 mg.mL^-1^). The mix was first incubated 1h at 65°C with regular twisting. Two mL Chloroform Isoamyl alcohol (CIAA) was then added to the lysate. Tubes were inverted slowly and regularly to complete the emulsion, then centrifuged for 20 min at max speed at room temperature. The supernatant was transferred in 2 mL tubes (1 mL/tube). Each tube then received 20μL RNAse A (10 mg.ml^-1^) and was incubated at 37°C for 45 min. DNA was precipitated by 900μL cold isopropanol per tube. Tubes were then softly inverted and let stand 10 min minimum in a -20°C freezer. Tubes were then centrifuged 15 min at maximum speed (~18000 rpm), and the supernatant was discarded. Pellets were washed wih 1 mL Ethanol 70%, then centrifuged 5 min. Liquid was discarded by draining. Pellets were then dried 10 min in a speed-vacuum dryer, then resuspended in 110μL EB buffer (QIAGEN) for one night at 4°C. The integrity of DNA samples was assessed with gel electrophoresis and the quality and quantity were analysed on both a Nanodrop spectrophotometer (Thermo Fischer Scientific, Waltham, Massachusetts, United States) and with Qubit 4 fluorometer (Thermo Fischer Scientific, Waltham, Massachusetts, United States).
