# Supplementary material for: Comparative genomics-based development of a LAMP assay for rapid and reliable in-field detection of Fusarium oxysporum f. sp. cubense tropical race 4
Source: PLoS One. 2026 Apr 29;21(4):e0347645. doi: 10.1371/journal.pone.0347645 (PMC13127957; doi:10.1371/journal.pone.0347645)
Supplement: Attachment — Submitted filename: PONE-D-25-47570_R1.pdf [file pone.0347645.s014.pdf]

PLOS One

Comparative genomics-based development of a LAMP assay for rapid and reliable in-field detection of Fusarium oxysporum f. sp. cubense tropical race 4

--Manuscript Draft--

Manuscript Number:	PONE-D-25-47570R1
Article Type:	Research Article
Full Title:	Comparative genomics-based development of a LAMP assay for rapid and reliable in-field detection of Fusarium oxysporum f. sp. cubense tropical race 4
Short Title:	A genome-driven Foc-TR4-specific Assay for Field Diagnostic
Corresponding Author:	Emmanuel Wicker<br>CIRAD BIOS: CIRAD Departement Systemes biologiques<br>Montpellier, FRANCE
Keywords:	Tropical race 4; diagnosis; LAMP; fungal plant pathogens; Fusarium oxysporum f. sp. cubense; Fusarium wilt of banana
Abstract:	Fusarium wilt, caused by the soilborne fungus Fusarium oxysporum f. sp. cubense (Foc) Tropical race 4 (TR4) is currently devastating Cavendish banana production worldwide. The availability of accurate and rapid field-deployable detection tools is vital for effective disease prevention and containment practices, as no effective treatment is currently available. Design of reliable molecular detection tools, however, is challenged by genetic similarity between non-pathogenic and target Foc strains that are present in symptomatic plant material. The high genetic diversity present among Foc isolates further complicates the identification of unique genomic regions suitable for diagnostic Current molecular assays are also still mostly confined to laboratory settings and relies on expensive equipment that needs skilled operators to perform. In the present study, a simple loop-mediated isothermal amplification (LAMP) detection assay was developed for direct in-field detection. A comprehensive database of Foc genomes including 148 TR4 genomes and 146 non-target genomes was used, considering the wide diversity within Foc. These genomic sequences were exploited in a comparative genomics in silico pipeline using the KEC method (K-mer elimination by cross-reference), combined with filtering steps using BLASTn, and Illumina read alignments. The specificity of the LAMP primers was validated using a diverse collection of 161 isolates, which included Foc-TR4 isolates, Foc VCGs, F. oxysporum spp. endophytes, other Fusarium spp., and genera which are commonly isolated from banana vascular tissue. The limit of detection reached 102 copies. µL-1 of plasmid DNA, and the target pathogen was successfully identified both from artificially inoculated and naturally infested material in contrasted environments, indicating the LAMP assay to be a robust and reliable tool fully suitable for field diagnosis.
Order of Authors:	Mikel Arrieta Salgado, PhD
	Diane Mostert, PhD
	Sebastien Ravel, Master
	Samuel Rozsasi, Master
	Veronique Maillot-Lebon
	Agus Sutanto, PhD
	Catur Hermanto, PhD
	Mouzdalifa Mmadi, Master
	Abdou Azali Hamza, PhD
	Nadia Adjanoh-Lubin
	Babbitha Fenelon
	Henri Adreit
	Sandrine Fabre, Master

Powered by Editorial Manager® and ProduXion Manager® from Aries Systems Corporation

	Beatrix Coetzee, PhD
	J. Jansen van Vuuren
	Sheryl Le Roux
	Altus Viljoen, Professor, PhD
	Yolande Chilin-Charles
	Jean Carlier, PhD, HDR
	Camilo Gianinazzi
	Emmanuel Wicker, PhD, HDR
	Isabelle Robène, PhD, HDR
Response to Reviewers:	Dear Academic Editor and reviewers,<br><br>The comments and points raised were all considered. We wrote a point-to-point detailed response letter to both the Journal Requirements and the Reviewers comments, available in the file "Response to Reviewers" enclosed.<br>In the name of all co-authors, I would like to thank you for your careful reviews which greatly contributed to improve this manuscript.<br>Yours sincerely,<br>E. Wicker
Additional Information:	
**Question**	**Response**
**Financial Disclosure**<br><br>Enter a financial disclosure statement that describes the sources of funding for the work included in this submission. Review the submission guidelines for detailed requirements. View published research articles from *PLOS ONE* for specific examples.<br><br>This statement is required for submission and **will appear in the published article** if the submission is accepted. Please make sure it is accurate.<br><br>**Funded studies**<br>Enter a statement with the following details:<br>• Initials of the authors who received each award<br>• Grant numbers awarded to each author<br>• The full name of each funder<br>• URL of each funder website<br>• Did the sponsors or funders play any role in the study design, data collection and analysis, decision to publish, or preparation of the manuscript?	Yes

Did you receive funding for this work?	
Please add funding details.<br>as follow-up to "**Financial Disclosure**<br><br>Enter a financial disclosure statement that describes the sources of funding for the work included in this submission. Review the submission guidelines for detailed requirements. View published research articles from *PLOS ONE* for specific examples.<br><br>This statement is required for submission and **will appear in the published article** if the submission is accepted. Please make sure it is accurate.<br><br>**Funded studies**<br>Enter a statement with the following details:<br>• Initials of the authors who received each award<br>• Grant numbers awarded to each author<br>• The full name of each funder<br>• URL of each funder website<br>• Did the sponsors or funders play any role in the study design, data collection and analysis, decision to publish, or preparation of the manuscript?<br><br>Did you receive funding for this work?"	This study was funded by the Projet France-Relance (PFR) CIRAD-QUALIPLANTE (2022-2024), and by the European Union Horizon 2020 Research and Innovation Program Marie Sklodowska-Curie Fellowship, through the INDICANTS project grant nr 890856.
Please select the country of your main research funder (please select carefully as in some cases this is used in fee calculation).<br>as follow-up to "**Financial Disclosure**<br><br>Enter a financial disclosure statement that describes the sources of funding for the work included in this submission. Review the submission guidelines for detailed requirements. View published research articles from *PLOS ONE* for specific examples.<br><br>This statement is required for submission and **will appear in the published article** if the submission is accepted. Please make	FRANCE - FR

sure it is accurate.<br><br>**Funded studies**<br>Enter a statement with the following details:<br>• Initials of the authors who received each award<br>• Grant numbers awarded to each author<br>• The full name of each funder<br>• URL of each funder website<br>• Did the sponsors or funders play any role in the study design, data collection and analysis, decision to publish, or preparation of the manuscript?<br><br>Did you receive funding for this work?"	
**Competing Interests**<br><br>Use the instructions below to enter a competing interest statement for this submission. On behalf of all authors, disclose any competing interests that could be perceived to bias this work—acknowledging all financial support and any other relevant financial or non-financial competing interests.<br><br>This statement is required for submission and **will appear in the published article** if the submission is accepted. Please make sure it is accurate and that any funding sources listed in your Funding Information later in the submission form are also declared in your Financial Disclosure statement.<br><br>View published research articles from *PLOS ONE* for specific examples.	The authors have declared that non competing interests exist.

**NO authors have competing interests**<br><br>Enter: *The authors have declared that no competing interests exist.*<br><br>**Authors with competing interests**<br><br>Enter competing interest details beginning with this statement:<br><br>*I have read the journal's policy and the authors of this manuscript have the following competing interests: [insert competing interests here]*<br><br>* typeset	
**Ethics Statement**<br><br>Enter an ethics statement for this submission. This statement is required if the study involved:<br><br>• Human participants<br>• Human specimens or tissue<br>• Vertebrate animals or cephalopods<br>• Vertebrate embryos or tissues<br>• Field research<br><br>Write "N/A" if the submission does not require an ethics statement.<br><br>General guidance is provided below. Consult the submission guidelines for detailed instructions. **Make sure that all information entered here is included in the Methods section of the manuscript.**	Field samplings were done in Java, Indonesia, as granted by the National Research and Innovation Agency BRIN.

**Format for specific study types**<br><br>**Human Subject Research (involving human participants and/or tissue)**<br>• Give the name of the institutional review board or ethics committee that approved the study<br>• Include the approval number and/or a statement indicating approval of this research<br>• Indicate the form of consent obtained (written/oral) or the reason that consent was not obtained (e.g. the data were analyzed anonymously)<br><br>**Animal Research (involving vertebrate animals, embryos or tissues)**<br>• Provide the name of the Institutional Animal Care and Use Committee (IACUC) or other relevant ethics board that reviewed the study protocol, and indicate whether they approved this research or granted a formal waiver of ethical approval<br>• Include an approval number if one was obtained<br>• If the study involved *non-human primates*, add *additional details* about animal welfare and steps taken to ameliorate suffering<br>• If anesthesia, euthanasia, or any kind of animal sacrifice is part of the study, include briefly which substances and/or methods were applied<br><br>**Field Research**<br><br>Include the following details if this study involves the collection of plant, animal, or other materials from a natural setting:<br>• Field permit number<br>• Name of the institution or relevant body that granted permission	
**Data Availability**<br><br>Authors are required to make all data underlying the findings described fully available, without restriction, and from the time of publication. PLOS allows rare exceptions to address legal and ethical concerns. See the PLOS Data Policy and FAQ for detailed information.	Yes - all data are fully available without restriction

A Data Availability Statement describing where the data can be found is required at submission. Your answers to this question constitute the Data Availability Statement and **will be published in the article**, if accepted.<br><br>**Important:** Stating 'data available on request from the author' is not sufficient. If your data are only available upon request, select 'No' for the first question and explain your exceptional situation in the text box.<br><br>Do the authors confirm that all data underlying the findings described in their manuscript are fully available without restriction?	
**Describe where the data may be found in full sentences. If you are copying our sample text, replace any instances of XXX with the appropriate details.**<br><br>• If the data are **held or will be held in a public repository**, include URLs, accession numbers or DOIs. If this information will only be available after acceptance, indicate this by ticking the box below. For example: *All XXX files are available from the XXX database (accession number(s) XXX, XXX.*).<br>• If the data are all contained **within the manuscript and/or Supporting Information files**, enter the following: *All relevant data are within the manuscript and its Supporting Information files.*<br>• If neither of these applies but you are able to provide **details of access elsewhere**, with or without limitations, please do so. For example:<br><br>*Data cannot be shared publicly because of [XXX]. Data are available from the XXX Institutional Data Access / Ethics Committee (contact via XXX) for researchers who meet the criteria for access to confidential data.*<br><br>*The data underlying the results presented in the study are available from (include the name of the third party*	All relevant data are within the manuscript and its Supporting Information files.

and contact information or URL).<br>• This text is appropriate if the data are owned by a third party and authors do not have permission to share the data.<br><br>* typeset	
Additional data availability information:	

Powered by Editorial Manager® and ProduXion Manager® from Aries Systems Corporation

Manuscript
Click here to access/download;Manuscript;Draft_TR4_LAMP_PlosOne_REV

Comparative genomics-based development of a LAMP assay for rapid and reliable in-field detection of *Fusarium oxysporum* f. sp. *cubense* tropical race 4

M. Arrieta*[1,2], D. Mostert[4], S. Ravel[1,2], S. Rozsasi[3,4], V. Maillot-Lebon[3], A. Sutanto[5], C. Hermanto[5], Mouzdalifa Mmadi[6], Abdou Azali Hamza[6], N.A. Lubin[1,2], B. Fenelon[3], H. Adreit[1,2], S. Fabre[1,2], B. Coetzee[4], J. Jansen van Vuuren[4], S. Le Roux[4], A. Viljoen[4], Y. Chilin-Charles[2,7], J. Carlier[1,2], C. Gianinazzi[8], E. Wicker*[1,2] and I. Robène*[3,4]

[1] CIRAD, UMR PHIM, Montpellier, France.

[2] PHIM, Plant Health Institute, Univ Montpellier, INRAe, CIRAD, Institut Agro, Montpellier, France

[3] CIRAD, UMR PVBMT, Saint-Pierre, Réunion Island, France.

[4] Department of Plant Pathology, Stellenbosch University, Stellenbosch, South Africa.

[5] BRIN, Cibinong, Kabupaten Bogor, Indonesia

[6] Institut National de Recherche pour l'Agriculture, la Pêche et l'Environnement (INRAPE), Moroni, Union des Comores

[7] CIRAD, UMR PHIM, Capesterre-Belle-Eau, Guadeloupe, France.

[8] QUALIPLANTE SAS, Laverune, France.

***Corresponding authors**

E-mail : mikarriet@gmail.com, emmanuel.wicker@cirad.fr, isabelle.robene@cirad.fr

ORCID ID:

- Mikel Arrieta: https://orcid.org/0000-0001-7209-0672
- Diane Mostert: https://orcid.org/ 0000-0002-3382-8555
- Sebastien Ravel: https://orcid.org/ 0000-0001-6663-782X
- Samuel Rozsasi: https://orcid.org/ 0009-0008-7182-3764

- Beatrix Coetzee: https://orcid.org/ 0000-0001-5374-8918
- Altus Viljoen: https://orcid.org/ 0000-0001-7146-0491
- Yolande Chilin-Charles: https://orcid.org/ 0009-0009-1584-5149
- Jean Carlier: https://orcid.org/0000-0002-6967-1852
- Emmanuel Wicker: https://orcid.org/0000-0003-0927-7404
- Isabelle Robene: https://orcid.org/0000-0003-2012-4652

Short title: A genome-driven Foc-TR4-specific Assay for Field Diagnostic

Abstract

Fusarium wilt, caused by the soilborne fungus *Fusarium oxysporum* f. sp. *cubense* (Foc) Tropical race 4 (TR4) is currently devastating Cavendish banana production worldwide. The availability of accurate and rapid field-deployable detection tools is vital for effective disease prevention and containment practices, as no effective treatment is currently available. Design of reliable molecular detection tools, however, is challenged by genetic similarity between non-pathogenic and target Foc strains that are present in symptomatic plant material. The high genetic diversity present among Foc isolates further complicates the identification of unique genomic regions suitable for diagnosis. Current molecular assays are also still mostly confined to laboratory settings and relies on expensive equipment that needs skilled operators to perform. In the present study, a simple loop-mediated isothermal amplification (LAMP) detection assay was developed for direct in-field detection. A comprehensive database of Foc genomes including 148 TR4 genomes and 146 non-target genomes was used, considering the wide diversity within Foc. These genomic sequences were exploited in a comparative genomics *in silico* pipeline using the KEC method (K-mer elimination by cross-reference), combined with filtering steps using BLASTn, and Illumina read alignments. The specificity of the LAMP primers was validated using a diverse collection of 161 isolates, which included Foc-TR4 isolates, Foc VCGs, *F. oxysporum* spp. endophytes, other *Fusarium* spp., and genera which are commonly isolated from banana vascular tissue. The limit of detection reached 10^2 copies μL^{-1} of plasmid DNA, and the target pathogen was successfully identified both from artificially inoculated and naturally infested material in contrasted environments, indicating the LAMP assay to be a robust and reliable tool fully suitable for field diagnosis.

Keywords: Tropical race 4, diagnosis, LAMP, fungal plant pathogens, *Fusarium oxysporum* f. sp. *cubense*, Fusarium wilt of banana

Introduction

Fusarium wilt of banana is a soilborne vascular disease that threatens global banana production, and is considered one of the most destructive plant diseases worldwide [1-3]. The causal agent is *Fusarium oxysporum* f. sp. *cubense* (Foc), a highly diverse fungal species which different strains have caused two major epidemics in the last century. Foc-Race 1 caused destruction of the most widely cultivated variety during the 20th century, Gros Michel [4, 5]. This first Fusarium wilt epidemic could only be brought under control after the 1950s by the deployment of a resistant cultivar called Cavendish, which currently represents the 99% of worldwide banana exports and more than 40% of global banana production [6-9]. The "return of the banana Fusarium wilt calamity" occurred in the early 1990s with outbreaks in Indonesia, Malaysia, and Taiwan, of a new Foc race called "tropical race 4" (TR4) [2, 10]. This race affects Cavendish banana cultivars, as well as the other cultivars susceptible to Race 1 [2, 11, 12]. The resilience of Foc due to the production of survival structures called chlamydospores, and the acceleration of global trade during the last decades favoured the spread of the pathogen. Within the last two decades it has rapidly spread from its origin in Asia to major banana growing regions in the Middle East, Africa and Latin America [2, 3, 13-15].

Millions of people worldwide rely on banana both as a staple food and as a source of income [16], and hence solutions for the Foc-TR4 epidemic are crucial to avoid consequences of another wipeout of the currently preferred cultivar. As a soilborne pathogen, Foc can be transmitted through irrigation, watercourses and floodwater, as well as field tools, vehicles, machinery, contaminated plants and soil. It can also survive in the soil for decades [4, 11, 17]. Once Foc is established within a field, there is no effective control methods to ensure its complete eradication [11].

Early detection and containment are crucial in response to Foc-TR4 epidemics in the absence of disease management strategies [11]. Molecular detection tools are the preferred method for disease

detection and there is an abundance of assays published including PCR [18, 19], quantitative qPCR [20, 21], and loop-mediated isothermal amplification (LAMP), as reviewed in Roberts *et al.* [22]. The design of a reliable molecular detection assay for Foc-TR4 has faced several challenges. First molecular assays targeted ~~asingle~~ sequences or single nucleotide polymorphism (SNPs) in core genes (e.g. the intergenic spacer region (IGS)), whereas these genes are conserved among both target and non-target Fusaria [23]. Consequently, endophytic *F. oxysporum* with homology in these regions, are often identified as false positives in molecular assays designed in these core gene regions [24]. Another challenge consists of the specific identification of the Foc-TR4 lineage among the different races, VCGs, and Foc genetic clades. The banana Fusarium Wilt agent, Foc, is indeed a polyphyletic and diverse organism, composed of three genetic clades (A, B, C *sensu* O'Donnell et al., 2004 [25]), corresponding respectively to the *Fusarium oxysporum* Species Complex (FOSC) Clades 1, 2, and 3 [26], eight subclusters (Foc-SC), more than 24 vegetative-compatibility groups (VCGs) [27] and four races based on the host specificity.

Foc TR4 isolates can cause disease to Cavendish banana in tropical conditions [28]. According to various authors, it is a synonym of the VCG 01213/16 [29, 30][, which has been controversially reclassified as *Fusarium odoratissimum* [31], and which can also include closely related VCG 0121 [2]. Race 4 is further divided into Subtropical Race 4 (STR4), which infects Cavendish only in subtropical conditions after stress predisposition [32]. Furthermore, Race 4 isolates can also affect varieties affected by Race 1 and 2, which makes the race concept confounding and challenges development of accurate molecular diagnostics [22].

Current Foc-TR4 molecular assays are also mostly confined to laboratory settings and rely on expensive molecular equipment and requires skilled operators to perform these assays. There is a need for a TR4 diagnostic system to be Point-of-care (POC), implying that the testing and assay be carried directly in the field and with rapid and simplified protocols. Loop-mediated isothermal

amplification (LAMP) assays enable a rapid, simple, sensitive and robust detection [33, 34]. LAMP machines are usually portable, and the enzyme used is often less sensitive to background inhibitors, which makes it more compatible with simplified DNA extraction methods that can be done directly from sampled plant material. Among the different LAMP assays developed for detecting FocTR4, some are unable to distinguish between TR4 and STR4 [19, 35] or generate false positives [36]. Conversely, the LAMP assay developed by Ordóñez et al.[37] appears to be a reliable tool for detecting FocTR4.

In this study, a novel Foc-TR4 LAMP assay was designed with the aim to be field deployable. We considered a large and comprehensive genomic and genetic collection of fungal strains to identify specific markers, design adequate LAMP primers, as well as to address the limitations and specificity of the existing LAMP systems. The LAMP assay was designed with a simple and cost-effective DNA extraction protocol which can be conducted by unskilled operators. We compared its diagnostic performance with existing molecular tools including the Ordóñez LAMP assay [37]. The assay successfully detected Foc-TR4 both from artificially inoculated and naturally infected banana material and could be conducted directly in field settings. The proposed system provides a useful tool useful for early Foc-TR4 detection during surveillance and containment.

Materials and methods

Genomic resources

The genomic resources used in this study included a total of 294 genomes (summarized in Table 1 and detailed in S1 Table)**.** Among these genomes are those available in an in-house genomic database shared between CIRAD and Stellenbosch University including 118 target Foc-TR4 genomes, 47 genomes from other Foc VCGs and 2 endophytic *F. oxysporum* isolates. Additionally, 127 genomes

were downloaded from the NCBI database (National Center for Biotechnology Information, Maryland, United States) or the NGDC (National Genomics Data Centre, Beijing, China). These included 31 target Foc-TR4 genomes, 16 non-target Foc genomes, 51 genomes of various other *formae speciales* of *Fusarium oxysporum*, 29 other genomes from *Fusarium* genus, and other microbial genera commonly associated with bananas. These genomic resources were available in three different formats: Illumina short read sequencing data (n=172), genome assemblies that had been sequenced using the Oxford Nanopore Technology (ONT) (n=21), and genome assemblies that were publicly available (n=101).

Table 1: Summary of the genomic resources and DNA from microbial isolates used in this study.

Species	Clade / Foc-SC[a]	VCG	Race	Genomic Resources				TOTAL DNA Samples
				**Illumina Reads**	**ONT Assembly**	**Public Assembly**	**TOTAL Genomes**	
F. oxysporum f. sp. cubense	A/1	0120	STR4	4	1	1	**6**	**6**
F. oxysporum f. sp. cubense	A/1	0120/15	STR4	5	0	0	**5**	**4**
F. oxysporum f. sp. cubense	A/1	01210	STR4	7	0	0	**7**	**1**
F. oxysporum f. sp. cubense	A/1	01219	STR4	1	0	0	**1**	**1**
F. oxysporum f. sp. cubense	A/1	0122	STR4	1	1	0	**2**	**2**
F. oxysporum f. sp. cubense	A/1	0129/11	STR4	1	0	0	**1**	**2**
F. oxysporum f. sp. cubense	A/1	01215	STR4	1	0	0	**1**	**1**
F. oxysporum f. sp. cubense	A/1	01227		0	0	0	**0**	**1**
F. oxysporum f. sp. cubense	A/1	0126	STR4	1	0	0	**1**	**1**
F. oxysporum f. sp. cubense	A/1	0129	STR4	1	0	0	**1**	**1**
F. oxysporum f. sp. cubense	A/1	SMV 1	UNK	0	0	0	**0**	**1**
F. oxysporum f. sp. cubense	A/1	SMV 2	UNK	0	0	0	**0**	**1**
F. oxysporum f. sp. cubense	A/2	0121	Race 4	1	5	0	**6**	**9**
F. oxysporum f. sp. cubense	A/2	01213/16	**TR4**	126	14	8	**148**	**46**
F. oxysporum f. sp. cubense	A/2	SMV 3	UNK	0	0	0	**0**	**1**
F. oxysporum f. sp. cubense	B/4	01212	Race 1	1	0	0	**1**	**1**
F. oxysporum f. sp. cubense	B/4	01220	Race 1	2	0	1	**3**	**1**
F. oxysporum f. sp. cubense	B/4	01222	Race 1	1	0	0	**1**	**1**
F. oxysporum f. sp. cubense	B/4	0124	Race 1	3	0	1	**4**	**3**
F. oxysporum f. sp. cubense	B/4	0124/25	Race 1	0	0	0	**0**	**1**
F. oxysporum f. sp. cubense	B/4	0124/5	Race 1	1	0	0	**1**	**2**
F. oxysporum f. sp. cubense	B/4	0124/5/8	Race 1	1	0	0	**1**	**1**
F. oxysporum f. sp. cubense	B/4	0125	Race 1	1	0	1	**2**	**1**
F. oxysporum f. sp. cubense	B/4	0128	Race 1	1	0	0	**1**	**3**
F. oxysporum f. sp. cubense	B/4	UNK	Race 1	0	0	0	**0**	**3**
F. oxysporum f. sp. cubense	B/4	01228	UNK	0	0	0	**0**	**1**
F. oxysporum f. sp. cubense	B/4	01229	UNK	0	0	0	**0**	**1**
F. oxysporum f. sp. cubense	B/4	01232	UNK	0	0	0	**0**	**1**
F. oxysporum f. sp. cubense	B/4	01233	UNK	0	0	0	**0**	**1**
F. oxysporum f. sp. cubense	B/4	01236	UNK	0	0	0	**0**	**1**
F. oxysporum f. sp. cubense	B/4	0124/22	UNK	1	0	0	**1**	**0**
F. oxysporum f. sp. cubense	B/4	0128/20	UNK	0	0	0	**0**	**1**
F. oxysporum f. sp. cubense	B/4	SMV 4	UNK	0	0	0	**0**	**1**
F. oxysporum f. sp. cubense	B/4	SMV 5	UNK	0	0	0	**0**	**1**
F. oxysporum f. sp. cubense	B/4	SMV 6	UNK	0	0	0	**0**	**1**
F. oxysporum f. sp. cubense	B/5	01214	UNK	1	0	0	**1**	**5**
F. oxysporum f. sp. cubense	B/5	01231	UNK	0	0	0	**0**	**1**

F. oxysporum f. sp. *cubense*	B/6	01217	Race 1/2	1	0	0	**1**	**0**
F. oxysporum f. sp. *cubense*	B/6	01223	UNK	1	0	0	**1**	**0**
F. oxysporum f. sp. *cubense*	B/6	01224	UNK	1	0	0	**1**	**1**
F. oxysporum f. sp. *cubense*	B/6	0123	Race 1/2	1	0	0	**1**	**2**
F. oxysporum f. sp. *cubense*	B/7	01218	UNK	1	0	0	**1**	**1**
F. oxysporum f. sp. *cubense*	B/7	01234	UNK	0	0	0	**0**	**1**
F. oxysporum f. sp. *cubense*	B/7	01235	UNK	0	0	0	**0**	**1**
F. oxysporum f. sp. *cubense*	B/7	SMV 10	UNK	0	0	0	**0**	**1**
F. oxysporum f. sp. *cubense*	B/8	01221	Race 1/2	1	0	0	**1**	**1**
F. oxysporum f. sp. *cubense*	B/8	01230	UNK	0	0	0	**0**	**1**
F. oxysporum f. sp. *cubense*	C/3	01226	UNK	0	0	0	**0**	**3**
F. oxysporum f. sp. *cubense*	UNK	UNK	Race 1	1	0	3	**4**	**5**
F. oxysporum f. sp. *cubense*	UNK	UNK	STR4	12	0	0	**12**	**0**
F. oxysporum f. sp. *cubense*	UNK	UNK	UNK	0	0	5	**5**	**0**
F. oxysporum			Banana endophyte	2	0	0	**2**	**3**
F.oxysporum, other *formae speciales* [b]				0	**29**	**30**	**12**	0
F. oxysporum, others [c]				0	0	22	**22**	**12**
Fusarium, other species [d]				0	29	29	3	0
Nectria sp. FW16.1				0	0	1	**1**	**0**
Pseudocercospora fijiensis				0	0	0	**0**	**3**
Ralstonia solanacearum				0	0	0	**0**	**1**
**Total Target (TR4)**				**126**	**14**	**8**	**148**	**46**
**Total Non Target**				**46**	**7**	**93**	**146**	**115**
**Total**				**172**	**21**	**101**	**294**	**161**

The data type used is indicated as Illumina short read genomes, ONT-based assemblies, or Public assemblies (from publicly available genomes). All F. *oxysporum* f. sp. other than *cubense* were not pathogenic on banana.

[a]The Clade and VCG classification is based on Mostert *et al.*, 2022 [27]. SMV: Single-Member VCG. UNK: Unknown.

[b] The other *formae speciales* of *Fusarium oxysporum* tested were: *canariensis, cepae, ciceris, conglutinans, cucumerinum, cyclamini, dianthi, fragariae, koae, lini, lycopersici, radicis-lycopersici, radicis-vanillae, rapae, strigae, vanillae, vasinfectum, spinaciae*

[c] *F.oxysporum* strains which were not assigned to a specific *forma specialis*.

[d] The other *Fusarium* species tested included *F.agapanthi, F. avenaceum, F. falciforme, F. fujikuroi, F. graminearum, F. haematococcum, F. langsethiae, F. louisianense, F. metavorans, F. neocosmosporiellum, F. pseudoanthophilum, F. pseudonygamai, F. ramigenum, F. redolens, F. solani, F. udum, F. vanettenii, F. venenatum, F. verticillioides*

Identification of a target genomic region

The K-mer elimination by cross-reference (KEC) method v.1.0 [38] (Step 1 in Fig 1) was used to identify target specific candidate sequences. This approach is fed by genome assemblies that are user-defined as target or non-target genomes. In addition to the 21 ONT-based in-house assemblies [39, 40], and the 101 publicly available genomes, all the Illumina short read genomes (172) were assembled to maximise the number of input genomes. The assembler ABySS **v.2** [41] software was used with an automated optimised best K-mer selection through the *ABYSS_launch.py* workflow, written by Florian Charriat (https://github.com/FlorianCHA/script_paper). Importantly, these assemblies were repeat masked using RepeatMasker (http://repeatmasker.org/).

Fig 1. Pipeline followed to find TR4-specific sequences. Step 1 assemblies of target and not-target genomes were used for the KEC (K-mer elimination by cross-reference) method described by Beran et al.)[38] to obtain an initial list of target specific candidate sequences. These sequences were queried (**Step 2**) in an internal BLAST to double-check the presence/absence on non-target genomes. The remaining candidate sequences were filtered in two ways. Firstly, based on the results obtained when a query was done with BLAST on the NCBI online database (**Step 3**), whilst the second (**Step 4**) aligned Illumina reads on the candidate sequences with RattleSNP software (https://github.com/sravel/RattleSNP), and computed coverage of query reads on sequence tested. Figure created with Miro™.

The ONT-based inhouse assembly of CAV807 ([39] available upon request) was chosen as the main target reference genome (master) on the KEC method, using the "pool" feature with the rest of target genomes. A combination of parameters was tested to do KEC computations (test details included on S2 Table) with a minimum output sequence size range from 60 to 500 bp, K-mer size ranges from 13 to 19, different input populations, and including or excluding reverse complement sequences, all of which produced different output candidate sequence lists. All these sequences were then merged on a single file which was used as reference sequences on the following steps (Steps 24 in Fig 1).

To verify that the output sequences were present on the target genomes while absent from non-target genomes, an internal BLASTN query (Step 2, Fig 1) was done for all the candidate sequences on all the available genomes; candidates that had high BLAST hits on non-targeted genomes (more than 90% identity of full queried sequence) were discarded. The best sequence candidates were also queried against fungal genomes (Whole-Genome shotgun contigs database version for Fungi, taxid 4751) on the NCBI database (step 3, Fig 1) (https://blast.ncbi.nlm.nih.gov/Blast.cgi). The candidate sequences were regarded as good targets for primer design if (i) they got a hit on all the available TR4 genomes on NCBI (named "*Fusarium oxysporum* f. sp. *cubense* TR4", or "*F.odoratissimum*"), and (ii) they got the minimum hit on non-TR4 genomes. As a reference, internal BLASTN and NCBI database queries were performed in parallel using the sequence targeted in the Ordóñez LAMP assay [37], which was the latest published LAMP TR4-diagnostic region at the time of this study.

Additionally, the genomic Illumina reads of all genomes available in this format were mapped (Step 4 Fig 1 on the candidate sequences used as references, using the workflow RattleSNP (https://github.com/sravel/RattleSNP). Mapping 150 bp reads on such a short reference sequence may result in misalignments. To avoid this, the candidate sequences were extended 1000 bp up and downstream using the unmasked version of the ONT-based CAV807 assembly, before the alignment step, The sequences which had the highest percentages of coverage among all target Illumina genomes (TR4), and lowest coverage on non-targeted genomes, were selected.

PCR primer design and initial screening of selected candidate sequences

Initial screening of best performing candidate sequences was done either in PCR or Real-time quantitative PCR (qPCR). Primers were designed using Primer 3 (https://primer3.ut.ee/), and assays were performed on a bCUBE machine (Hyris Ltd, London, United Kingdom), adjusting the amplification protocol for each case primer system. For PCRs, the Promega GoTaq® G2 MasterMix (Promega, Wisconsin, United States) kit was used, and PCR products were assessed on a 1.5%

Agarose Gel, whilst for qPCR the SYBR™ Select Master Mix qPCR (Thermo Fischer, Waltham, Massachusetts, United States) was used.

LAMP primer design and assay conditions

LAMP primers were designed using the Primer explorer V5 software (primerexplorer.jp/lampv5e/index.html). When software did not yield Loop primers by default settings, design was done manually. A similar approach was used for the design of Swarm primers for which design option is not included in the software. LAMP reactions were performed on the portable Genie II machine (Optigene, Blatchford, United Kingdom), in a total reaction volume of 25 µL, containing 15 µL ISO-DR004 Isothermal Mastermix (OptiGene, Horsham, UK), 2.5 µL of pre-mixed Primer Mix, (resulting in final concentrations of 0.2 µM of each F3 and B3 primers, 0.8 µM of each FIP and BIP primers, 0.4 µM of each loop primer), and 5µL of template DNA. Note that within the specificity tests, 1 µL of template DNA was used instead of 5µL. LAMP reactions were run at 65°C for 30 min, followed by a 6 min annealing step with temperatures varying from 98°C to 80°C at a speed of 0.05°C per second. Amplification curves associated with a "Time to Result value" (TTR values) and annealing temperature (*Ta*) peaks were generated and recorded during the LAMP reaction. A sample was considered positive if (i) the average TTR of the replicates of a sample was below the established minimum TTR threshold (30:00), (ii) if the average *Ta* peak was ± 1 °C from the reference Ta (relative to each LAMP system), and (iii) if the fluorescence of the Ta peak reached a minimum threshold value of 1500. By convention, a value of 31:00 (just above the time limit) was assigned to a negative result (TTR=00:00) for the calculations and graphical representations (as in [42]). In the case of sensitivity tests, the limit of detection (LOD) was considered as the minimum dilution of DNA at which the mean of the replicates could produce a positive result at a 95% of confidence level.

Reference assays

The Ordóñez LAMP assay [37] was used with the LAMP conditions described above and the program described in the publication. Additionally Two TR4-specific qPCRs systems were considered as references: (i) The hydrolysis probe-based real-time assay developed by Aguayo[20], which is the current reference method in France by the French Agency for Food, Environmental and Occupational Health and Safety, ANSES [43], along with the Promega GoTaq™ Probe qPCR Master Mix (Promega, Wisconsin, United States); and (ii) the SYBR™-based real-time assay [21] along with the SYBR™ Select Master Mix qPCR (Thermo Fischer, Waltham, Massachusetts, United States). A sample was considered positive when the mean Threshold Cycle (Ct) over all replicates was below a threshold of 35 cycles.

Specificity testing

A set of 161 DNA samples was used for specificity testing in the present study; including 46 target Foc-TR4 isolates and 82 non-target Foc isolates representing intraspecies diversity (S3 Table). In addition, other non-target isolates included *F. oxysporum* endophytes and other *F. oxysporum formae speciales* (n=25), other *Fusarium* spp. (n=5), and pathogens of banana (3 isolates of *Pseudocercospora fijiensis*, causing the black Sigatoka; and one isolate of *Ralstonia solanacearum,* causing the Moko disease). Moko strain was grown onto tetrazolium chloride medium (TZC) at 28 °C [44]. Bacterial cultures grown overnight were used for DNA extraction using a DNeasy Blood and tissues kit (Qiagen, Courtaboeuf, France). Fungal cultures were grown on potato dextrose agar (PDA) for seven days at 25°C, then transferred to a liquid medium (Nitrate medium; Yeast Nitrogen Base: 1.7 g L^{-1}, Sucrose : 30 g L^{-1}, KNO_3: 10.11 g L^{-1}; M. de Saint & M. Rep, personal comm. 2020) in an Erlenmeyer flask for five to seven days. The mycelia were filtered through sterile cheesecloth by rinsing with sterile water. Mycelia of each isolate was then ground with mortar and pestle in liquid nitrogen prior to DNA extractions performed following an in-house protocol (S1 Methods). DNA quantity and quality was assessed on a Nanodrop™ spectrophotometer (Thermo Fischer, Waltham, Massachusetts, United States). DNA concentration was adjusted to 1-2 ngμL^{-1} before it was tested

with optimised LAMP assays. The rest of extractions were performed in Stellenbosch University following protocols detailed ealier[45, 46].

Sensitivity testing

Different DNA samples were prepared for sensitivity tests including pure plasmid DNA samples, plasmid spiked plant material, as well as plant material artificially inoculated with conidial suspensions.

Sensitivity based on plasmid dilution series

A plasmid containing the selected DNA sequence targeted by the LAMP primers was ordered from Eurofins (Eurofins, Luxembourg) (S1 Fig). This was initially resuspended in TE buffer (Sigma-Aldrich, Massachusetts, United States) as recommended by the manufacturer, and dilution series were prepared in water (10^8 copies μL^{-1} to 10^0 copies μL^{-1}).

Sensitivity based on plasmid-spiked plant material

Plasmid-spiked plant tissue samples were prepared by first grinding healthy plant tissue from corm and pseudostem in liquid nitrogen, after which approximately 100 mg of healthy tissue collected in Eppendorf tubes. To spike the samples, 10 µL of each plasmid dilution were then mixed with the ground material. DNA was then extracted with the DNeasy Plant Kit protocol as per manufacturer's instructions (Qiagen, Hilden, Germany), but eluted in a final volume of 100 µL, which was used to calculate the final concentration of plasmid in each sample (from 10^5 conidia μL^{-1}, downto 0 conidia μL^{-1}).

Sensitivity based on plant material inoculated with conidial suspensions

The Foc-TR4 isolate MAY0001 was used to prepare an initial conidial suspension. Conidial concentrations (10^7 conidia mL^{-1} downto 10^0 conidia mL^{-1}) were adjusted with haemocytometer (KOVA, California, United States) visualised under a light microscope. Each conidial dilution was mixed with about 100 mg of ground healthy plant tissue from corm and pseudostem and then centrifuged at 14 rpm for 5 mins. DNA extraction was performed on the pellet using the DNeasy Plant Kit (Qiagen, Hilden, Germany). Extractions were done in duplicate for each prepared concentration (from 10^5 conidia µL^{-1}, to 0 conidia µL^{-1}).

Detection from artificially inoculated material

Conidial suspensions of MAY0001 were prepared as described above. Three-month old tissue culture-derived banana plants which were previously hardened in the CIRAD-PHIM glasshouse Montpellier, France. Infections were conducted by pouring 20 ml conidial suspensions at a concentration of $1x10^6$ conidia mL^{-1} around the base of plant. After ten weeks of incubation (30°C day/ 26°C night, 90% RH, 2h of additional lighting (300W m^{-2}) to ensure 12h daylight during winter), infection was assessed by cross-sections of the corm. Where infection was evident including reddish brown discoloration of the vascular tissues and rhizomes, tissue was collected and frozen at -20°C. Tissue was then ground to a powder in liquid nitrogen and transferred to Eppendorf™ tubes (Eppendorf, Hamburg, Germany). About 100 mg of plant material from each sampled plant were used for DNA extraction with the DNeasy Plant Kit following the instructions of the manufacturer (Qiagen, Hilden, Germany). DNA quantity was measured with Nanodrop. The sensitivity of the optimised LAMP assay to detect Foc-TR4 DNA was compared to the mentioned published assays .

In-field deployment and detection from naturally infested plant material

The optimised LAMP assay was deployed on site during a field survey in Java, Indonesia, organized in collaboration with the local institute BRIN (National Research and Innovation Agency). The survey was conducted in the Cianjur regency, at four different plots in the Cikalongkulon and Mande districts where Foc-TR4 were previously reported. Thirty-two plants of different banana cultivars, including both Cavendish and non-Cavendish cultivars of various genomic formulas (detailed in S7 Table), were sampled, making a total of 70 samples collected from fruit, core of pseudostem, outer pseudostem, corm, or peduncles, which were analysed both with the LAMP assay optimised in this study, with published LAMP [37] and the Matthews qPCR [21]. Additionally, eight pseudostem samples collected from Comoros (Grande Comore) and Vietnam respectively that were sent to Stellenbosch University for VCG characterisation, were also analysed.

A rapid DNA extraction method was modified from Robène *et al* [42], for extraction from collected pseudostem tissues: About 250 mg of pseudostem tissue was transferred to Bioreba extraction and.an volume of 1 ml 0.5 M NaOH including 2% PVP added, which was then ground with hand grinder (Bioreba, Reinach, Switzerland). After grinding, 5 µL of the resulting homogenate liquid was then collected and diluted into 195 µL 100 mM Tris (pH 8.0). This homogenate was immediately analysed in LAMP assays. In parallel, the Mathews qPCR [21] was performed on the duplicate samples extracted with a magnetic bead extraction kit according to the supplier's protocol (Sera-Mag Select, cytiva, Marlborough, USA)

Statistical analysis for the test comparisons

The results of the LAMP assays on plant material were compared with each other and with the qPCR results by the Cohen's kappa index and the McNemar's test [47]. Cohen's kappa index was interpreted as in McHugh 2012[48]to evaluate the agreement between techniques: Kappa $\leq$ 0 corresponds to no agreement, 0.01–0.20 is "none to slight", 0.21–0.40 is "fair", 0.41– 0.60 is "moderate", 0.61–0.80 is "substantial", and 0.81–1.00 is "near-perfect". Additionally, different

diagnostic parameters were calculated, such as the Sensitivity, Specificity, Positive Predictive Value (PPV), Negative Predictive Value (NPV), and Accuracy as in [49]. A McNemar's p-value below 0.05 was taken as evidence to reject the null hypothesis of equal marginal proportions, indicating a statistically significant difference in detection outcomes between the two methods compared. This would reflect an imbalance in discordant results, i.e. a real shift in the numbers of false positives versus false negatives, which in turn underlies any observed differences in diagnostic parameters such as sensitivity and specificity.

Ethics statement

Field samplings were done in Java, Indonesia, as granted by the National Research and Innovation Agency BRIN (Badan Riset dan Inovasi Nasional, https://www.brin.go.id).

Results

In silico identification and validation of candidate target region

A total of 1170 candidate target sequences were yielded with the KEC method. The internal BLASTN filtering step (by querying the candidate sequences against the same database) (Step 2, Fig 1) was found a mandatory step, as many candidates were detected both in target and non-target genomes (generally phylogenetically close lineages). Duplicated candidate sequences were also removed, selecting 30 candidate sequences to analyse (details in S2 Table). The list was further reduced to 10 candidate sequences when the amount of non-target genomes was increased in the analysis and sequences showing multiple copies over the target regions were discarded. After the *in silico* specificity testing done by BLAST query on NCBI database on Whole Genome Contigs for all Fungi (taxid 4751) (Step 3, Fig 1), three candidates were finally selected including Candidate 5 (709 bp),

Candidate 23 (417 bp) and Candidate 27 (515 bp) located in contigs VMNF01000005.1, VMNF01000003.1 and VMNF01000004.1 of UK0001 reference genome respectively (contigs 12, 3, and 2 on the CAV807 reference used) (Fig 2). All three candidates were identified in intergenic regions, with Candidate 5 being close to a putative gene. All appeared to be target-specific, as their sequence identities to non-target genome sequences did not exceed 90% in the internal database (Detailed **information of the DNA isolates used in this study.**

S4 Table). In external NCBI BLAST analysis (Step 3, Fig 1), three candidates showed some hits with non-target taxa, all with sequence identities below 84% and with partial regions of the queried sequence (Details in Detailed **information of the DNA isolates used in this study.**

S4 Table). Candidate 5 had a non-specific hit on an *Ascodesmis sphaerospora* strain, Candidate 23 showed non-specific BLAST hits with *F. oxysporum* Fo10, an Australian endophyte isolated from soil (Constantin et al., 2021), and on *F. fujikuroi*. Candidate 27 had hits with a *F. proliferatum* genome.

Fig 2. Genomic locations of the amplicons of previously published Foc-TR4 diagnostic systems used on the genomes of UK0001 and CAV807. The three amplicons of the three candidates described in the current publication in bold. The chromosome names of the UK0001 have been simplified to fit in the Fig, being originally "VMNF010000...".

None of the candidate sequences had BLAST-positive hits on the full number of target Foc TR4 Illumina assemblies used, leading us to a further mapping exploration using the RattleSNP workflow (Step 4, Fig 1). This approach finds alignment coverage and depth of Illumina sequence reads, rather than sequence identity on assemblies, which would allow to explore sequences that had been discarded during assembly process. From all 120 TR4 Illumina genomes, nine and seven genomes were not found to map at all on the candidates 5 and 27, respectively (Table 2**Error! Reference source not found.**). However, since the missing genome lists of these two markers were

complementary to each other, these two markers were kept designing a potential duplex. Candidate 23 on the other hand, was fully inclusive with all 120 TR4 genomes, although mapping depth was low in some regions of the candidate sequence.

Coverage over non-target genomes was below 10% for all the three candidate regions (Full details on Detailed **information of the DNA isolates used in this study.**

S4 Table) except for CAV_36117 (Clade B, Foc-SC04), which mapped with a 12.6% and 43.7% coverage on the sequences of Candidate 5 and 23 respectively, and CAV_612 (Clade A, Foc-SC01) , mapping with a 55.1% of coverage on Candidate 27.

Table 2: ***In silico*** **specificity of candidate Foc-TR4 target sequence regions and LAMP amplicons of those previously published.**

Reference sequence used for alignment / BLAST queries										Genomes used		Internal BLAST results of reference sequences on all assembled genomes (Identity %)			Mapping results of Illumina reads on the Reference Sequences		
Marker Region	AT[a]	ST[a]	L[a]	MC[a]	Mask.[a]	Loc.[a]	Position in CAV807 / UK0001 reference genomes			Status	NTBH[b]	100%	95-100%	90-95%	GFM[c]	GPM[c]	GNM[c]
							Contig	Start	End								
**Cand5**	LAMP	Amplicon	214	No	No	Inter	contig_12 / VMNF01000005.1	1486738 / 5279483	1486951 / 5279270	Target	No	133	0	0	111	0	9
										Non Target		0	0	0	0	2	46
**Cand23**	LAMP	Amplicon	247	No	No	Inter	contig_3 / VMNF01000003.1	4477508 / 4455382	4477754 / 4455628	Target	No	119	0	0	118	2	0
										Non Target		0	0	0	0	10	38
**Cand27**	LAMP	Amplicon	234	No	No	Inter	contig_2 / VMNF01000004.1	32096 / 5203206	32329 / 5202973	Target	Yes	133	0	0	112	1	7
										Non Target		0	0	0	0	7	41
**Ordoñez et al. (2019)**	LAMP	Amplicon	214	No	No	Inter	contig_11 / VMNF01000015.1	623539 / 2103661	623752 / 2103448	Target	Yes	140	0	0	118	0	2
										Non Target		0	**1**	0	**1**	3	44
**Canare et al. (2024)**	LAMP	Amplicon	213	Yes	No	Inter	contig_12 / VMNF01000014.1	842769 / 2814708	842981 / 2814920	Target	Yes	142	0	2	120	0	0
										Non Target		**17**	**2**	17	**17**	5	26
**Li et al. (2013)**	LAMP	Amplicon	213	No	Yes	Genic	contig_6 / VMNF01000005.1	603139 / 613080	603351 / 613292	Target	Yes	143	0	0	120	0	0
										Non Target		**3**	**25**	0	**25**	20	3
**Zhang et al. (2013)**	RT-LAMP	Amplicon	472	Yes	No	Inter	contig_3 / VMNF01000010.1	5266 / 17344	5737 / 16873	Target	Yes	27	4	0	120	0	0
										Non Target		0	**22**	3	**48**	0	0
**Peng et al. (2014)**	RT-LAMP	Amplicon	217	No	Yes	Genic	contig_6 / VMNF01000005.1	603149 / 613090	603365 / 613306	Target	Yes	143	0	0	120	0	0
										Non Target		**3**	**25**	0	**27**	19	2

[a]Summary of the genome locations, BLAST and sequence alignment results. AT: Assay Type; ST: Sequence Type; L: length (bp); MC: Multiple Copies; Mask.: Located in repeat-masked regions; Loc.: location, genic or intergenic (inter)

[b]NTBH: Any Non Target BLAST hit (any size),

[c]GFM: Genomes fully mapped (100% coverage); GPM: genomes partially mapped; GNM: Genomes not mapped (0% coverage).

In silico specificity analysis of candidate regions vs. previously published regions

The approach of internal BLAST and Illumina alignments (Steps 2 and 4 in Fig 1), initially tested on the new candidates, was also applied on already published TR4 diagnostics assays by selecting the amplicon sequences of these systems (Table 2, details on Detailed **information of the DNA isolates used in this study.**

S4 Table). The genomic locations of the different markers on the genomes UK0001 and CAV807 are shown on Fig 2. Considering LAMP systems, none of the published regions were found to be fully specific to our genome panel.

The Ordóñez LAMP amplicon [37] showed the least amount of false positive detections, but had hits of 95-100% sequence identity to eight non-target genomes when tested on the internal BLAST dataset, most notably sequence identity of 97.2% to isolate CAV 225, an *F. oxysporum* isolated soil in banana plantation in South Africa. In addition, the Ordóñez system [37] did not have any sequence alignment with two Foc-TR4 Illumina genomes: SHN19, and FJ41 from China. Considering the other four LAMP systems studied (Table 2), they all showed good inclusivity, by mapping with all 120 Illumina TR4 genomes, but were found to be less specific than Ordóñez *et al.* (2019). The Canare *et al.* 2024 marker [50] [showed 100% sequence identity with 17 non-targeted assembled genomes, most of which were STR4, VCG 0121s, and three Australian unknown VCG Foc isolates. It also showed full alignment with 17 non-target Illumina genomes including various known Foc VCGs (0120, 0121, 0122, 0126, 01215, 01219). The LAMP target reported from [36], designed based on the IGS gene regions [18], showed a 100% alignment with 48 non-target Illumina genomes, and a BLAST identity between 95-100% in 40 non-target alignments, including Foc isolates identified as STR4, Race 1 and isolates in other Foc VCGs. The LAMP amplicons of Li *et al.* (2013), and Peng *et al.* (2014) [19, 35], located nearby close to the amplicon of the RT PCR Lin *et al.* (2013) [51], were found in full alignment within 25 and 27 non-target Illumina genomes, respectively. When analysed

in BLAST both gene targets showed a 100% identity within three VCG 0121 genomes (TWN0005, TWN0004, and TWN0003, and showed a 95-100% identity with 25 non-target genomes.

We also benchmarked other non-LAMP system amplicons (Detailed **information of the DNA isolates used in this study.**

S4 Table). The qPCR amplicon published by Matthews *et al.* 2020, showed the best inclusivity-exclusivity, although it was found to be located in a repeats-rich region of the genome (thus masked by RepeatMasker). The Aguayo qPCR amplicon [20] showed a 100% BLAST identity on the three VCG 0121 genomes (TWN0005, TWN0004, and TWN0003), and high coverage on 47 non-target genomes, mostly with Race 4 isolates.

Primer design and initial validation

A first screening was done on a subset of the specificity DNA panel, confirming that the best candidates were Candidate 5, 23 and 27 (respectively named Cand-5, Cand-23, and Cand-27). LAMP primers were then designed on the three candidates to be tested in the specificity panel.

Due to the limited size of the highly specific part of the Cand-23 fragment, only LAMP primer sets including FIP, BIP, F3, and B3 could be designed using the primer explorer software, and a Loop and two swarm primers [52] designed manually, were included in the final version of the Candidate 23 LAMP assay (

Table **3**, **Error! Reference source not found.**).

Fig 3. Position and direction of the LAMP primers on the Cand-23 region.

Table 3. Primer sequences and concentration used for Cand-23 LAMP assay.

Primer	Sequence	FC[a]
FIP-cand23	AAGCACTTATAAAACCCGGCCTCGAAGTAAACTAACTAGGGCAAC	1.6
BIP-cand23	AAGCTGGCTTAGCCTTAAAAGCTCCTATTAAGGGCAAAGAGAC	1.6
Loop-cand23	TTCTAATTACTTTTATTAGCACTGC	0.8
Swam_F1c-cand23	AAGCACTTATAAAACCCGGCCTC	0.8
SwarmB1c-cand23	AAGCTGGCTTAGCCTTAAAAGC	0.8
F3-cand23	TTAGAGGTATAAGGAGGCTTAG	0.4
B3-cand23	GCCTTTACTTAGTAATCTTTTACCT	0.4

[a]FC: Final concentration (µM), required in the final 25 µL reaction.

LAMP Primer Specificity

The Cand-23 LAMP assay was the most specific one (

), only amplifying true positives and not amplifying any non-target isolates among the 161 strains tested. Cand-5 LAMP assay (121 strains tested) amplified 3 false positives and 7 false negatives, which were some of the genomes found absent *in silico*. This would confirm that their absence was genuine rather than due to sequencing artefacts. Cand-27 showed one 1 false negative (62 strains tested), and therefore, the Cand-23 LAMP assay was chosen for the subsequent tests. Considering other molecular detection tools that were analysed in this study, the Matthews qPCR [21] was found to be fully specific among 104 isolates tested. The Aguayo qPCR [20] amplified all target isolates, and 15 non-target (VCG 0121) isolates. This was not surprising as it is known that this system also amplifies VCG 0121. The Ordoñez LAMP assay [37] (158 isolates tested) picked up all Foc-TR4 target isolates but also amplified 3 non-target strains: CAV 531 (endophyte), CAV 615 (VCG 01227), and Fova1185, a *Fusarium oxysporum* isolated from vanilla, respectively. Detailed results of the specificity are shown in S5 Table.

Table 4. Specificity results of the LAMP markers designed in this project (Cand-23, Cand-5, and Cand-27), compared with three current TR4 diagnostic reference methods.

Isolates tested					Result of different Foc TR4 diagnostic systems											
					Existing systems						This study					
Status	Species[a]	Clade / Foc-SC[b]	VCG[a]	Race[a]	qPCR1[c]		qPCR2[c]		LAMP3[c]		LAMP Cand-23		LAMP Cand-5		LAMP Cand-27	
					+[d]	-[d]	+[d]	-[d]	+[d]	-[d]	+[d]	-[d]	+[d]	-[d]	+[d]	-[d]
Non Target	*F. oxysporum* f. sp. *cubense*	A/1	0120	STR4	0	2	0	2	0	6	0	6	0	3	0	2
			0120/15	UNK	0	3	0	3	0	5	0	5	0	3	0	1
			01210	UNK	0	1	0	1	0	1	0	1	0	1	0	0
			01219	UNK	0	0	0	0	0	1	0	1	0	1	0	1
			0122	UNK	0	2	0	2	0	2	0	2	1	1	0	2
			0129/11	UNK	0	1	0	1	0	2	0	2	0	2	0	1
			01215	UNK	0	0	0	0	0	1	0	1	0	1	0	1
			01227	UNK	0	0	0	0	1	0	0	1	0	1	0	1
			0126	UNK	0	1	0	1	0	1	0	1	0	1	0	1
			0129	UNK	0	0	0	0	0	1	0	1	0	1	0	1
			SMV 1	UNK	0	0	0	0	0	1	0	1	0	1	0	1
			SMV 2	UNK	0	0	0	0	0	1	0	1	0	1	0	1
		A/2	0121	Race 4	8	0	0	8	0	9	0	9	0	9	0	3
Target			01213/16	TR4	23	0	21	0	36	0	37	0	17	7	11	1
			N.D.	TR4*	9	0	9	0	9	0	9	0	0	0	0	0
Non Target			SMV 3	UNK	0	0	0	0	0	1	0	1	1	0	0	1

F. oxysporum f. sp. *cubense*	B/4	01212	Race 1/2	0	1	0	1	0	1	0	1	0	1	0	1
		01220	UNK	0	0	0	0	0	1	0	1	0	1	0	1
		01222	UNK	0	0	0	0	0	1	0	1	0	1	0	1
		0124	UNK	0	2	0	2	0	3	0	3	0	3	0	1
		0124/25	UNK	0	1	0	1	0	1	0	1	0	0	0	0
		0124/5	UNK	0	0	0	0	0	2	0	2	0	2	0	2
		0124/5/8	UNK	0	1	0	1	0	1	0	1	0	1	0	0
		0128	UNK	0	1	0	1	0	3	0	3	0	3	0	2
		ND	UNK	0	3	0	3	0	3	0	3	0	1	0	1
		01228	UNK	0	0	0	0	0	1	0	1	0	1	0	1
		01229	UNK	0	0	0	0	0	1	0	1	0	1	0	1
		01232	UNK	0	0	0	0	0	1	0	1	0	1	0	1
		01233	UNK	0	0	0	0	0	1	0	1	0	1	0	1
		01236	UNK	0	0	0	0	0	1	0	1	0	1	0	1
		0125	UNK	0	1	0	1	0	1	0	1	1	0	0	0
		0128/20	UNK	0	0	0	0	0	1	0	1	0	1	0	1
		SMV 4	UNK	0	0	0	0	0	1	0	1	0	1	0	1
		SMV 5	UNK	0	0	0	0	0	1	0	1	0	1	0	1
		SMV 6	UNK	0	0	0	0	0	1	0	1	0	1	0	1
	B/5	01214	UNK	0	4	0	4	0	5	0	5	0	3	0	1
		01231	UNK	0	0	0	0	0	1	0	1	0	1	0	1
	B/6	0123	Race 1	0	1	0	1	0	2	0	2	0	2	0	2
	B/7	01218	Race 1	0	0	0	0	0	1	0	1	0	1	0	1
		01234	UNK	0	0	0	0	0	1	0	1	0	1	0	1
		01235	UNK	0	0	0	0	0	1	0	1	0	1	0	1
		SMV 10	UNK	0	0	0	0	0	1	0	1	0	1	0	1
	B/8	01230	Race 1	0	0	0	0	0	1	0	1	0	1	0	1
	C/3	01226	Race 1	0	3	0	3	0	3	0	3	0	3	0	3

UNK	Race 1	0	5	0	5	0	5	0	5	0	2	0	0
F. fujikuroi		0	2	0	2	0	2	0	2	0	2	0	0
F. graminearum		0	1	0	1	0	1	0	1	1	0	0	0
F. oxysporum	Endophyte	0	2	0	2	1	2	0	3	0	3	0	2
	UNK	0	7	0	7	0	8	0	8	0	7	0	1
F. oxysporum f. sp. cepae		0	2	0	2	0	2	0	2	0	2	0	0
F. oxysporum f. sp. cyclamini		0	1	0	1	0	1	0	1	0	1	0	0
F. oxysporum f. sp. lini		0	1	0	1	0	1	0	1	0	1	0	0
F. oxysporum f. sp. lycopersici		0	1	0	1	0	1	0	1	0	1	0	0
F. oxysporum f. sp. medicaginis		0	1	0	1	0	1	0	1	0	1	0	0
F. oxysporum f. sp. melonis		0	1	0	1	0	1	0	1	0	1	0	0
F. oxysporum f. sp. radicis-lycopersici		0	1	0	1	0	1	0	1	0	1	0	0
F. oxysporum f. sp. radicisvanilla		0	1	0	1	0	1	0	1	0	1	0	0
F. oxysporum f. sp. vanilla		0	2	0	2	1	1	0	2	0	2	0	0
F. oxysporum f. sp. vasinfectum		0	1	0	1	0	1	0	1	0	1	0	0
F. oxysporum Fo47		0	1	0	1	0	1	0	1	0	1	0	0
F. oxysporum PHRC474		0	1	0	1	0	1	0	1	0	1	0	0
Fusarium f. sp.		0	2	0	2	0	2	0	2	0	0	0	0
Pseudocercospora fijiensis		0	3	0	3	0	3	0	3	0	3	0	0
Ralstonia solanacearum		0	0	0	0	0	0	0	1	0	0	0	0
**Total Counts**		**40**	**64**	**30**	**72**	**48**	**110**	**46**	**114**	**21**	**100**	**11**	**51**

[a] Abbreviations: "F": *Fusarium*, "TR4*": TR4 verified by PCR, "N.D." Not determined, "UNK": Unknown, "SMV": Single-Member VCG.

[b] The Clade and VCG classification is based on Mostert et al., 2022 [27].

[c] qPCR1: Aguayo *et al*., 2017 [20]; qPCR2: Matthews *et al*., 2020 [21]; LAMP3: Ordoñez *et al*., 2019 [37].

[d] Results column meanings: +: Positive, -: Negative.

LAMP Primer Sensitivity and optimisation

Preliminary optimisation of Cand-23 LAMP primer concentrations and combinations allowed the selection of a primer mix (PM) consisting of doubled initial standard concentrations (FIP and BIP at 1.6 µM; SwarmF1c, SwarmB1c and Loop primers at 0.8 µM; and B3 and F3 at 1.4 µM) (Table 3), as this configuration reduced the average TTR by 1 min 45 s (S2 Fig).

Sensitivity of the Cand-23 LAMP assay was tested on plasmid dilutions prepared in water (Fig 4, a) and on DNA extractions of healthy plant tissue spiked with plasmid dilutions (Fig 4, b). In both sample sets, the limit of detection (LOD) was reached consistently at 10^2 copies μL^{-1}. When a volume of 5 µL per reaction of the plasmid DNA was used (blue points in the graphs), TTR averaged 18:07 min, and Annealing temperature (At) of 82.8 °C for plasmid dilutions in water, and average TTR 13:41 min at 82.3 °C in plant tissue. Surprisingly, when sensitivity were conducted on samples were plant tissue were spiked with plasmid dilutions a better detection limit were recorded at 10^1 copies μL^{-1} using 5µL per reaction, that is 50 copies per reaction (average TTR 20:32 min, 82.3 °C in Fig 4, b).

Fig 4. Sensitivity of the Cand-23-LAMP system as assessed in plasmid dilutions (a) and in plasmid-spiked plant tissue extractions (b). TTR: Time To Result (mins:ss). The colour and shape of the data points indicates the DNA volume used in the reaction; blue triangles (5µL) or yellow squares (1µL) for TTR means, while replicates are shown as solid circles. Error bars indicate the Standard Error (SE) of the mean. The horizontal black line indicates the threshold above which a sample is considered negative, and the dashed red horizontal line indicates the value assigned to the samples that produced null signal (negative). The asterisks on top indicate whether the mean is positive with a 95% of confidence level.

Additionally, DNA extractions of conidial dilutions mixed with healthy plant material were tested to compare the Cand-23 LAMP assay with the Ordoñez LAMP assay [37], and two qPCR assays [20, 21]. For both LAMP systems tested, a LOD of 10^5 cells. μL^{-1} were reached. An average TTR at 16:35 min and annealing peak at 82.7 °C were recorded for Cand-23 LAMP system, while for the Ordoñez LAMP assay [37], an average TTR 15:20 min, and annealing peak of recorded at 84.5°C (S3 Fig). Collectively, these results indicated that both LAMP systems display similar sensitivity performances. Considering the qPCR assays, the Matthews *et al*., 2020 SYBR green qPCR could not detect any of the samples from this extraction, whilst the Aguayo system reached a LOD of 10^5 cells. μL^{-1}. The detailed results of all sensitivity tests can be found in S6 Table.

Detection from artificially and naturally infected plant material

In glasshouse conditions, all the healthy and asymptomatic plants tested negative. In the field, the situation was more complex because the presence of other pathogens, particularly the bacterium causing Banana Blood Disease, produced confusing symptoms (yellowing and wilting of leaves, internal vascular discoloration), making the correlation between symptoms and the presence of Foc-TR4 unclear. In Java, Foc-TR4 was detected by both LAMP assays and the qPCR assay in only 10 samples out of 66, which had been collected from seven banana plants of the cultivars Pisang Barangan, Balbisiana, and Pisang Raja, located in Cianjur. A perfect correspondence of results was obtained between the Cand-23 LAMP, and the qPCR results performed on samples from Comoros and Vietnam (Fig 5). Moreover, these results agreed with the VCG determined from the isolated fungi, when available (S7 Table).

The Cohen's kappa index, reflecting the agreement between Cand-23 and qPCR, varied between different datasets (Fig 5a, Table 5 and S7 Table) from 0.65 (substantial) in the glasshouse (n=34), 0.49 (moderate) for in-field sampling done in Indonesia (n=66), up to 1 (perfect) in Comoros (n=8) and Vietnam (n=8). Considering all the field data, the kappa index was 0.64, with a total agreement

of 84.1%, indicating a substantial agreement with the reference method. However, the results obtained from field data between Cand-23 LAMP and qPCR were significantly different (McNemar's test p=0.0265), probably due to the higher positives detected by qPCR compared to LAMP. This discrepancy is reflected in the diagnostics Sensitivity (63.3%) whereas the Specificity shows consistently high values (96.4%). For the glasshouse experiment, results were similar with a total agreement of 82.4%, 0.65 kappa index, but not significant McNemar p-value (0.6831). The glasshouse and Indonesia samples were also tested with Ordóñez LAMP [37] to compare with Cand-23 LAMP (S7 Table and Fig 5b) No significant differences were obtained between the two methods and the different parameters reflected a hight agreement between the two methods: an overall kappa coefficient of 0.87, and sensitivity and specificity values of 89.5% and 97.1%, respectively.

Fig 5. Cand-23 *in planta* result comparison. Comparison of the results obtained with the Cand-23 LAMP assay compared to the results with: (a) the Foc-TR4 specific qPCR as reported by Matthews *et al.*, (2020)[21]and (b) Foc-TR4 specific Ordóñez LAMP assay [37]. The colour of the data points refers the origin of the data (naturally infected samples in Indonesia, Vietnam of Comoros, or artificially inoculated material in the Glasshouse) and indicates the average of the different replicates.

Table 5: Summary of the results obtained by the Cand-23 LAMP compared to qPCR assay as published by Matthews *et al*. (2020).

	Field_Comoros		Field_Indonesia		Field_Vietnam		Glasshouse		TOTAL FIELD		TOTAL	
	LAMP +	LAMP -	LAMP +	LAMP -	LAMP +	LAMP -	LAMP +	LAMP -	LAMP +	LAMP -	LAMP +	LAMP -
**qPCR +**	6	0	10	11	3	0	15	4	19	11	34	15
**qPCR -**	0	2	2	43	0	5	2	13	2	50	4	63
**n. Samples**	6	2	12	54	3	5	17	17	21	61	38	78
**Total n. Samples**	8		66		8		34		82		116	
**Pos. agreement**	100%		83.3%		100%		88.2%		90.5%		89.5%	
**Neg. agreement**	100%		79.6%		100%		76.5%		82%		80.8%	
**Total agreement**	100%		80.3%		100%		82.4%		84.1%		83.6%	
**Cohen's kappa (± SD)**	**1 (±0)**		**0.49 (±0.12)**		**1 (±0)**		**0.65 (±0.13)**		**0.64 (±0.09)**		**0.65 (±0.07)**	
**McNemar's Chi-sq.**			4.92				0.17		4.92		5.26	
**McNemar's p-value (Chi-sq.)**			0.0265				0.6831		0.0265		0.0218	
**Sensitivity (SE)**	100%		47.6%		100%		78.9%		63.3%		69.4%	
**Specificity (SP)**	100%		95.6%		100%		86.7%		96.2%		94%	
**Accuracy (AC)**	100%		80.3%		100%		82.4%		84.1%		83.6%	
**Positive Predictive Value (PPV)**	100%		83.3%		100%		88.2%		90.5%		89.5%	
**Negative Predictive Value (NPV)**	100%		79.6%		100%		76.5%		82%		80.8%	

Results are presented for artificially inoculation conducted under glasshouse conditions, as well as naturally infected samples analysed collected in Indonesia, Comoros, and Vietnam. Parameter description: Sensitivity (SE) = TP / (TP + FN): proportion of true positives correctly detected by LAMP. Specificity (SP) = TN / (TN + FP): proportion of true negatives correctly identified. Positive Predictive Value (PPV) = TP / (TP + FP): probability that a positive LAMP result is a true positive. Negative Predictive Value (NPV) = TN / (TN + FN): probability that a negative LAMP result is a true negative. Accuracy (AC) = (TP + TN) / (TP + TN + FP + FN): overall proportion of correctly classified samples. Cohen's Kappa ≤ 0 corresponds to no agreement, 0.01–0.20 is "none to slight", 0.21–0.40 is "fair", 0.41– 0.60 is "moderate", 0.61–0.80 is "substantial", and 0.81–1.00 is "near-perfect". A McNemar's p

value below 0.05 was taken as evidence to reject the null hypothesis of equal marginal proportions, indicating a statistically significant difference in detection outcomes between the two methods compared.

Discussion

Foc-TR4 continues to expand globally, with latest detections reported in Latin America (Colombia, Peru and Venezuela) [14] and Africa (Mayotte [53], and Comoros islands [54]). The continued spread poses a significant threat to the global banana industry due to the lack of effective curative measures against the disease once established. Consequently, there is an urgent need for a rapid and reliable diagnostic tool for early detection to implement timely containment strategies and mitigate the impact of the disease. In this study, we present a new real-time LAMP assay for Foc-TR4 VCG 01213/16 identification, which were robustly designed through comparative genomic approach and specificity testing on a diverse collection of 148 Foc-TR4 genomes, and 146 non-target genomes, including all known Foc VCGs and other closely *F. oxysporum* as well as other genera strains. This assay can be applied directly in-field and therefore, offer an improved tool that can be used for early detection in surveillance efforts or in containment strategies on-farm.

In this study the K-mer Elimination by Cross-reference (KEC) method [38]was considered for robust identification of a Foc TR4 specific sequence region. The KEC method has previously been used to develop LAMP assays in plant pathogenic bacteria and fungi [55, 56], but not yet on *Fusarium oxysporum*. The KEC method enables unbiased, genome-wide identification of TR4-specific sequences by operating directly at the k-mer level, independently of gene annotation or pre-defined loci, thereby reducing reference bias compared with gene- or reference-based approaches. In addition, KEC allows the direct selection of candidate regions of a predefined minimum length, which is essential for LAMP primer design, where recommended target sizes range between 120 and 180 nucleotides [33]. This contrasts with other approaches used so far to develop Foc TR4 diagnostic assays, such as DArTseq sequencing which implies genome complexity reduction [21, 37], or the identification of polymorphisms in known *SIX* genes [50, 57], which are biased towards enzyme restrictions sites or genes, and yield short candidate sequences. These sequences often require to be extended to be able to design LAMP primers, at the risk of losing the specificity. Several alternative

marker discovery strategies were evaluated in this study, including ortho-group filtering and Illumina read mapping and filtering against a TR4 reference genome, but these approaches yielded few candidates and lacked sufficient specificity or inclusivity. In contrast, the KEC method generated multiple candidate regions, enabling downstream filtering and experimental validation to select a robust marker. Notwithstanding this advantage, the KEC method may also be limited by the quality of the input genome assemblies used, which might include poor genomic sequences such as high GC content regions, not appropriate for primer design. To overcome these limitations the reference genome (referred as “master” genome in KEC protocol) used was a high quality long-read assembly of an Foc-TR4 isolate. The repetitive element-masked version of the genome was used to avoid the selection of candidates within the repeat-rich regions of the genome which could potentially be unstable. One disadvantage of the KEC approach employed however, was that not all the predicted sequences were target specific, as BLAST searches showed matches in non-target genomes. The initial version (v1.0) of the KEC software was used in this analysis, in which the *include* mode applied less stringent criteria for sequence uniqueness than in later releases, potentially allowing retention of sequences not exclusively specific to the target. This limitation was nevertheless addressed by the addition of internal and external BLAST steps improved specificity, but full inclusivity on all the target Foc-TR4 genomes was still not achieved. This was probably due to the incompleteness of some Illumina assemblies, as regions with low read depth and quality were excluded during the assembly process [58]. Adding the Illumina read mapping step allowed to identify genuine genomic absences on certain Foc-TR4 isolates in some of the candidates, leading the selection of the Candidate 23, which was present in all 120 target genomes and absent in all non-target genomes representing all known *Foc* VCGs, as well as non-pathogenic and endophytic *F. oxysporum* species associated with banana. This multi-step validation demonstrated that the KEC-based strategy, combined with downstream filtering, provided a clear improvement in marker specificity and inclusivity compared to alternative approaches cited above. This was demonstrated experimentally when tested on a large specificity panel comprising Foc-TR4 isolates representatives from all known other *Foc* VCGs,

additional isolates belonging to the *F. oxysporum* species complex and fungal endophytes isolates from banana environment

The selected Candidate 23 is located within a unique intergenic region on Chromosome 2 of the reference II5 (GCA 031834405). The sequence was characterized by the presence of repetitive nucleotides. Additionally, read coverage was low, with a pronounced decrease observed in one part of the sequence. Due to the presence of repetitive nucleotides in the Candidate 23 target sequence, the design of LAMP primers with primer design software was not possible. This issue was resolved through the manual design of a loop primer and Swarm primers, a lesser-known alternative to loop primers that enhanced the LAMP reaction [52, 59]. This allowed the development of a LAMP system that worked and provided a clear single annealing peak.

This study also analysed the specificity of other already published Foc-TR4 LAMP assays using the *in silico* approach. Amplicon sequences from Li *et al.* (2013) [19] showed good inclusivity in terms of BLAST and alignment on all Foc-TR4 Illumina genomes, but also had high (95-100%) identity and full alignment with 43 and 25 non-target genomes respectively, including 100% BLAST identity on three VCG 0121 genomes. A similar result was obtained with the LAMP assay designed by Canare *et al*. (2024) [50], consistent with the fact that this LAMP targets the *SIX8a* homologue gene, which is present in all Race 4 isolates [28, 60]. In contrast, the Ordóñez *et al.* (2019) LAMP assay [37]was not fully inclusive, not picking up two Foc TR4 genomes (both of Chinese origin). Whether this was a genuine lack or could have been caused by sequencing problems of these genomes, could not be validated on DNA; however, the correlation between the *in silico* and *in vitro* was good in the sixty-two isolates for which there was data and DNA. Also, similar cases of missing alignments on genomes for Candidate 5 were validated experimentally on seven DNA isolates. On the other hand, this LAMP system was not fully exclusive, showing high sequence homology to an *F. oxysporum* endophyte, and positives on non-target DNA isolates. The latter result stresses the importance of performing thorough

specificity testing especially considering endophytic, non-pathogenic isolates which might amplify as false positives. This is particularly challenging in fungal species with a broad host range such as *F.* o*xysporum* given the genetic similarity between non-pathogenic and endophytic isolates with pathogenic isolates [61].

The sensitivity of our LAMP assay obtained a consistent LOD of up to 100 copies per reaction with plasmid dilutions, which is the same dilution as reported in other existing LAMP systems for Foc-TR4 [40]. Interestingly a better LOD of 50 copies per reaction was reached when testing the plasmid dilutions in a plant tissue background extraction. Plant tissue providing a matrix which would help plasmid DNA precipitation and binding, preventing degradation and DNA loss could possibly explain this difference with the tests carried out on simple water plasmid dilutions [62, 63]. Nevertheless, this corroborates the fact that non-target DNA presence does not affect the assay performance.

The LAMP assay designed in this study was evaluated under glasshouse and field conditions across three countries (Indonesia, Vietnam, and Comoros), and it performed reliably using both commercial and simplified DNA extraction protocols. The tool's portability was also demonstrated through successful implementation in non-laboratory settings, highlighting its potential for use in resource-limited settings.
In comparison with the qPCR assays tested in parallel, our LAMP assay showed strong correlation; however, qPCR still proved to remain as the gold standard in terms of sensitivity. Nevertheless, this came with the trade-off of higher requirements in terms of laboratory, extraction equipment and time.

Conclusion

Extensive regulatory measures have been implemented to prevent the introduction and establishment of Foc-TR4 in unaffected areas. The effectiveness of this surveillance relies heavily on having

specific and sensitive detection protocols. Although molecular-based methods are available, many have demonstrated issues with specificity. A novel highly specific LAMP assay was developed in this study for in-field detection of Foc-TR4, which was validated by comparative genomics approach. This approach accounts for the known genetic diversity within Foc and FOSC, improving upon the specificity of previously published Foc-TR4 detection assays to date. The LAMP assay was shown to be highly effective in detecting FocTR4 in infected banana plants, particularly in non-laboratory environments, offering a promising tool for improving disease management strategies. Implementing this technique regularly in diagnostic processes will allow early identification of infected banana plants, facilitating timely intervention to prevent widespread outbreaks. The Cand-23 LAMP assay is now commercialized in a ready-to-use “LAMP Foc-TR4 kit” produced by QUALIPLANTE SAS (Lavérune, France. https://www.qualiplante.eu), in an all-inclusive format containing the lyophilized primer mix, the lyophilized enzyme, a resuspensions buffer, and positive and negative controls.

Acknowledgements

We thank Natalya Saveljeva, Veronique Roussel and Christine Pages for technical assistance, Lorène Milliex for the project management, Caroline Chatillon for assistance in Qualiplante, Megan Matthews for help in extractions and assistance. We also thank Nguyen Thi Thô for assistance and extractions, and Mauricio Soto-Suarez (Agrosavia) for having shared Foc-TR4 genome assemblies and long-reads ONT data from Colombia.

References

1. FAO. Together, we can prevent the spread of Tropical Race 4 (TR4). Food Agric Organ United Nations2020. p. 1-8.
2. Viljoen A, Ma L-J, Molina AB. CHAPTER 8: Fusarium Wilt (Panama Disease) and Monoculture in Banana Production: Resurgence of a Century-Old Disease. In: Ristaino JB, Records A, editors. Emerging Plant Diseases and Global Food Security. Saint Paul, MN, USA: APS Press; 2020. p. 159-84.
3. Munhoz T, Vargas J, Teixeira L, Staver C, Dita M. Fusarium Tropical Race 4 in Latin America and the Caribbean: status and global research advances towards disease management. Frontiers in Plant Science [Internet]. 2024 2024-July-16; 15: 1397617. Available from: https://www.frontiersin.org/journals/plant-science/articles/10.3389/fpls.2024.1397617/pdf.
4. Stover RH. Fusarial wilt (panama disease) of bananas and other Musa species: Commonwealth Mycological Institute; 1962. 117 p.
5. Ploetz RC. Fusarium Wilt of Banana Is Caused by Several Pathogens Referred to as *Fusarium oxysporum* f. sp. *cubense*. Phytopathology. 2006;96(6):653-6. doi: 10.1094/PHYTO-96-0653. PubMed PMID: 18943184.
6. Lescot T. Inquiétudes sur l'évolution géographique de la race 4 tropicale (TR4) de la maladie de Panama [Concerns over the geographic spread of tropical race 4 (TR4) of Panama disease]. Fruitrop. 2015;(231):68. PubMed PMID: Agritrop:581003; PubMed Central PMCID: PMCAxe 4 (2014-2018) - Santé des animaux et des plantes.
7. Dawson C, Loeillet D, Lescot T, Sanchez C. Recueil statistique Banane 2023. Document technique et de recherche. Montpellier, France: CIRAD, 2024 Contract No.: Agritrop:611936.
8. Voora V, Larrea C, Bermúdez S. Global Market Report: Bananas. International Institute for Sustainable Development, 2020.

9. Drenth A, Kema G. The Vulnerability of Bananas to Globally Emerging Disease Threats. Phytopathology. 2021;111(12):2146-61. doi: 10.1094/phyto-07-20-0311-rvw. PubMed PMID: 34231377.

10. Ploetz RC. Panama disease: Return of the first banana menace. Int J Pest Manage. 1994;40(4):326-36. doi: 10.1080/09670879409371908.

11. Dita M, Barquero M, Heck D, Mizubuti ESG, Staver CP. Fusarium Wilt of Banana: Current Knowledge on Epidemiology and Research Needs Toward Sustainable Disease Management. Frontiers in Plant Science [Internet]. 2018 Oct 19; 9: 1468. Available from: https//doi.org/10.3389/fpls.2018.01468.

12. Waraczewski R, Sołowiej BG. May bananas go extinct? The epidemic of Panama disease. A review. Agronomy Science. 2022;77(3):139-48. doi: 10.24326/as.2022.3.10.

13. Viljoen A, Mostert D, Chiconela T, Beukes I, Fraser C, Dwyer J, et al. Occurrence and spread of the banana fungus *Fusarium oxysporum* f. sp. *cubense* TR4 in Mozambique. S Afr J Sci [Internet]. 2020 11/26; 116(11/12). Available from: https://sajs.co.za/article/view/8608.

14. Martínez G, Olivares BO, Rey JC, Rojas J, Cardenas J, Muentes C, et al. The Advance of Fusarium Wilt Tropical Race 4 in Musaceae of Latin America and the Caribbean: Current Situation. Pathogens [Internet]. 2023 Feb; 12(2): 277. Available from: https://10.3390/pathogens12020277.

15. Maymon M, Sela N, Shpatz U, Galpaz N, Freeman S. The origin and current situation of *Fusarium oxysporum* f. sp. *cubense* tropical race 4 in Israel and the Middle East. Sci Rep [Internet]. 2020 Jan 31; 10(1): 1590. Available from: https://www.nature.com/articles/s41598-020-58378-9.pdf.

16. Sharrock S, Frison E. Musa production around the world - trends, varieties and regional importance. Focus Paper 2. Montpellier, France: INIBAP, 1998.

17. Pegg KG, Coates LM, O'Neill WT, Turner DW. The Epidemiology of Fusarium Wilt of Banana. Frontiers in Plant Science [Internet]. 2019; 10: 1395. Available from: https://doi.org/10.3389/fpls.2019.01395.

18. Dita MA, Waalwijk C, Buddenhagen IW, Souza MT, Kema GHJ. A molecular diagnostic for tropical race 4 of the banana fusarium wilt pathogen. Plant Pathology. 2010;59(2):348-57. doi: 10.1111/j.1365-3059.2009.02221.x. PubMed PMID: WOS:000275107800015.

19. Li BJ, Du JL, Lan CZ, Liu PQ, Weng QY, Chen QH. Development of a loop-mediated isothermal amplification assay for rapid and sensitive detection of *Fusarium oxysporum* f. sp c*ubense* race 4. Eur J Plant Pathol. 2013;135(4):903-11. doi: 10.1007/s10658-012-0136-9. PubMed PMID: WOS:000315601500026.

20. Aguayo J, Mostert D, Fourrier-Jeandel C, Cerf-Wendling I, Hostachy B, Viljoen A, et al. Development of a hydrolysis probe-based real-time assay for the detection of tropical strains of *Fusarium oxysporum* f. sp. *cubense* race 4. Plos One [Internet]. 2017; 12(2): e0171767. Available from: https://www.ncbi.nlm.nih.gov/pmc/articles/PMC5298334/pdf/pone.0171767.pdf.

21. Matthews MC, Mostert D, Ndayihanzamaso P, Rose LJ, Viljoen A. Quantitative detection of economically important *Fusarium oxysporum* f. sp. *cubense* strains in Africa in plants, soil and water. Plos One [Internet]. 2020; 15(7): e0236110. Available from: https://journals.plos.org/plosone/article/file?id=10.1371/journal.pone.0236110&type=printable.

22. Roberts JM, Carvalhais LC, O'Dwyer C, Rincón-Flórez VA, Drenth A. Diagnostics of Fusarium wilt in banana: Current status and challenges. Plant Pathology. 2024;73(4):760-76. doi: https://doi.org/10.1111/ppa.13863.

23. Ma LJ, van der Does HC, Borkovich KA, Coleman JJ, Daboussi MJ, Di Pietro A, et al. Comparative genomics reveals mobile pathogenicity chromosomes in Fusarium. Nature

[Internet]. 2010 Mar 18 PMC3048781]; 464(7287). Available from: https://www.nature.com/articles/nature08850.pdf.

24. Magdama F, Monserrate-Maggi L, Serrano L, Sosa D, Geiser DM, Jimenez-Gasco MDM. Comparative analysis uncovers the limitations of current molecular detection methods for *Fusarium oxysporum* f. sp. *cubense* race 4 strains. Plos One [Internet]. 2019; 14(9): e0222727. Available from: https://journals.plos.org/plosone/article/file?id=10.1371/journal.pone.0222727&type=printable.

25. O'Donnell K, Sutton DA, Rinaldi MG, Magnon KC, Cox PA, Revankar SG, et al. Genetic diversity of human pathogenic members of the *Fusarium oxysporum* complex inferred from multilocus DNA sequence data and amplified fragment length polymorphism analyses: evidence for the recent dispersion of a geographically widespread clonal lineage and nosocomial origin. J Clin Microbiol. 2004;42(11):5109-20. Epub 2004/11/06. doi: 10.1128/JCM.42.11.5109-5120.2004. PubMed PMID: 15528703; PubMed Central PMCID: PMCPMC525153.

26. O'Donnell K, Kistler HC, Cigelnik E, Ploetz RC. Multiple evolutionary origins of the fungus causing Panama disease of banana: concordant evidence from nuclear and mitochondrial gene genealogies. Proc Natl Acad Sci U S A. 1998;95(5):2044-9. PubMed PMID: 9482835; PubMed Central PMCID: PMCPMC19243.

27. Mostert D, Wicker E, de Jager MM, Al Kaabi SM, O'Neill WT, Perry S, et al. A Polyphasic Approach Reveals Novel Genotypes and Updates the Genetic Structure of the Banana Fusarium Wilt Pathogen. Microorganisms [Internet]. 2022; 10(2): 269. Available from: https://www.mdpi.com/2076-2607/10/2/269.

28. Czislowski E, Fraser-Smith S, Zander M, O'Neill WT, Meldrum RA, Tran-Nguyen LTT, et al. Investigation of the diversity of effector genes in the banana pathogen, *Fusarium*

oxysporum f. sp. *cubense*, reveals evidence of horizontal gene transfer. Mol Plant Pathol. 2018;19(5):1155-71. doi: 10.1111/mpp.12594. PubMed PMID: 28802020.

29. Zheng S-J, García-Bastidas F, Li X, Zeng L, Bai T, Xu S, et al. New Geographical Insights of the Latest Expansion of *Fusarium oxysporum* f.sp. *cubense* Tropical Race 4 Into the Greater Mekong Subregion. Front Plant Sci [Internet]. 2018; 9: 457. Available from: https://doi.org/10.3389/fpls.2018.00457.

30. Torres Bedoya E, Bebber DP, Studholme DJ. Taxonomic Revision of the Banana Fusarium Wilt TR4 Pathogen Is Premature. Phytopathology. 2021;111(12):2141-5. doi: 10.1094/phyto-03-21-0089-le. PubMed PMID: 34100303.

31. Maryani N, Lombard L, Poerba YS, Subandiyah S, Crous PW, Kema GHJ. Phylogeny and genetic diversity of the banana Fusarium wilt pathogen *Fusarium oxysporum* f. sp. *cubense* in the Indonesian centre of origin. Stud Mycol [Internet]. 2019 Mar; 92. Available from: https://www.ncbi.nlm.nih.gov/pmc/articles/PMC6086327/pdf/main.pdf.

32. EFSA PoPH, Bragard C, Baptista P, Chatzivassiliou E, Di Serio F, Gonthier P, et al. Pest categorisation of *Fusarium oxysporum* f. sp. *cubense* Tropical Race 4. EFSA Journal [Internet]. 2022; 20(1): e07092. Available from: https://efsa.onlinelibrary.wiley.com/doi/abs/10.2903/j.efsa.2022.7092.

33. Yang L, Sun Y, Sun L, Wang Z, Feng J, Liang Y. Application of Loop-Mediated Isothermal Amplification in Plant Pathogen Detection. Phytopathology. 2025;115(1):6-13. doi: 10.1094/phyto-10-23-0391-kc. PubMed PMID: 39348563.

34. Németh MZ, Kovács GM. A comprehensive guide to loop-mediated isothermal amplification, an emerging diagnostic tool for plant pathogenic fungi. Frontiers in Plant Science [Internet]. 2025 2025-May-22; 16: 1568657. Available from: https://www.frontiersin.org/journals/plant-science/articles/10.3389/fpls.2025.1568657.

35. Peng J, Zhang H, Chen F, Zhang X, Xie Y, Hou X, et al. Rapid and quantitative detection of *Fusarium oxysporum* f. sp. *cubense* race 4 in soil by real-time fluorescence loop-mediated

isothermal amplification. J Appl Microbiol. 2014;117(6):1740-9. doi: 10.1111/jam.12645. PubMed PMID: 25200557.

36. Zhang X, Zhang H, Pu JJ, Qi YX, Yu QF, Xie YX, et al. Development of a Real-Time Fluorescence Loop-Mediated Isothermal Amplification Assay for Rapid and Quantitative Detection of *Fusarium oxysporum* f. sp *cubense* Tropical Race 4 In Soil. Plos One [Internet]. 2013 Dec 20; 8(12): e82841. Available from: https://doi.org/10.1371/journal.pone.0082841.

37. Ordóñez N, Salacinas M, Mendes O, Seidl MF, Meijer HJG, Schoen CD, et al. A loop-mediated isothermal amplification (LAMP) assay based on unique markers derived from genotyping by sequencing data for rapid in planta diagnosis of Panama disease caused by Tropical Race 4 in banana. Plant Pathology. 2019;68(9):1682-93. doi: 10.1111/ppa.13093.

38. Beran P, Stehlíková D, Cohen SP, Čurn V. KEC: unique sequence search by K-mer exclusion. Bioinformatics [Internet]. 2021; 37(19): btab196. Available from: https://doi.org/10.1093/bioinformatics/btab196.

39. Bache S. Analysis of the genomic structures of several strains of the fungal plant pathogen *Fusarium oxysporum* f.sp. *cubense* [Master]. Montpellier, Strasbourg: Université de Strasbourg; 2022.

40. Mestiri Y. Outils d'annotation et de visualisation de structure génomique pour l'annotation et l'exploration du pangénome fongique. Application à *Fusarium oxysporum* f.sp. *cubense*, agent causal de la fusariose du bananier. Montpellier: Université de Montpellier; 2023.

41. Jackman SD, Vandervalk BP, Mohamadi H, Chu J, Yeo S, Hammond SA, et al. ABySS 2.0: resource-efficient assembly of large genomes using a Bloom filter. Genome Research. 2017;27(5):768-77. doi: 10.1101/gr.214346.116. PubMed PMID: WOS:000400392400011.

42. Robène I, Maillot-Lebon V, Pecrix Y, Ravelomanantsoa S, Massé D, Jouen E, et al. Loop-Mediated Isothermal Amplification Assays for Rapid Detection of

the *Ralstonia solanacearum* Species Complex and Phylotype I in Solanaceous Crops. Phytofrontiers. 2023;3(3):582-93. doi: 10.1094/Phytofr-08-22-0089-R. PubMed PMID: WOS:001369256500020.

43. ANSES. Détection des souches tropicales de *Fusarium oxysporum* f. sp. *cubense* race 4 (Foc TR4) par PCR en temps réel. Méthode d'analyse en santé des végétaux: ANSES-Laboratoire de la Santé des Végétaux, Laboratoire de référence "Champignons sur toute matrice"; 2018. p. 27.

44. Kelman A. The relationship of pathogenicity in *Pseudomonas solanacearum* to colony appearance on a tetrazolium medium. Phytopathology. 1954;44:693-5.

45. Matthews MC. The global spread and detection of *Fusarium oxysporum* f. sp. *cubense* tropical Race 4 [PhD]. Stellenbosch: Stellenbosch University; 2023.

46. Matthews MC, van der Linden J, Robène I, Rozsasi S, Coetzee B, Campa M, et al. A combined recombinase polymerase amplification CRISPR/Cas12a assay for detection of *Fusarium oxysporum* f. sp. *cubense* tropical race 4. Sci Rep-Uk [Internet]. 2025 2025/01/19; 15(1): 2436. Available from: https://doi.org/10.1038/s41598-025-85633-8.

47. Wanjala BW, Ateka EM, Miano DW, Fuentes S, Perez A, Low JW, et al. Loop-Mediated Isothermal Amplification assays for on-site detection of the main sweetpotato infecting viruses. J Virol Methods [Internet]. 2021 2021/12/01; 298: 114301. Available from: https://www.sciencedirect.com/science/article/pii/S0166093421002408.

48. McHugh ML. Lessons in biostatistics, Interrater reliability: the kappa statistic. Biochem Med (Zagreb). 2012;22(3):276-82. PubMed PMID: 23092060; PubMed Central PMCID: PMCPMC3900052.

49. Cao G, Lin K, Ai J, Cai J, Zhang H, Yu Y, et al. A diagnostic accuracy study comparing RNA LAMP, direct LAMP, and rapid antigen testing from nasopharyngeal swabs. Front Microbiol [Internet]. 2022 [cited The authors declare that the research was conducted in the absence of any commercial or financial relationships that could be construed as a potential conflict of

interest. PMC9813509]; 13: 1063414. Available from: https://doi.org/10.3389/fmicb.2022.1063414.

50. Canare Perry Lorraine D, Uy Roselyn J, Konishi S, Tonami H, Alvindia Dionisio G, Nakashima C. Development of Loop-Mediated Isothermal Amplification (LAMP) Kit for the Detection of Fusarium Wilt Using *SIX8* Genes. J Phytopathol [Internet]. 2024; 172(5): e13398. Available from: https://onlinelibrary.wiley.com/doi/abs/10.1111/jph.13398.

51. Lin YH, Su CC, Chao CP, Chen CY, Chang CJ, Huang JW, et al. A molecular diagnosis method using real-time PCR for quantification and detection of *Fusarium oxysporum* f. sp *cubense* race 4. Eur J Plant Pathol. 2013;135(2):395-405. doi: 10.1007/s10658-012-0096-0. PubMed PMID: WOS:000313163400016.

52. Botella JR. Point-of-Care DNA Amplification for Disease Diagnosis and Management. Annu Rev Phytopathol [Internet]. 2022; 60(1). Available from: https://www.annualreviews.org/doi/abs/10.1146/annurev-phyto-021621-115027.

53. Aguayo J, Cerf I, Folscher AB, Fourrier-Jeandel C, Ioos R, Matthews MC, et al. First report of *Fusarium oxysporum* f. sp. *cubense* tropical race 4 (TR4) causing banana wilt in the Island of Mayotte. Plant Dis. 2021;105(1):219. Epub 2020/08/07. doi: 10.1094/PDIS-06-20-1196-PDN. PubMed PMID: 32755365.

54. Mmadi M, Azali HA, Mostert D, Robène I, Viljoen A. First Report of Fusarium Wilt of Cavendish Bananas Caused by *Fusarium oxysporum* f. sp. *cubense* Tropical Race 4 in the Grande Comoros Island. Plant Disease. 2023;107(12):4029. doi: 10.1094/pdis-07-23-1288-pdn. PubMed PMID: 37526488.

55. Beran P, Stehlikova D, Cohen SP, Rost M, Beranova K, Curn V. Utilization of a New Hundred-Genomes Pipeline to Design a Rapid Duplex LAMP Detection Assay for *Xanthomonas euvesicatoria* and *X. vesicatoria* in Tomato. Plant Dis. 2023;107(6):1822-8. Epub 20230601. doi: 10.1094/PDIS-05-22-1098-RE. PubMed PMID: 36428255.

56. Wang Z, Guo L, Tan X, Deng J, Gong S, Li D, et al. Development of Loop-Mediated Isothermal Amplification Assays for the Rapid and Accurate Diagnosis of *Exserohilum turcicum* for Field Applications. Plant Disease. 2024;108(6):1461-9. doi: 10.1094/pdis-10-23-2101-sr. PubMed PMID: 38240714.

57. Carvalhais LC, Henderson J, Rincon-Florez VA, O'Dwyer C, Czislowski E, Aitken EAB, et al. Molecular Diagnostics of Banana Fusarium Wilt Targeting Secreted-in-Xylem Genes. Front Plant Sci [Internet]. 2019 2019/06/20 PMC6554419]; 10: 547. Available from: https://www.ncbi.nlm.nih.gov/pmc/articles/PMC6554419/pdf/fpls-10-00547.pdf.

58. Lischer HEL, Shimizu KK. Reference-guided *de novo* assembly approach improves genome reconstruction for related species. BMC Bioinformatics [Internet]. 2017 Nov 10; 18(1): 474. Available from: https://link.springer.com/content/pdf/10.1186/s12859-017-1911-6.pdf.

59. Martineau RL, Murray SA, Ci S, Gao W, Chao S-h, Meldrum DR. Improved Performance of Loop-Mediated Isothermal Amplification Assays via Swarm Priming. Analytical Chemistry. 2017;89(1):625-32. doi: 10.1021/acs.analchem.6b02578.

60. Fraser-Smith S, Czislowski E, Meldrum RA, Zander M, O'Neill W, Balali GR, et al. Sequence variation in the putative effector gene SIX8 facilitates molecular differentiation of *Fusarium oxysporum* f. sp *cubense*. Plant Pathology. 2014;63(5):1044-52. doi: 10.1111/ppa.12184. PubMed PMID: WOS:000342808300009.

61. Lievens B, Rep M, Thomma BP. Recent developments in the molecular discrimination of *formae speciales* of *Fusarium oxysporum*. Pest Manag Sci. 2008;64(8):781-8. doi: 10.1002/ps.1564. PubMed PMID: 18335459.

62. Zhu B. Degradation of plasmid and plant DNA in water microcosms monitored by natural transformation and real-time polymerase chain reaction (PCR). Water Res. 2006;40(17):3231-8. Epub 20060901. doi: 10.1016/j.watres.2006.06.040. PubMed PMID: 16945402.

63. Si NW, Li J, Gao HF, Li YJ, Zhai SS, Xiao F, et al. Effect of the Matrix and Target on the Accurate Quantification of Genomic and Plasmid DNA by Digital Polymerase Chain

Reaction. Agriculture-Basel [Internet]. 2023 Jan; 13(1): 127. Available from: https://doi.org/10.3390/agriculture13010127.

Supporting information

S1 Methods. DNA extraction protocol.

S1 Fig: Plasmid diagram

S2 Fig: Primer optimisation recipe comparison test

S3 Fig: Sensitivity results on conidia dilutions-spiked plant tissue

S1 Table: Detailed information of the genomic data used in this study.

S2 Table: Detailed parameters of the KEC candidates and their derived primers.

S3 Table: Detailed information of the DNA isolates used in this study.

S4 Table: S4_Table. Detailed informations on the LAMP candidates as compared with LAMP and PCR/qPCR assays already published.

S5 Table: Detailed Specificity Results of our LAMP candidates compared with qPCRs and LAMP references.

S6 Table: Detailed Sensitivity Results of our LAMP candidates compared with qPCRs and LAMP references.

S7 Table: Detailed TR4 Diagnostic results of test done on plants of our LAMP candidates compared with qPCR and LAMP references.

Figure1. Pipeline followed to find TR4-specific sequences.

Click here to access/download;Figure;Fig1.tiff

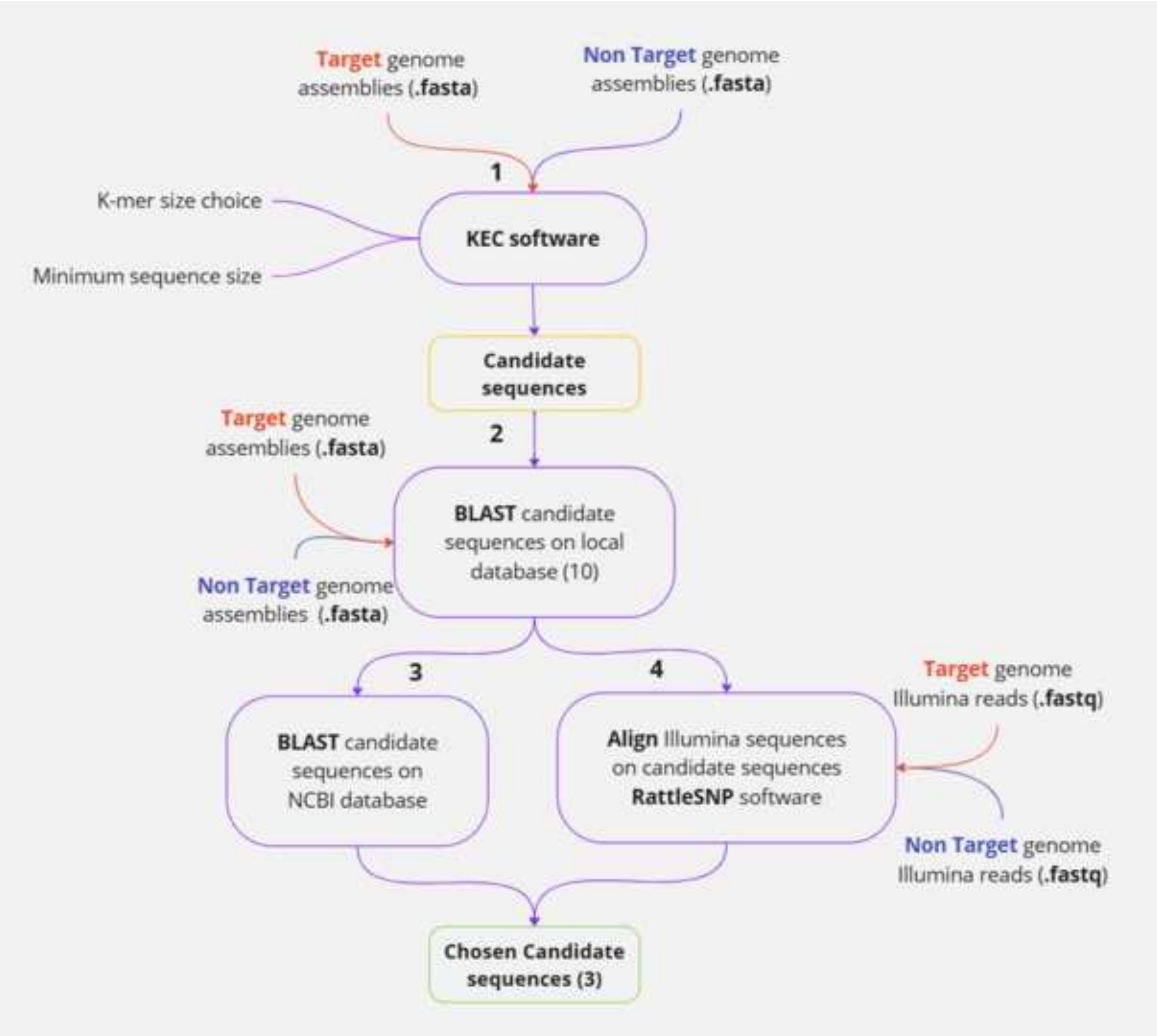

Figure2.Genomic locations of the amplicons of previously published Foc-TR4 diagnostic systems used on the genomes of UK0001 and CAV807.

Click here to access/download;Figure;Fig2.tiff

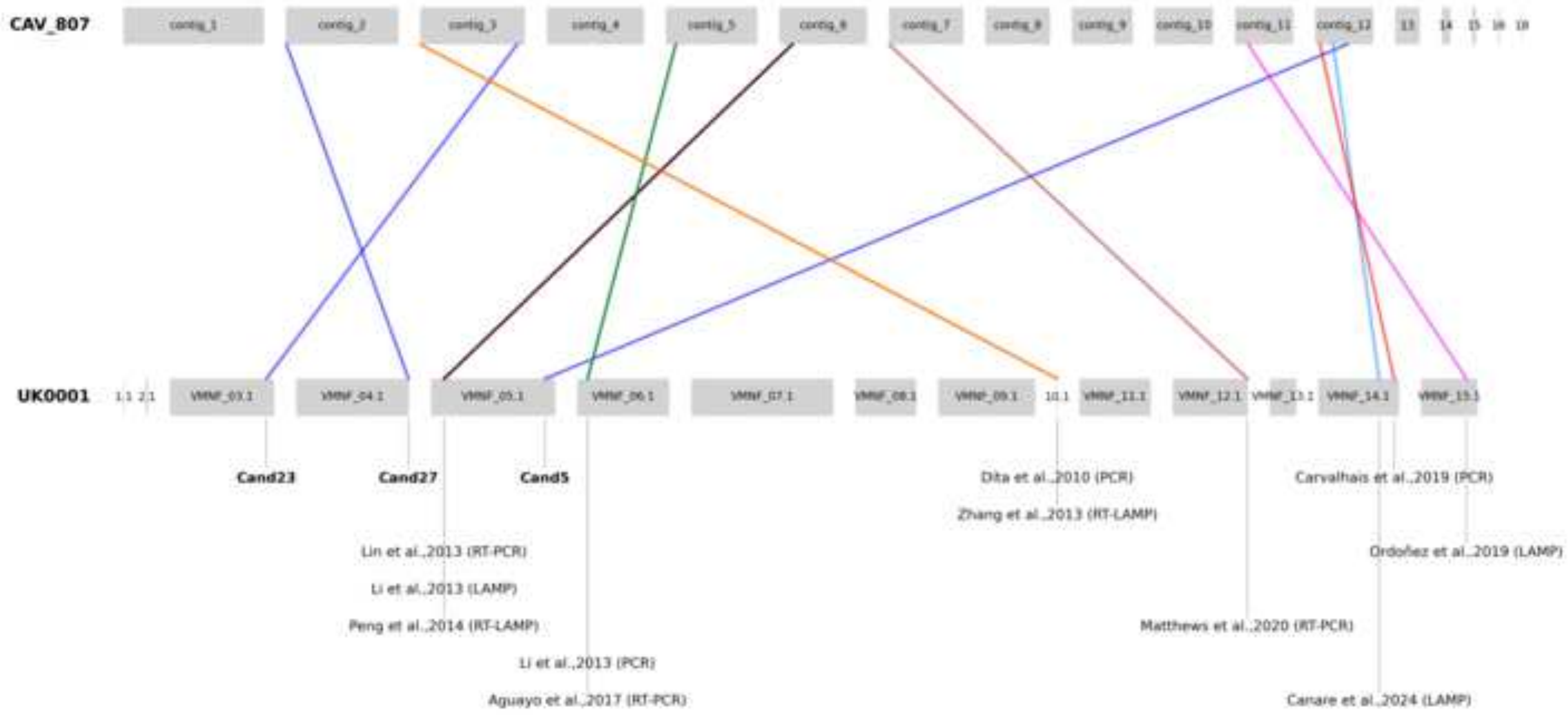

Figure3.Position and direction of the LAMP primers on the Cand-23 region.

Click here to access/download;Figure;Fig3.tiff

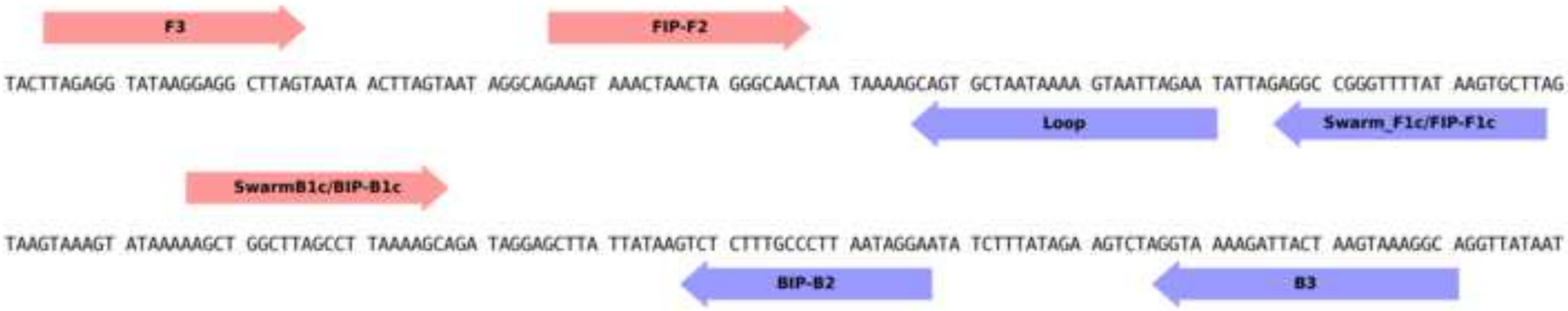

Figure4. Sensitivity of the Cand-23-LAMP system as assessed in plasmid dilutions (a) and in plasmid-spiked plant tissue extractions (b)

Click here to access/download;Figure;Fig4.tiff

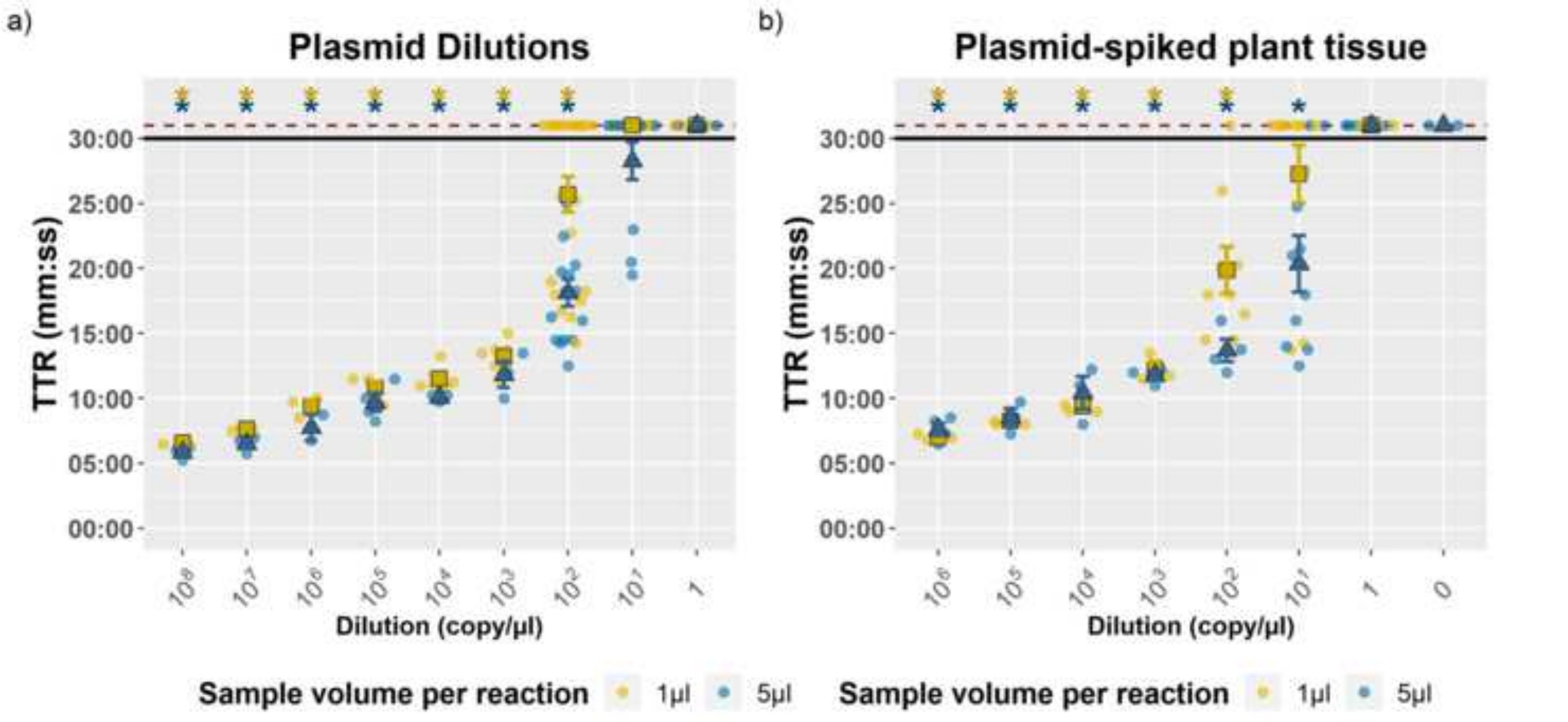

Figure5. Cand-23 in planta result comparison

Click here to access/download;Figure;Fig5.tiff

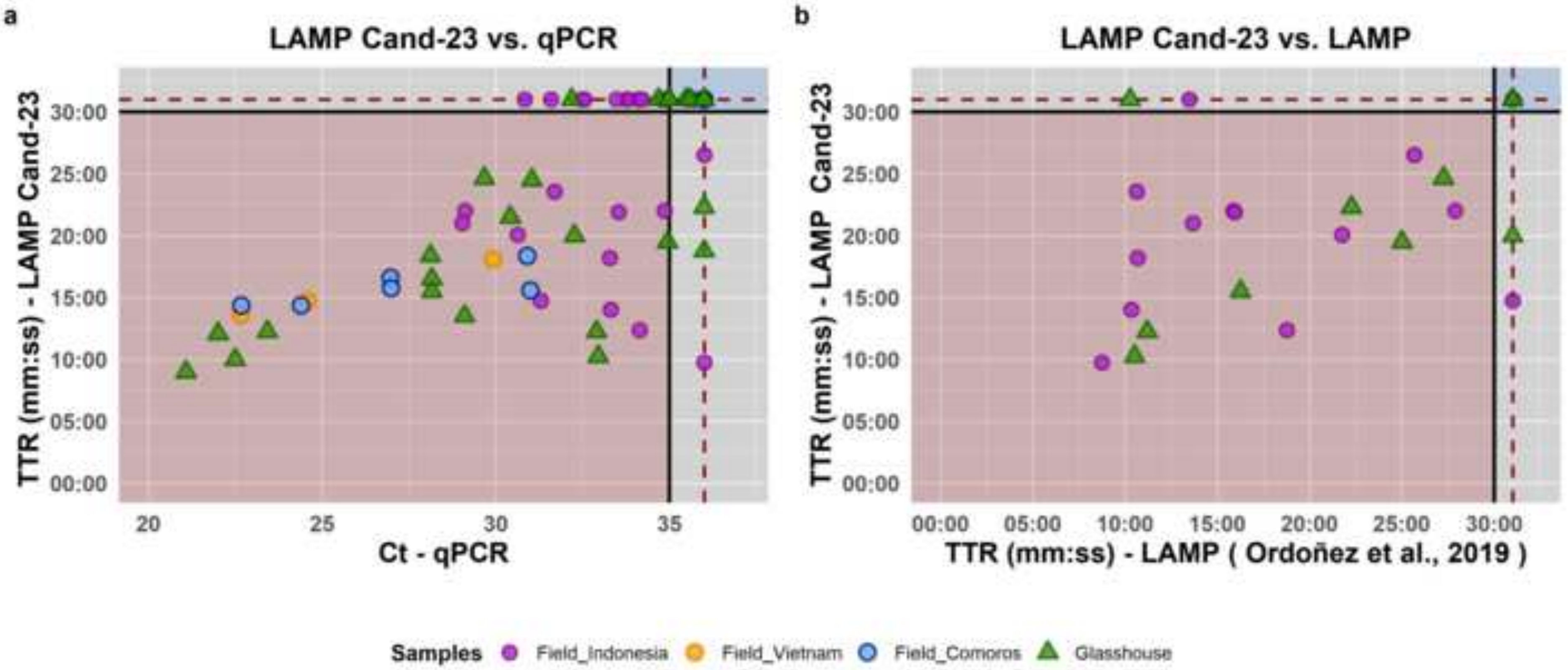

S1_Figure. Plasmid diagram

Click here to access/download
Supporting Information
S1_Fig_Plasmid_diagram.pptx

S2 Figure. Primer optimisation recipe comparison test

Click here to access/download
Supporting Information
S2_Fig_REVISED.pptx

S3 Figure. Sensitivity results

Click here to access/download
Supporting Information
S3_Fig_REVISED.pptx

S1 Table.Detailed information on Genomic Data

Click here to access/download
Supporting Information
S1_Table_REVISED.xlsx

S2_Table. Detailed parameters of the KEC candidates and their derived primers

Click here to access/download
Supporting Information
S2_Table_Candidates_KEC_parameters_PCR_Primers.xlsx

S3 Table. Detailed information on the DNAs

Click here to access/download
Supporting Information
S3_Table_REVISED.xlsx

S4_Table. Detailed informations on the LAMP candidates as compared with LAMP and PCR/qPCR assays already published

Click here to access/download
Supporting Information
S4_Table_Candidate_summary.xlsx

S5 Table. Detailed specificity results of LAMP

Click here to access/download
Supporting Information
S5_Table_REVISED.xlsx

S6_Table. Detailed Sensitivity Results of our LAMP candidates compared with qPCRs and LAMP references.

Click here to access/download
Supporting Information
S6_Table_Sensitivity_detailed_results.xlsx

S7 Table. Detailed TR4 diagnostic results of LAMP.

Click here to access/download
Supporting Information
S7_Table_REVISED.xlsx

Comparative genomics-based development of a LAMP assay for rapid and reliable in-field detection of *Fusarium oxysporum* f. sp. *cubense* tropical race 4

M. Arrieta*[1,2], D. Mostert[4], S. Ravel[1,2], S. Rozsasi[3,4], V. Maillot-Lebon[3], A. Sutanto[5], C. Hermanto[5], Mouzdalifa Mmadi[6], Abdou Azali Hamza[6], N.A. Lubin[1,2], B. Fenelon[3], H. Adreit[1,2], S. Fabre[1,2], B. Coetzee[4], J. Jansen van Vuuren[4], S. Le Roux[4], A. Viljoen[4], Y. Chilin-Charles[2,7], J. Carlier[1,2], C. Gianinazzi[8], E. Wicker*[1,2] and I. Robène*[3,4]

[1] CIRAD, UMR PHIM, F-34398 Montpellier, France.

[2] PHIM, Plant Health Institute, Univ Montpellier, INRAe, CIRAD, Institut Agro, Montpellier, France

[3] CIRAD, UMR PVBMT, F-97410 Saint-Pierre, Réunion Island, France.

[4] Department of Plant Pathology, Stellenbosch University, Stellenbosch 7602, South Africa.

[5] BRIN, Jl. Raya Bogor No.970, Nanggewer Mekar, Kec. Cibinong, Kabupaten Bogor, Jawa Barat 16915, Indonesia

[6] Institut National de Recherche pour l'Agriculture, la Pêche et l'Environnement (INRAPE), BP 289, Moroni, Union des Comores

[7] CIRAD, UMR PHIM, F-97130 Capesterre-Belle-Eau, Guadeloupe, France.

[8] QUALIPLANTE SAS, F-34880 Laverune, France.

***Corresponding authors**

E-mail :ondance : mikarriet@gmail.com, emmanuel.wicker@cirad.fr, isabelle.robene@cirad.fr

ORCID ID:

- Mikel Arrieta: https://orcid.org/0000-0001-7209-0672
- Diane Mostert: https://orcid.org/ 0000-0002-3382-8555
- Sebastien Ravel: https://orcid.org/ 0000-0001-6663-782X

Formatted: Font: Bold, No underline, Font color: Auto
Formatted: No underline, Font color: Auto
Formatted: Font: Bold, No underline, Font color: Auto
Formatted: No underline, Font color: Auto
Formatted: Font: Bold, No underline, Font color: Auto

- Samuel Rozsasi: https://orcid.org/ 0009-0008-7182-3764
- Beatrix Coetzee: https://orcid.org/ 0000-0001-5374-8918
- Altus Viljoen: https://orcid.org/ 0000-0001-7146-0491
- Yolande Chilin-Charles: https://orcid.org/ 0009-0009-1584-5149
- Jean Carlier: https://orcid.org/0000-0002-6967-1852
- Emmanuel Wicker: https://orcid.org/0000-0003-0927-7404
- Isabelle RobeneRobène: https://orcid.org/0000-0003-2012-4652

Short title: A genome-driven Foc-TR4-specific Assay for Field Diagnostic

Formatted: No underline, Font color: Auto

Formatted: Font color: Auto

Formatted: Font: Bold, No underline, Font color: Auto

Formatted: No underline, Font color: Auto

Formatted: Font: Bold, No underline, Font color: Auto

Formatted: Font: Not Bold

Formatted: Font: Not Bold

Formatted: Font: 11 pt

Formatted: Font: 11 pt

Abstract

Fusarium wilt, caused by the soilborne fungus *Fusarium oxysporum* f. sp. *cubense* (Foc) Tropical race 4 (TR4) is currently devastating Cavendish banana production worldwide. The availability of accurate and rapid field-deployable detection tools is vital for effective disease prevention and containment practices, as no effective treatment is currently available. Design of reliable molecular detection tools, however, is challenged by genetic similarity between non-pathogenic and target Foc strains that are present in symptomatic plant material. The high genetic diversity present among Foc isolates further complicates the identification of unique genomic regions suitable for diagnosis.~~tic~~ Current molecular assays are also still mostly confined to laboratory settings and relies on expensive equipment that needs skilled operators to perform. In the present study, a simple loop-mediated isothermal amplification (LAMP) detection assay was developed for direct in-field detection. A comprehensive database of Foc genomes including 148 TR4 genomes and 146 non-target genomes was used, considering the wide diversity within Foc. These genomic sequences were exploited in a comparative genomics *in silico* pipeline using the KEC method (K-mer elimination by cross-reference), combined with filtering steps using BLASTn, and Illumina read alignments. The specificity of the LAMP primers was validated using a diverse collection of 161 isolates, which included Foc-TR4 isolates, Foc VCGs, *F. oxysporum* spp. endophytes, other *Fusarium* spp., and genera which are commonly isolated from banana vascular tissue. The limit of detection reached 10^2 copies~~.~~ µL^{-1} of plasmid DNA, and the target pathogen was successfully identified both from artificially inoculated and naturally infested material in contrasted environments, indicating the LAMP assay to be a robust and reliable tool fully suitable for field diagnosis.

Keywords: Tropical race 4, diagnosis, LAMP, fungal plant pathogens, *Fusarium oxysporum* f. sp. *cubense*, Fusarium wilt of banana

Introduction

Fusarium wilt of banana is a soilborne vascular disease that threatens global banana production, and is considered one of the most destructive plant diseases worldwide [1-3]. The causaling agent is *Fusarium oxysporum* f. sp. *cubense* (Foc), a highly diverse fungal species which different strains have caused two major epidemics in the last century. Foc-Race 1 caused destruction of the most widely cultivated variety during the 20th century, Gros Michel [4, 5]. This first Fusarium wilt epidemic could only be brought under control after the 1950s by the deployment of a resistant cultivar called Cavendish, which currently represents the 99% of worldwide banana exports and more than 40% of global banana production [6-9] [. The "return of the banana Fusarium wilt calamity" occurred in the early 1990s with outbreaks in Indonesia, Malaysia, and Taiwan, of a new Foc race called "tropical race 4" (TR4) [2, 10]. This race affects Cavendish banana cultivars, as well as the other cultivars susceptible to Race 1 [2, 11, 12][2,11,12]. The resilience of Foc due to the production of survival structures called chlamydospores, and the acceleration of global trade during the last decades favoured the spread of the pathogen. Within the last two decades it has rapidly spread from its origin in Asia to major banana growing regions in the Middle East, Africa and Latin America [2, 3, 13-15] [2,3,13–15].

Millions of people worldwide rely on banana both as a staple food and as a source of income [16][16], and hence solutions for the Foc-TR4 epidemic are crucial to avoid consequences of another wipeout of the currently preferred cultivar. As a soilborne pathogen, Foc can be transmitted through irrigation, watercourses and floodwater, as well as field tools, vehicles, machinery, contaminated plants and soil. It can also survive in the soil for decades [4, 11, 17][4,11,17]. Once Foc is established within a field, there is no effective control methods to ensure its complete eradication [11][11].

Early detection and containment are crucial in response to Foc-TR4 epidemics in the absence of disease management strategies [11]~~[11]~~. Molecular detection tools are the preferred method for disease detection and there is an abundance of assays published including PCR [18, 19]~~[18,19]~~, quantitative qPCR [20, 21]~~[20,21]~~, and loop-mediated isothermal amplification (LAMP), as reviewed in Roberts *et al.* [22]~~[22]~~. The design of a reliable molecular detection assay for Foc-TR4 has faced several challenges. First molecular assays targeted ~~One is the frequent use of~~ ~~a~~ single sequences or single nucleotide polymorphism (SNPs) in core genes (e.g. the intergenic spacer region (IGS)), whereas these genes are conserved ~~which are conserved~~ among both target and non-target Fusaria [23]~~[23]~~. Consequently, endophytic *F. oxysporum* with homology in these regions, are often identified as false positives in molecular assays designed in these core gene regions [24]~~[24]~~. Another challenge consists of the specific identification of the Foc-TR4 lineage among the different races, VCGs, and Foc genetic clades. The banana Fusarium Wilt agent, Foc, is indeed a polyphyletic and diverse organism, composed of three genetic clades (A, B, C *sensu* O'Donnell et al., 2004 [25] ~~[25]~~), corresponding respectively to the *Fusarium oxysporum* Species Complex (FOSC) Clades 1, 2, and 3 [26]~~[26]~~, eight subclusters (Foc-SC), more than 24 vegetative-compatibility groups (VCGs) [27]~~[27]~~ and four races based on the host specificity.~~.~~

Foc TR4 isolates can cause disease to Cavendish banana in tropical conditions [28]~~[28]~~. According to various authors, it is a synonym of the VCG 01213/16 [29, 30][~~29,30]~~, which has been controversially reclassified as *Fusarium odoratissimum* [31] ~~[31]~~, and~~which~~ which can also include closely related VCG 0121 [2]~~[2]~~. Race 4 is further divided into Subtropical Race 4 (STR4), which infects Cavendish only in subtropical conditions after stress predisposition [32]~~[32]~~. Furthermore, Race 4 isolates can also affect varieties affected by Race 1 and 2, which makes the race concept confounding and challenges development of accurate molecular diagnostics [22]~~[22]~~.

Formatted: Font: Not Italic

Current Foc-TR4 molecular assays are also mostly confined to laboratory settings and rely on expensive molecular equipment and requires skilled operators to perform these assays. There is a need for a TR4 diagnostic system to be Point-of-care (POC), implying that the testing and assay be carried directly in the field and with rapid and simplified protocols. Loop-mediated isothermal amplification (LAMP) assays enable a rapid, simple, sensitive and robust detection [33, 34]~~[33,34]~~. LAMP machines are usually portable, and the enzyme used is often less sensitive to background inhibitors, which makes it more compatible with simplified DNA extraction methods that can be done directly from sampled plant material. Among the different LAMP assays developed for detecting FocTR4, some are unable to distinguish between TR4 and STR4 [19, 35]~~ (Li et al., 2013; Peng et al., 2014)~~ or generate false positives [36]~~(Zhang et al., 2013)~~. Conversely, the LAMP assay developed by Ordóñez et al.~~ (2019)~~[37] appears to be a reliable tool for detecting FocTR4.

In this study, a novel Foc-TR4 LAMP assay was designed with the aim to be field deployable. We considered a large and comprehensive genomic and genetic collection of fungal strains to identify specific markers, design adequate LAMP primers, as well as to address the limitations and specificity of the existing LAMP systems. The LAMP assay was designed with a simple and cost-effective DNA extraction protocol which can be conducted by unskilled operators. We compared its diagnostic performance with existing molecular tools including the Ordóñez LAMP assay [37]~~developed by Ordóñez et al. (2019)~~. The assay successfully detected Foc-TR4 both from artificially inoculated and naturally infected banana material and could be conducted directly in field settings. The proposed system provides a useful tool useful for early Foc-TR4 detection during surveillance and containment.

Materials and methods

Genomic resources

The genomic resources used in this study included a total of 294 genomes (summarized in Table 1 and detailed in S1 Table). Among these genomes are those available in an in-house genomic database shared between CIRAD and Stellenbosch University including 118 target Foc-TR4 genomes, 47 genomes from other Foc VCGs and 2 endophytic *F. oxysporum* isolates. Additionally, 127 genomes were downloaded from the NCBI database (National Center for Biotechnology Information, Maryland, United States) or the NGDC (National Genomics Data Centre, Beijing, China). These included 31 target Foc-TR4 genomes, 16 non-target Foc genomes, 51 genomes of various other *formae speciales* of *Fusarium oxysporum*, 29 other genomes from *Fusarium* genus, and other microbial genera commonly associated with bananas. These genomic resources were available in three different formats: Illumina short read sequencing data (n=172), genome assemblies that had been sequenced using the Oxford Nanopore Technology (ONT) (n=21), and genome assemblies that were publicly available (n=101).

Field Code Changed

Formatted: Font: Not Bold

Table 1: Summary of the genomic resources and DNA from microbial isolates used in this study.

Species	Clade / Foc-SC[a]	VCG	Race	Genomic Resources				TOTAL DNA Samples
				Illumina Reads	ONT Assembly	Public Assembly	TOTAL Genomes	
F. oxysporum f. sp. cubense	A/1	0120	STR4	4	1	1	**6**	**6**
F. oxysporum f. sp. cubense	A/1	0120/15	STR4	5	0	0	**5**	**4**
F. oxysporum f. sp. cubense	A/1	01210	STR4	7	0	0	**7**	**1**
F. oxysporum f. sp. cubense	A/1	01219	STR4	1	0	0	**1**	**1**
F. oxysporum f. sp. cubense	A/1	0122	STR4	1	1	0	**2**	**2**
F. oxysporum f. sp. cubense	A/1	0129/11	STR4	1	0	0	**1**	**2**
F. oxysporum f. sp. cubense	A/1	01215	STR4	1	0	0	**1**	**1**
F. oxysporum f. sp. cubense	A/1	01227		0	0	0	**0**	**1**
F. oxysporum f. sp. cubense	A/1	0126	STR4	1	0	0	**1**	**1**
F. oxysporum f. sp. cubense	A/1	0129	STR4	1	0	0	**1**	**1**
F. oxysporum f. sp. cubense	A/1	SMV 1	UNK	0	0	0	**0**	**1**
F. oxysporum f. sp. cubense	A/1	SMV 2	UNK	0	0	0	**0**	**1**
F. oxysporum f. sp. cubense	A/2	0121	Race 4	1	5	0	**6**	**9**
F. oxysporum f. sp. cubense	A/2	01213/16	**TR4**	126	14	8	**148**	**46**
F. oxysporum f. sp. cubense	A/2	SMV 3	unk	0	0	0	**0**	**1**
F. oxysporum f. sp. cubense	B/4	01212	Race 1	1	0	0	**1**	**1**
F. oxysporum f. sp. cubense	B/4	01220	Race 1	2	0	1	**3**	**1**
F. oxysporum f. sp. cubense	B/4	01222	Race 1	1	0	0	**1**	**1**
F. oxysporum f. sp. cubense	B/4	0124	Race 1	3	0	1	**4**	**3**
F. oxysporum f. sp. cubense	B/4	0124/25	Race 1	0	0	0	**0**	**1**
F. oxysporum f. sp. cubense	B/4	0124/5	Race 1	1	0	0	**1**	**2**
F. oxysporum f. sp. cubense	B/4	0124/5/8	Race 1	1	0	0	**1**	**1**
F. oxysporum f. sp. cubense	B/4	0125	Race 1	1	0	1	**2**	**1**
F. oxysporum f. sp. cubense	B/4	0128	Race 1	1	0	0	**1**	**3**
F. oxysporum f. sp. cubense	B/4	UNK	Race 1	0	0	0	**0**	**3**
F. oxysporum f. sp. cubense	B/4	01228	UNK	0	0	0	**0**	**1**
F. oxysporum f. sp. cubense	B/4	01229	UNK	0	0	0	**0**	**1**
F. oxysporum f. sp. cubense	B/4	01232	UNK	0	0	0	**0**	**1**
F. oxysporum f. sp. cubense	B/4	01233	UNK	0	0	0	**0**	**1**
F. oxysporum f. sp. cubense	B/4	01236	UNK	0	0	0	**0**	**1**
F. oxysporum f. sp. cubense	B/4	0124/22	UNK	1	0	0	**1**	**0**
F. oxysporum f. sp. cubense	B/4	0128/20	UNK	0	0	0	**0**	**1**
F. oxysporum f. sp. cubense	B/4	SMV 4	UNK	0	0	0	**0**	**1**
F. oxysporum f. sp. cubense	B/4	SMV 5	UNK	0	0	0	**0**	**1**
F. oxysporum f. sp. cubense	B/4	SMV 6	UNK	0	0	0	**0**	**1**
F. oxysporum f. sp. cubense	B/5	01214	UNK	1	0	0	**1**	**5**
F. oxysporum f. sp. cubense	B/5	01231	UNK	0	0	0	**0**	**1**

F. oxysporum f. sp. cubense	B/6	01217	Race 1/2	1	0	0	**1**	**0**
F. oxysporum f. sp. cubense	B/6	01223	UNK	1	0	0	**1**	**0**
F. oxysporum f. sp. cubense	B/6	01224	UNK	1	0	0	**1**	**1**
F. oxysporum f. sp. cubense	B/6	0123	Race 1/2	1	0	0	**1**	**2**
F. oxysporum f. sp. cubense	B/7	01218	UNK	1	0	0	**1**	**1**
F. oxysporum f. sp. cubense	B/7	01234	UNK	0	0	0	**0**	**1**
F. oxysporum f. sp. cubense	B/7	01235	UNK	0	0	0	**0**	**1**
F. oxysporum f. sp. cubense	B/7	SMV 10	UNK	0	0	0	**0**	**1**
F. oxysporum f. sp. cubense	B/8	01221	Race 1/2	1	0	0	**1**	**1**
F. oxysporum f. sp. cubense	B/8	01230	UNK	0	0	0	**0**	**1**
F. oxysporum f. sp. cubense	C/3	01226	UNK	0	0	0	**0**	**3**
F. oxysporum f. sp. cubense	UNK	UNK	Race 1	1	0	3	**4**	**5**
F. oxysporum f. sp. cubense	UNK	UNK	STR4	12	0	0	**12**	**0**
F. oxysporum f. sp. cubense	UNK	UNK	UNK	0	0	5	**5**	**0**
F. oxysporum			Banana endophyte	2	0	0	**2**	**3**
F.oxysporum, other formae speciales [b]				0	**29**	**30**	**12**	0
F. oxysporum, others [c]				0	0	22	**22**	**12**
Fusarium, other species [d]				0	29	29	3	0
Nectria sp. FW16.1				0	0	1	**1**	**0**
Pseudocercospora fijiensis				0	0	0	**0**	**3**
Ralstonia solanacearum				0	0	0	**0**	**1**
**Total Target (TR4)**				**126**	**14**	**8**	**148**	**46**
**Total Non Target**				**46**	**7**	**93**	**146**	**115**
**Total**				**172**	**21**	**101**	**294**	**161**

The data type used is indicated as Illumina short read genomes, ONT-based assemblies, or Public assemblies (from publicly available genomes). All F. *oxysporum* f. sp. other than *cubense* were not pathogenic on banana.

[a]The Clade and VCG classification is based on Mostert *et al*., 2022 [27][27]. SMV: Single-Member VCG. UNK: Unknown.

[b] The other *formae speciales* of *Fusarium oxysporum* tested were: *canariensis, cepae, ciceris, conglutinans, cucumerinum, cyclamini, dianthi, fragariae, koae, lini, lycopersici, radicis-lycopersici, radicis-vanillae, rapae, strigae, vanillae, vasinfectum, spinaciae*

[c] *F.oxysporum* strains which were not assigned to a specific *forma specialis*.

[d] The other *Fusarium* species tested included *F.agapanthi, F. avenaceum, F. falciforme, F. fujikuroi, F. graminearum, F. haematococcum, F. langsethiae, F. louisianense, F. metavorans, F. neocosmosporiellum, F. pseudoanthophilum, F. pseudonygamai, F. ramigenum, F. redolens, F. solani, F. udum, F. vanettenii, F. venenatum, F. verticillioides*

Identification of a target genomic region

The K-mer elimination by cross-reference (KEC) method v.1.0 [38]~~[35]~~ (Step 1 in Fig 1) was used to identify target specific candidate sequences. This approach is fed by genome assemblies that are user-defined as target or non-target genomes. In addition to the 21 ONT-based in-house assemblies [39, 40]~~[36,37]~~, and the 101 publicly available genomes, all the Illumina short read genomes (172) were assembled to maximise the number of input genomes. The assembler ABySS **v.2** [41] ~~[38]~~ software was used with an automated ~~optimized~~optimised best K-mer selection through the *ABYSS_launch.py* workflow, written by Florian Charriat (https://github.com/FlorianCHA/script_paper). Importantly, these assemblies were repeat masked using RepeatMasker (http://repeatmasker.org/).

Fig 1. Pipeline followed to find TR4-specific sequences. Step 1 assemblies of target and not-target genomes were used for the KEC (K-mer elimination by cross-reference) method described by Beran et al.~~, (2021~~)[38] to obtain an initial list of target specific candidate sequences. These sequences were queried (**Step 2**) in an internal BLAST to double-check the presence/absence on non-target genomes. The remaining candidate sequences were filtered in two ways. Firstly, based on the results obtained when a query was done with BLAST on the NCBI online database (**Step 3**), whilst the second (**Step 4**) aligned Illumina reads on the candidate sequences with RattleSNP software ~~(~~(https://github.com/sravel/RattleSNP)~~Ravel)~~, and computed coverage of query reads on sequence tested. Figure created with Miro™.

The ONT-based inhouse assembly of CAV807 ~~(~~([39]~~Bache, 2022 [36];~~ available upon request) was chosen as the main target reference genome (master) on the KEC method, using the "pool" feature with the rest of target genomes. A combination of parameters was tested to do KEC computations (test details included on S2 Table) with a minimum output sequence size range from 60 to 500 bp, K-mer size ranges from 13 to 19, different input populations, and including or excluding reverse complement sequences, all of which produced different output candidate sequence lists. All these

Formatted: Font: 18 pt

Formatted: Font: Not Bold

Formatted: Font: Not Bold

Field Code Changed

sequences were then merged on a single file which was used as reference sequences on the following steps (Steps 2-4 in Fig 1).

To verify that the output sequences were present on the target genomes while absent from non-target genomes, an internal BLASTN query (Step 2, Fig 1) was done for all the candidate sequences on all the available genomes; candidates that had high BLAST hits on non-targeted genomes (more than 90% identity of full queried sequence) were discarded. The best sequence candidates were also queried against fungal genomes (Whole-Genome shotgun contigs database version for Fungi, taxid 4751) on the NCBI database (step 3, Fig 1) (https://blast.ncbi.nlm.nih.gov/Blast.cgi). The candidate sequences were regarded as good targets for primer design if (i) they got a hit on all the available TR4 genomes on NCBI (named "*Fusarium oxysporum* f. sp. *cubense* TR4", or "*F.odoratissimum*"), and (ii) they got the minimum hit on non-TR4 genomes. As a reference, internal BLASTN and NCBI database queries were performed in parallel using the sequence targeted in the Ordóñez LAMP assay [37]~~by Ordóñez et al. (2019)~~, which was the latest published LAMP TR4-diagnostic region at the time of this study.

Additionally, the genomic Illumina reads of all genomes available in this format were mapped (Step 4 Fig 1 on the candidate sequences used as references, using the workflow RattleSNP (https://github.com/sravel/RattleSNP). Mapping 150 bp reads on such a short reference sequence may result in misalignments. To avoid this, the candidate sequences were extended 1000 bp up and downstream using the unmasked version of the ONT-based CAV807 assembly, before the alignment step, The sequences which had the highest percentages of coverage among all target Illumina genomes (TR4), and lowest coverage on non-targeted genomes, were selected.

PCR primer design and initial screening of selected candidate sequences

Initial screening of best performing candidate sequences was done either in PCR or Real-time quantitative PCR (qPCR). Primers were designed using Primer 3 (https://primer3.ut.ee/), and assays were performed on a bCUBE machine (Hyris Ltd, London, United Kingdom), adjusting the amplification protocol for each case primer system. For PCRs, the Promega GoTaq® G2 MasterMix (Promega, Wisconsin, United States) kit was used, and PCR products were assessed on a 1.5% Agarose Gel~~ (data not shown)~~, whilst for qPCR the SYBR™ Select Master Mix qPCR (Thermo Fischer, Waltham, Massachusetts, United States) was used.

LAMP primer design and assay conditions

LAMP primers were designed using the Primer explorer V5 software (primerexplorer.jp/lampv5e/index.html). When software did not yield Loop primers by default settings, design was done manually. A similar approach was used for the design of Swarm primers for ~~which design~~which design option is not included in the software. LAMP reactions were performed on the portable Genie II machine (Optigene, Blatchford, United Kingdom), in a total reaction volume of 25 µL, containing 15 µL ISO-DR004 Isothermal Mastermix (OptiGene, Horsham, UK), 2.5 µL of pre-mixed Primer Mix, (resulting in final concentrations of 0.2 µM of each F3 and B3 primers, 0.8 µM of each FIP and BIP primers, 0.4 µM of each loop primer), and 5µL of template DNA. Note that within the specificity tests, 1 µL of template DNA was used instead of 5µL. LAMP reactions were run at 65°C for 30 min, followed by a 6 min annealing step with temperatures varying from 98°C to 80°C at a speed of 0.05°C~~/~~ per second. Amplification curves associated with a “Time to Result value” (TTR values) and annealing temperature (*Ta*) peaks were generated and recorded during the LAMP reaction. A sample was considered positive if (i) the average TTR of the replicates of a sample was below the established minimum TTR threshold (30:00), (ii) if the average *Ta* peak was ± 1 °C from the reference Ta (relative to each LAMP system), and (iii) if the fluorescence of the Ta peak reached a minimum threshold value of 1500. By convention, a value of 31:00 (just above the time limit) was assigned to a negative result (TTR=00:00) for the calculations and graphical

representations (as in Robène et al., 2023)(as in [42]). In the case of sensitivity tests, the limit of detection (LOD) was considered as the minimum dilution of DNA at which the mean of the replicates could produce a positive result at a 95% of confidence level.

Reference assays

The Ordóñez LAMP assay [37] described in [37]Ordóñez et al., 2019 was used with the LAMP conditions described above and the program described in the publication. Additionally Two TR4-specific qPCRs systems were considered as references: (i) The hydrolysis probe-based real-time assay developed by AguayoAguayo *et al.* 2017[20], which is the current reference method in France by the French Agency for Food, Environmental and Occupational Health and Safety, ANSES [43][41], along with the Promega GoTaq™ Probe qPCR Master Mix (Promega, Wisconsin, United States); and (ii) the SYBR™-based real-time assay [21]developed by Matthews *et al.* 2020 along with the SYBR™ Select Master Mix qPCR (Thermo Fischer, Waltham, Massachusetts, United States). A sample was considered positive when the mean Threshold Cycle (Ct) over all replicates was below a threshold of 35 cycles.

Specificity testing

A set of 161 DNA samples was used for specificity testing in the present study; including 46 target Foc-TR4 isolates and 82 non-target Foc isolates representing intraspecies diversity (S3 Table). In addition, other non-target isolates included *F. oxysporum* endophytes and other *F. oxysporum formae speciales* (n=25), other *Fusarium* spp. (n=5), and pathogens of banana (3 isolates of *Pseudocercospora fijiensis,* causing the black Sigatoka; and one isolate of *Ralstonia solanacearum,* causing the Moko disease). Moko strain was grown onto tetrazolium chloride medium (TZC) at 28 °C [44][42]. Bacterial cultures grown overnight were used for DNA extraction using a DNeasy Blood and tissues kit (Qiagen, Courtaboeuf, France). Fungal cultures were grown on potato dextrose agar (PDA) for seven days at 25°C, then transferred to a liquid medium (Nitrate medium; Yeast Nitrogen

Field Code Changed

Formatted: Font: Not Bold

Base: 1.7 g .L^{-1}, Sucrose : 30 g. L^{-1}, KNO_3: 10.11 g. L^{-1}; M. de Saint & M. Rep, personal comm. 2020) in an Erlenmeyer flask for five to seven days. The mycelia were filtered through sterile cheesecloth by rinsing with sterile water. Mycelia of each isolate was then groundinded with mortar and pestle in liquid nitrogen prior to DNA extractions performed following an in-house protocol (S1 Methods). DNA quantity and quality was assessed on a Nanodrop™ spectrophotometer (Thermo Fischer, Waltham, Massachusetts, United States). DNA concentration was adjusted to 1-2 ng.µLµl^{-1} before it was tested with optimizedoptimised LAMP assays. The rest of extractions were performed in Stellenbosch University following protocols detailed ealierin[45, 46] [43,44].

Sensitivity testing

Different DNA samples were prepared for sensitivity tests including pure plasmid DNA samples, plasmid spiked plant material, as well as plant material artificially inoculated with conidial suspensions.

Sensitivity based on plasmid dilution series

A plasmid containing the selected DNA sequence targeted by the LAMP primers was ordered from Eurofins (Eurofins, Luxembourg) (S1 Fig). This was initially resuspended in TE buffer (Sigma-Aldrich, Massachusetts, United States) as recommended by the manufacturer, and dilution series were prepared in water (10^8 copies/ µL^{-1} to 10^0 copies/µL µL^{-1}).

Sensitivity based on plasmid-spiked plant material

Plasmid-spiked plant tissue samples were prepared by first grinding healthy plant tissue from corm and pseudostem in liquid nitrogen, after which approximately 100 mg of healthy tissue collected in Eppendorf tubes. To spike the samples, 10 µL of each plasmid dilution (prepared as in 2.7.1) were then mixed with the ground material. DNA was then extracted with the DNeasy Plant Kit protocol as per manufacturer's instructions (Qiagen, Hilden, Germany), but elutded in a final volume of 100 µL,

Formatted: Subscript

Formatted: Font: Not Bold

Formatted: Font: Not Bold

Field Code Changed

Formatted: Superscript

which was used to calculate the final concentration of plasmid in each sample (from 10^5 conidia µL^{-1}, downto 0 conidia µL^{-1}).

Sensitivity based on plant material inoculated with conidial suspensions

The Foc-TR4 isolate MAY0001 was used to prepare an initial conidial suspension. Conidial concentrations (10^7 conidia mL^{-1} downto 10^0 conidia mL^{-1}) were adjusted with haemocytometer (KOVA, California, United States) visualised under a light microscope. Each conidial dilution was mixed with about 100 mg of ground healthy plant tissue from corm and pseudostem and then centrifuged at 14 rpm for 5 mins. DNA extraction was performed on the pellet using the DNeasy Plant Kit (Qiagen, Hilden, Germany). Extractions were done in duplicate for each prepared concentration (from 10^5 conidia µL^{-1}, to 0 conidia µL^{-1}).

Detection from artificially inoculated material

Conidial suspensions of MAY0001 were prepared as described ~~in 2.7.3~~above. Three-month old tissue culture-derived banana plants which were previously hardened in the CIRAD-PHIM glasshouse Montpellier, France. Infections were conducted by pouring 20 ml conidial suspensions at a concentration of 1x10^6 conidia mL^{-1} around the base of plant. After ten weeks of incubation (30°C day/ 26°C night, 90% RH, 2h of additional lighting (300W m^{-2}) to ensure 12h daylight during winter), infection was assessed by cross-sections of the corm. Where infection was evident including reddish brown discoloration of the vascular tissues and rhizomes, tissue was collected and frozen at -20°C. Tissue was then ground to a powder in liquid nitrogen and transferred to Eppendorf™ tubes (Eppendorf, Hamburg, Germany). About 100 mg of plant material from each sampled plant were used for DNA extraction with the DNeasy Plant Kit following the instructions of the manufacturer (Qiagen, Hilden, Germany). DNA quantity was measured with Nanodrop. The sensitiv~~bil~~ity of the

optimised LAMP assay to detect Foc-TR4 DNA was compared to the mentioned published assays ~~(2.5)~~.

In-field deployment and detection from naturally infested plant material

The optimised LAMP assay was deployed on site during a field survey in Java, Indonesia, organized in collaboration with the local institute BRIN (National Research and Innovation Agency). The survey was conducted in the Cianjur regency, at four different plots in the Cikalongkulon and Mande districts where Foc-TR4 were previously reported. Thirty-two plants of different banana cultivars, including both Cavendish and non-Cavendish cultivars of various genomic formulas (detailed in S7 Table), were sampled, making a total of 70 samples collected from fruit, core of pseudostem, outer pseudostem, corm, or peduncles, ~~from various cultivars~~ which were analysed both with the LAMP assay ~~optimized~~optimised in this study, with published LAMP [37]~~as described by Ordoñez *et al.* (2019)~~ and the Matthews qPCR [21]~~described by Matthews *et al.*, 2020~~. Additionally, eight pseudostem samples collected from Comoros (Grande Comore) and Vietnam respectively that were sent to Stellenbosch University for VCG characterisation, were also analysed.

A rapid DNA extraction method was modified from ~~Robène *et al.* (2023)~~Robène *et al* [42], for extraction from collected pseudostem tissues: About 250 mg of pseudostem tissue was transferred to Bioreba extraction and.an volume of 1 ml 0.5 M NaOH including 2% PVP added, which was then ground with hand grinder (Bioreba, Reinach, Switzerland). After grinding, 5 µL of the resulting homogenate liquid was then collected and diluted into 195 µL 100 mM Tris (pH 8.0). This homogenate was immediately analysed in LAMP assays. In parallel, the Mathews ~~*et al.* 2020~~ qPCR [21] was performed on the duplicate samples extracted with a magnetic bead extraction kit according to the supplier's protocol (Sera-Mag Select, cytiva, Marlborough, USA)

Formatted: Font: Italic

Statistical analysis for the test comparisons

The results of the LAMP assays on plant material were compared with each other and with the qPCR results by the Cohen's kappa index and the McNemar's test [47] ~~[45]~~. Cohen's kappa index was interpreted as in McHugh 2012[48] ~~[46]~~ to evaluate the agreement between techniques: Kappa ≤ 0 corresponds to no agreement, 0.01–0.20 is "none to slight", 0.21–0.40 is "fair", 0.41– 0.60 is "moderate", 0.61–0.80 is "substantial", and 0.81–1.00 is "near-perfect". Additionally, different diagnostic parameters were calculated, such as the Sensitivity, Specificity, Positive Predictive Value (PPV), Negative Predictive Value (NPV), and Accuracy as in [49] ~~Cao *et al.*, 2022~~. A McNemar's p-value below 0.05 was taken as evidence to reject the null hypothesis of equal marginal proportions, indicating a statistically significant difference in detection outcomes between the two methods compared. This would reflect an imbalance in discordant results, i.e. a real shift in the numbers of false positives versus false negatives, which in turn underlies any observed differences in diagnostic parameters such as sensitivity and specificity.

Ethics statement

Field samplings were done in Java, Indonesia, as granted by the National Research and Innovation Agency BRIN (Badan Riset dan Inovasi Nasional, https://www.brin.go.id).

Results

In silico identification and validation of candidate target region

Formatted: Font color: Auto, Not Highlight

Formatted: Line spacing: Double

Formatted: Font color: Auto, Not Highlight

Formatted: Font color: Auto, Not Highlight

Formatted: Font: 12 pt, Not Bold

A total of 1170 candidate target sequences were yielded with the KEC method. The internal BLASTN filtering step (by querying the candidate sequences against the same database) (Step 2, Fig 1) was found a mandatory step, as many candidates were detected both in target and non-target genomes (generally phylogenetically close lineages). Duplicated candidate sequences were also removed, selecting 30 candidate sequences to analyse (details in S2 Table). The list was further reduced to 10 candidate sequences when the amount of non-target genomes was increased in the analysis and sequences showing multiple copies over the target regions were discarded. After the *in silico* specificity testing done by BLAST query on NCBI database on Whole Genome Contigs for all Fungi (taxid 4751) (Step 3, Fig 1), three candidates were finally selected including Candidate 5 (709 bp), Candidate 23 (417 bp) and Candidate 27 (515 bp) located in contigs VMNF01000005.1, VMNF01000003.1 and VMNF01000004.1 of UK0001 reference genome respectively (contigs 12, 3, and 2 on the CAV807 reference used) (Fig 2). All three candidates were identified in intergenic regions, with Candidate 5 being close to a putative gene. All appeared to be target-specific, as theirNone of the candidates showed sequence identities to non-target genome sequences did not exceed 90%y above 90% to non-target genome sequences in the internal database (Detailed **information of the DNA isolates used in this study.**

S4 Table). In external NCBI BLAST analysis (Step 3, Fig 1), three candidates showed some hits with non-target taxa, all with sequence identities below 84% and with partial regions of the queried sequence (Details in Detailed **information of the DNA isolates used in this study.**

S4 Table). Candidate 5 had a non-specific hit on an *Ascodesmis sphaerospora* strain, Candidate 23 showed non-specific BLAST hits with *F. oxysporum* Fo10, an Australian endophyte isolated from soil (Constantin et al., 2021), and on *F. fujikuroi*. Candidate 27 had hits with a *F. proliferatum* genome.

Field Code Changed

Formatted: Font: Not Bold

Formatted: Font: Bold

Field Code Changed

Formatted: Font: Not Bold

Field Code Changed

Formatted: Font: Bold

Formatted: Font: Not Bold

Fig 2. Genomic locations of the amplicons of previously published Foc-TR4 diagnostic systems used on the genomes of UK0001 and CAV807. The three amplicons of the three candidates described in the current publication in bold. The chromosome names of the UK0001 have been simplified to fit in the Fig, being originally “VMNF010000...”.

None of the candidate sequences had BLAST-positive hits on the full number of target Foc TR4 Illumina assemblies used, leading us to a further mapping exploration using the RattleSNP workflow (Step 4, Fig 1). This approach finds alignment coverage and depth of Illumina sequence reads, rather than sequence identity on assemblies, which would allow to explore sequences that had been discarded during assembly process. From all 120 TR4 Illumina genomes, nine and seven genomes were not found to map at all on the candidates 5 and 27, respectively (Table 2**Error! Reference source not found.**). However, since the missing genome lists of these two markers were complementary to each other, these two markers were kept designing a potential duplex. Candidate 23 on the other hand, was fully inclusive with all 120 TR4 genomes, although mapping depth was low in some regions of the candidate sequence.

Coverage over non-target genomes was below 10% for all the three candidate regions (Full details on Detailed **information of the DNA isolates used in this study.**

S4 Table) except for CAV_36117 (Clade B, Foc-SC04), which mapped with a 12.6% and 43.7% coverage on the sequences of Candidate 5 and 23 respectively, and CAV_612 (Clade A, Foc-SC01) , mapping with a 55.1% of coverage on Candidate 27.

Field Code Changed

Field Code Changed

Formatted: Font: Bold

Field Code Changed

Formatted: Font: Not Bold

Table 2: ***In silico*** **specificity of candidate Foc-TR4 target sequence regions and LAMP amplicons of those previously published.**

Reference sequence used for alignment / BLAST queries										Genomes used		Internal BLAST results of reference sequences on all assembled genomes (Identity %)			Mapping results of Illumina reads on the Reference Sequences		
Marker Region	AT[a]	ST[a]	L[a]	MC[a]	Mask.[a]	Loc.[a]	Position in CAV807 / UK0001 reference genomes: Contig	Start	End	Status	NTBH[b]	100%	95-100%	90-95%	GFM[c]	GPM[c]	GNM[c]
**Cand5**	LAMP	Amplicon	214	No	No	Inter	contig_12 / VMNF01000005.1	1486738 / 5279483	1486951 / 5279270	Target	No	133	0	0	111	0	9
										Non Target		0	0	0	0	2	46
**Cand23**	LAMP	Amplicon	247	No	No	Inter	contig_3 / VMNF01000003.1	4477508 / 4455382	4477754 / 4455628	Target	No	119	0	0	118	2	0
										Non Target		0	0	0	0	10	38
**Cand27**	LAMP	Amplicon	234	No	No	Inter	contig_2 / VMNF01000004.1	32096 / 5203206	32329 / 5202973	Target	Yes	133	0	0	112	1	7
										Non Target		0	0	0	0	7	41
**Ordoñez et al. (2019)**	LAMP	Amplicon	214	No	No	Inter	contig_11 / VMNF01000015.1	623539 / 2103661	623752 / 2103448	Target	Yes	140	0	0	118	0	2
										Non Target		0	**1**	0	**1**	3	44
**Canare et al. (2024)**	LAMP	Amplicon	213	Yes	No	Inter	contig_12 / VMNF01000014.1	842769 / 2814708	842981 / 2814920	Target	Yes	142	0	2	120	0	0
										Non Target		**17**	**2**	17	**17**	5	26
**Li et al. (2013)**	LAMP	Amplicon	213	No	Yes	Genic	contig_6 / VMNF01000005.1	603139 / 613080	603351 / 613292	Target	Yes	143	0	0	120	0	0
										Non Target		**3**	**25**	0	**25**	20	3
**Zhang et al. (2013)**	RT-LAMP	Amplicon	472	Yes	No	Inter	contig_3 / VMNF01000010.1	5266 / 17344	5737 / 16873	Target	Yes	27	4	0	120	0	0
										Non Target		0	**22**	3	**48**	0	0
**Peng et al. (2014)**	RT-LAMP	Amplicon	217	No	Yes	Genic	contig_6 / VMNF01000005.1	603149 / 613090	603365 / 613306	Target	Yes	143	0	0	120	0	0
										Non Target		**3**	**25**	0	**27**	19	2

[a]Summary of the genome locations, BLAST and sequence alignment results. AT: Assay Type; ST: Sequence Type; L: length (bp); MC: Multiple Copies; Mask.: Located in repeat-masked regions; Loc.: location, genic or intergenic (inter)

[b]NTBH: Any Non Target BLAST hit (any size),

[c]GFM: Genomes fully mapped (100% coverage); GPM: genomes partially mapped; GNM: Genomes not mapped (0% coverage).

In silico **specificity analysis of candidate regions vs. previously published regions**

The approach of internal BLAST and Illumina alignments (Steps 2 and 4 in Fig 1), initially tested on the new candidates, was also applied on already published TR4 diagnostics assays by selecting the amplicon sequences of these systems (Table 2, details on Detailed **information of the DNA isolates used in this study.**

S4 Table). The genomic locations of the different markers on the genomes UK0001 and CAV807 are shown on Fig 2. Considering LAMP systems, none of the published regions were found to be fully specific to our genome panel.

The Ordóñez LAMP amplicon [37] ~~published by Ordóñez *et al.* 2019~~ showed the least amount of false positive detections, but had hits of 95-100% sequence identity to eight non-target genomes when tested on the internal BLAST dataset, most notably sequence identity of 97.2% to isolate CAV 225, an *F. oxysporum* isolated soil in banana plantation in South Africa. In addition, the Ordóñez ~~*et al.* 2019~~ ~~[37][40]~~ system [37] did not have any sequence alignment with two Foc-TR4 Illumina genomes: SHN19, and FJ41 from China. Considering the other four LAMP systems studied (Table 2), they all showed good inclusivity, by mapping with all 120 Illumina TR4 genomes, but were found to be less specific than Ordóñez *et al.* (2019). The Canare *et al.* 2024 marker [50] [~~48]~~ showed 100% sequence identity with 17 non-targeted assembled genomes, most of which were STR4, VCG 0121s, and three Australian unknown VCG Foc isolates. It also showed full alignment with 17 non-target Illumina genomes including various known Foc VCGs (0120, 0121, 0122, 0126, 01215, 01219). The LAMP target reported from [36] ~~Zhang *et al.* (2013) LAMP amplicon[18]~~, designed based on the IGS gene regions [18]~~[18]~~, showed a 100% alignment with 48 non-target Illumina genomes, and a BLAST identity between 95-100% in 40 non-target alignments, including Foc isolates identified as STR4, Race 1 and isolates in other Foc VCGs. The LAMP amplicons of Li *et al.* (2013), and Peng *et al.* (2014) [19, 35], located nearby close to the amplicon of the RT PCR Lin *et al.* (2013) [51], were

Formatted: Font: Italic

Field Code Changed

Field Code Changed

Formatted: Font: Bold

Field Code Changed

Formatted: Font: Not Bold

Field Code Changed

Field Code Changed

found in full alignment within 25 and 27 non-target Illumina genomes, respectively. When analysed in BLAST both gene targets showed a 100% identity within three VCG 0121 genomes (TWN0005, TWN0004, and TWN0003, and showed a 95-100% identity with 25 non-target genomes.

We also benchmarked other non-LAMP system amplicons (Detailed **information of the DNA isolates used in this study.**

S4 Table). The qPCR amplicon published by Matthews *et al.* 2020, showed the best inclusivity-exclusivity, although it was found to be located in a repeats-rich region of the genome (thus masked by RepeatMasker). The Aguayo qPCR amplicon [20] ~~of Aguayo *et al.* (2017)~~ showed a 100% BLAST identity on the three VCG 0121 genomes (TWN0005, TWN0004, and TWN0003), and high coverage on 47 non-target genomes, mostly with Race 4 isolates.

Primer design and initial validation

A first screening ~~(either by PCR and qPCR, data not shown)~~ was done on a subset of the specificity DNA panel, confirming that the best candidates were Candidate 5, 23 and 27 (respectively named Cand-5, Cand-23~~Cand-27~~, and Cand-27~~Cand-23 respectively~~). LAMP primers were then designed on the three candidates to be tested in the specificity panel.

Due to the limited size of the highly specific part of the Cand-23 fragment, only LAMP primer sets including FIP, BIP, F3, and B3 could be designed using the primer explorer software, and a Loop and two swarm primers [52] designed manually, were included in the final version of the Candidate 23 LAMP assay (

Formatted: Font: Bold

Field Code Changed

Formatted: Font: Not Bold

Formatted: Font: Times New Roman

Formatted: Font: Times New Roman

Formatted: Font: Times New Roman

Table **3. Error! Reference source not found.).**

Formatted: Font: Times New Roman

Formatted: Font: Times New Roman

Formatted: Font: Times New Roman

Fig 3. Position and direction of the LAMP primers on the Cand-23 region.

Table 3. Primer sequences and concentration used for Cand-23 LAMP assay.

Primer	Sequence	FC[a]
FIP-cand23	AAGCACTTATAAAACCCGGCCTCGAAGTAAACTAACTAGGGCAAC	1.6
BIP-cand23	AAGCTGGCTTAGCCTTAAAAGCTCCTATTAAGGGCAAAGAGAC	1.6
Loop-cand23	TTCTAATTACTTTTATTAGCACTGC	0.8
Swam_F1c-cand23	AAGCACTTATAAAACCCGGCCTC	0.8
SwarmB1c-cand23	AAGCTGGCTTAGCCTTAAAAGC	0.8
F3-cand23	TTAGAGGTATAAGGAGGCTTAG	0.4
B3-cand23	GCCTTTACTTAGTAATCTTTTACCT	0.4

[a]FC: Final concentration (µM), required in the final 25 µL reaction.

LAMP Primer Specificity

The Cand-23 LAMP assay was the most specific one ( ), only amplifying true positives and not amplifying any non-target isolates among the 161 strains tested. Cand-5 LAMP assay (121 strains tested) amplified 3 false positives and 7 false negatives, which were some of the genomes found absent *in silico*. This would confirm that their absence was genuine rather than due to sequencing artefacts. Cand-27 showed one 1 false negative (62 strains tested), and therefore, the Cand-23 LAMP assay was chosen for the subsequent tests. Considering other molecular detection tools that were analysed in this study, the Matthews qPCR [21] ~~Matthews *et al.* (2020),~~ was found to be fully specific among 104 isolates tested. The Aguayo qPCR [20]~~Aguayo *et al.* (2017)~~ amplified all target isolates, and 15 non-target (VCG 0121) isolates. This was not surprising as it is known that this system also amplifies VCG 0121. The Ordoñez LAMP assay [37]~~designed by Ordoñez *et al.* (2018)~~ (158 isolates tested) picked up all Foc-TR4 target isolates but also amplified 3 non-target strains: CAV 531 (endophyte), CAV 615 (VCG 01227), and Fova1185, a *Fusarium oxysporum* isolated from vanilla, respectively. Detailed results of the specificity are shown in S5 Table.

Field Code Changed

Formatted: Font: Not Bold

Table 4. Specificity results of the LAMP markers designed in this project (Cand-23, Cand-5, and Cand-27), compared with three current TR4 diagnostic reference methods.

Isolates tested					Result of different Foc TR4 diagnostic systems											
					Existing systems						This study					
Status	Species[a]	Clade / Foc-SC[b]	VCG[a]	Race[a]	qPCR1[c]		qPCR2[c]		LAMP3[c]		LAMP Cand-23		LAMP Cand-5		LAMP Cand-27	
					+[d]	-[d]	+[d]	-[d]	+[d]	-[d]	+[d]	-[d]	+[d]	-[d]	+[d]	-[d]
Non Target	*F. oxysporum* f. sp. *cubense*	A/1	0120	STR4	0	2	0	2	0	6	0	6	0	3	0	2
			0120/15	UNK	0	3	0	3	0	5	0	5	0	3	0	1
			01210	UNK	0	1	0	1	0	1	0	1	0	1	0	0
			01219	UNK	0	0	0	0	0	1	0	1	0	1	0	1
			0122	UNK	0	2	0	2	0	2	0	2	1	1	0	2
			0129/11	UNK	0	1	0	1	0	2	0	2	0	2	0	1
			01215	UNK	0	0	0	0	0	1	0	1	0	1	0	1
			01227	UNK	0	0	0	0	1	0	0	1	0	1	0	1
			0126	UNK	0	1	0	1	0	1	0	1	0	1	0	1
			0129	UNK	0	0	0	0	0	1	0	1	0	1	0	1
			SMV 1	UNK	0	0	0	0	0	1	0	1	0	1	0	1
			SMV 2	UNK-	0	0	0	0	0	1	0	1	0	1	0	1
		A/2	0121	Race 4	8	0	0	8	0	9	0	9	0	9	0	3
Target			01213/16	TR4	23	0	21	0	36	0	37	0	17	7	11	1
			N.D.	TR4*	9	0	9	0	9	0	9	0	0	0	0	0
Non Target			SMV 3	UNK-	0	0	0	0	0	1	0	1	1	0	0	1

Formatted Table

		01212	Race 1/2	0	1	0	1	0	1	0	1	0	1	0	1
F. oxysporum f. sp. *cubense*	B/4	01220	UNK	0	0	0	0	0	1	0	1	0	1	0	1
		01222	UNK	0	0	0	0	0	1	0	1	0	1	0	1
		0124	UNK	0	2	0	2	0	3	0	3	0	3	0	1
		0124/25	UNK	0	1	0	1	0	1	0	1	0	0	0	0
		0124/5	UNK	0	0	0	0	0	2	0	2	0	2	0	2
		0124/5/8	UNK	0	1	0	1	0	1	0	1	0	1	0	0
		0128	UNK	0	1	0	1	0	3	0	3	0	3	0	2
		ND	UNK	0	3	0	3	0	3	0	3	0	1	0	1
		01228	UNK	0	0	0	0	0	1	0	1	0	1	0	1
		01229	UNK	0	0	0	0	0	1	0	1	0	1	0	1
		01232	UNK	0	0	0	0	0	1	0	1	0	1	0	1
		01233	UNK	0	0	0	0	0	1	0	1	0	1	0	1
		01236	UNK	0	0	0	0	0	1	0	1	0	1	0	1
		0125	UNK	0	1	0	1	0	1	0	1	1	0	0	0
		0128/20	UNK	0	0	0	0	0	1	0	1	0	1	0	1
		SMV 4	UNK	0	0	0	0	0	1	0	1	0	1	0	1
		SMV 5	UNK	0	0	0	0	0	1	0	1	0	1	0	1
		SMV 6	UNK-	0	0	0	0	0	1	0	1	0	1	0	1
	B/5	01214	UNK	0	4	0	4	0	5	0	5	0	3	0	1
		01231	UNK-	0	0	0	0	0	1	0	1	0	1	0	1
	B/6	0123	Race 1	0	1	0	1	0	2	0	2	0	2	0	2
	B/7	01218	Race 1	0	0	0	0	0	1	0	1	0	1	0	1
		01234	UNK	0	0	0	0	0	1	0	1	0	1	0	1
		01235	UNK	0	0	0	0	0	1	0	1	0	1	0	1
		SMV 10	UNK-	0	0	0	0	0	1	0	1	0	1	0	1
	B/8	01230	Race 1	0	0	0	0	0	1	0	1	0	1	0	1
	C/3	01226	Race 1	0	3	0	3	0	3	0	3	0	3	0	3

UNK	Race 1	0	5	0	5	0	5	0	5	0	2	0	0
F. fujikuroi		0	2	0	2	0	2	0	2	0	2	0	0
F. graminearum		0	1	0	1	0	1	0	1	1	0	0	0
F. oxysporum	Endophyte	0	2	0	2	1	2	0	3	0	3	0	2
	UNK	0	7	0	7	0	8	0	8	0	7	0	1
F. oxysporum ~~f.sp.~~f. sp. *cepae*		0	2	0	2	0	2	0	2	0	2	0	0
F. oxysporum ~~f.sp.~~f. sp. *cyclamini*		0	1	0	1	0	1	0	1	0	1	0	0
F. oxysporum ~~f.sp.~~f. sp. *lini*		0	1	0	1	0	1	0	1	0	1	0	0
F. oxysporum ~~f.sp.~~f. sp. *lycopersici*		0	1	0	1	0	1	0	1	0	1	0	0
F. oxysporum ~~f.sp.~~f. sp. *medicaginis*		0	1	0	1	0	1	0	1	0	1	0	0
F. oxysporum ~~f.sp.~~f. sp. *melonis*		0	1	0	1	0	1	0	1	0	1	0	0
F. oxysporum ~~f.sp.~~f. sp. *radicis-lycopersici*		0	1	0	1	0	1	0	1	0	1	0	0
F. oxysporum ~~f.sp.~~f. sp. *radicisvanilla*		0	1	0	1	0	1	0	1	0	1	0	0
F. oxysporum ~~f.sp.~~f. sp. *vanilla*		0	2	0	2	1	1	0	2	0	2	0	0
F. oxysporum ~~f.sp.~~f. sp. *vasinfectum*		0	1	0	1	0	1	0	1	0	1	0	0
F. oxysporum Fo47		0	1	0	1	0	1	0	1	0	1	0	0
F. oxysporum PHRC474		0	1	0	1	0	1	0	1	0	1	0	0
Fusarium ~~f.sp.~~f. sp.		0	2	0	2	0	2	0	2	0	0	0	0
Pseudocercospora fijiensis		0	3	0	3	0	3	0	3	0	3	0	0
Ralstonia solanacearum		0	0	0	0	0	0	0	1	0	0	0	0
**Total Counts**		**40**	**64**	**30**	**72**	**48**	**110**	**46**	**114**	**21**	**100**	**11**	**51**

[a] Abbreviations: "F": *Fusarium*, "TR4*": TR4 verified by PCR, "N.D." Not determined, "UNK": Unknown, "SMV": Single-Member VCG.

[b] The Clade and VCG classification is based on Mostert et al., 2022 [27]~~[27]~~.

[c] qPCR1: Aguayo *et al*., 2017 [20]~~[20]~~; qPCR2: Matthews *et al*., 2020 [21]~~[21]~~; LAMP3: Ordoñez *et al*., 2019 [37]~~[40]~~.

[d] Results column meanings: +: Positive, -: Negative.

LAMP Primer Sensitivity and ~~optimization~~optimisation

Preliminary optimisation of Cand-23 LAMP primer concentrations and combinations allowed the selection of a primer mix (PM) consisting of doubled initial standard concentrations (FIP and BIP at 1.6 µM; SwarmF1c, SwarmB1c and Loop primers at 0.8 µM; and B3 and F3 at 1.4 µM) (Table 3), as this configuration reduced the average TTR by 1 min 45 s (S2 Fig).

~~Prior to sensitivity tests, optimization of different Cand-23 LAMP primer concentrations and combinations were conducted (data not shown). The chosen primer mix (PM) concentration consisted of doubling the initial standard concentrations (FIP, BIP at 1.6 µM, SwarmF1c, SwarmB1c and Loop primer at 0.8 µM, and B3 and F3 at 1.4 µM) (Table 3), as it was shown to reduce the average TTR by 01:45 minutes (S2 Fig).~~

Formatted: Font: Times New Roman, 12 pt

Formatted: Highlight

Formatted: Font: Not Bold

Formatted: Font: Not Bold

Field Code Changed

Field Code Changed

Formatted: Font: Not Bold

Formatted: Font: Not Bold

Sensitivity of the Cand-23 LAMP assay was tested on plasmid dilutions prepared in water (Fig 4, a) and on DNA extractions of healthy plant tissue spiked with plasmid dilutions (Fig 4, b). In both sample sets, the limit of detection (LOD) was reached consistently at 10^2 copies~~.~~ μL^{-1}. When a volume of 5 µL per ~~/~~reaction of the plasmid DNA was used (blue points in the graphs), TTR averaged 18:07 min, and Annealing temperature (At) of 82.8 °C for plasmid dilutions in water, and average TTR 13:41 min at 82.3 °C in plant tissue. Surprisingly, when sensitivity were conducted on samples were plant tissue were spiked with plasmid dilutions a better detection limit were recorded at 10^1 copies ~~.~~μL^{-1} using 5µL per~~/~~ reaction, that is 50 copies per reaction (average TTR 20:32 ~~min, 82.3~~min, 82.3 °C in Fig 4, b).

Field Code Changed

Field Code Changed

Fig 4. Sensitivity of the Cand-23-LAMP system as assessed in plasmid dilutions (a) and in plasmid-spiked plant tissue extractions (b). TTR: Time To Result (mins:ss). The colour and shape of the data points indicates the DNA volume used in the reaction; blue triangles (5µL) or yellow squares (1µL) for TTR means, while replicates are shown as solid circles. Error bars indicate the

Standard Error (SE) of the mean. The horizontal black line indicates the threshold above which a sample is considered negative, and the dashed red horizontal line indicates the value assigned to the samples that produced null signal (negative). The asterisks on top indicate whether the mean is positive with a 95% of confidence level.

Additionally, DNA extractions of conidial dilutions mixed with healthy plant material were tested to compare the Cand-23 LAMP assay with the Ordoñez LAMP assay [37]~~published by Ordoñez *et al*., (2019)[40]~~, and two qPCR assays [20, 21]~~, published by Aguayo *et al*. (2017) [20] and Matthews *et al*. (2020) [21], respectively~~. For both LAMP systems tested, a LOD of 10^5 cells. μL^{-1} were reached. An average TTR at 16:35 min and annealing peak at 82.7 °C were recorded for Cand-23 LAMP system, while for the Ordoñez ~~et al. (2019)~~ LAMP assay [37], an average TTR 15:20 min, and annealing peak of recorded at 84.5 °C (S3 Fig). Collectively, these results indicated that both LAMP systems display similar sensitivity performances. Considering the qPCR assays, the Matthews *et al*., 2020 SYBR green qPCR could not detect any of the samples from this extraction, whilst the Aguayo system reached a LOD of 10^5 cells. μL^{-1}. The detailed results of all sensitivity tests can be found in S6 Table.

Field Code Changed

Formatted: Font: Not Bold

Formatted: Font: Not Bold

Field Code Changed

Detection from artificially and naturally infected plant material

In glasshouse conditions, all the healthy and asymptomatic plants tested negative. In the field, the situation was more complex because the presence of other pathogens, particularly the bacterium causing Banana Blood Disease, produced confusing symptoms (yellowing and wilting of leaves, internal vascular discoloration), making the correlation between symptoms and the presence of Foc-TR4 unclear. In Java, Foc-TR4 was detected by both LAMP assays and the qPCR assay in only 10 samples out of 66, which had been collected from seven banana plants of the cultivars Pisang Barangan, Balbisiana, and Pisang Raja, located in Cianjur. A perfect correspondence of results was obtained between the Cand-23 LAMP, and the qPCR results performed on samples from Comoros

and Vietnam (Fig 5). Moreover, these results agreed with the VCG determined from the isolated fungi, when available (S7 Table).

The Cohen's kappa index, reflecting the agreement between Cand-23 and qPCR, varied between different datasets (Fig 5a, Table 5 and S7 Table) from 0.65 (substantial) in the glasshouse (n=34), 0.49 (moderate) for in-field sampling done in Indonesia (n=66), up to 1 (perfect) in Comoros (n=8) and Vietnam (n=8). Considering all the field data, the kappa index was 0.64, with a total agreement of 84.1%, indicating a substantial agreement with the reference method. However, the results obtained from field data between Cand-23 LAMP and qPCR were significantly different (McNemar's test p=0.0265), probably due to the higher positives detected by qPCR compared to LAMP. This discrepancy is reflected in the diagnostics Sensitivity (63.3%) whereas the Specificity shows consistently high values (96.4%). For the glasshouse experiment, results were similar with a total agreement of 82.4%, 0.65 kappa index, but not significant McNemar p-value (0.6831). The glasshouse and Indonesia samples were also tested with Ordóñez et al (2019) LAMP [37] to compare with Cand-23 LAMP (S7 Table and Fig 5b) No significant differences were obtained between the two methods and the different parameters reflected a hight agreement between the two methods: an overall kappa coefficient of 0.87, , and sensitivity and specificity values of 89.5% and 97.1%, respectively.

Fig 5. Cand-23 *in planta* result comparison. Comparison of the results obtained with the Cand-23 LAMP assay compared to the results with: (a) the Foc-TR4 specific qPCR as reported by Matthews *et al*., (2020)[21] [21] and (b) Foc-TR4 specific Ordóñez LAMP assay as reported by Ordóñez *et al*., (2019) [37][40]. The colour of the data points refers the origin of the data (naturally infected samples in Indonesia, Vietnam of Comoros, or artificially inoculated material in the Glasshouse) and indicates the average of the different replicates.

Field Code Changed
Formatted: Font: Not Bold
Field Code Changed
Field Code Changed
Field Code Changed
Field Code Changed
Formatted: Font: Not Bold
Field Code Changed
Formatted: Font: Not Bold

Table 5: Summary of the results obtained by the Cand-23 LAMP compared to qPCR assay as published by Matthews *et al*. (2020).

	**Field_Comoros**		**Field_Indonesia**		**Field_Vietnam**		**Glasshouse**		**TOTAL FIELD**		**TOTAL**	
	**LAMP +**	**LAMP -**	**LAMP +**	**LAMP -**	**LAMP +**	**LAMP -**	**LAMP +**	**LAMP -**	**LAMP +**	**LAMP -**	**LAMP +**	**LAMP -**
**qPCR +**	6	0	10	11	3	0	15	4	19	11	34	15
**qPCR -**	0	2	2	43	0	5	2	13	2	50	4	63
**n. Samples**	6	2	12	54	3	5	17	17	21	61	38	78
**Total n. Samples**	8		66		8		34		82		116	
**Pos. agreement**	100%		83.3%		100%		88.2%		90.5%		89.5%	
**Neg. agreement**	100%		79.6%		100%		76.5%		82%		80.8%	
**Total agreement**	100%		80.3%		100%		82.4%		84.1%		83.6%	
**Cohen's kappa (± SD)**	**1 (±0)**		**0.49 (±0.12)**		**1 (±0)**		**0.65 (±0.13)**		**0.64 (±0.09)**		**0.65 (±0.07)**	
**McNemar's Chi-sq.**			4.92				0.17		4.92		5.26	
**McNemar's p-value (Chi-sq.)**			0.0265				0.6831		0.0265		0.0218	
**Sensitivity (SE)**	100%		47.6%		100%		78.9%		63.3%		69.4%	
**Specificity (SP)**	100%		95.6%		100%		86.7%		96.2%		94%	
**Accuracy (AC)**	100%		80.3%		100%		82.4%		84.1%		83.6%	
**Positive Predictive Value (PPV)**	100%		83.3%		100%		88.2%		90.5%		89.5%	
**Negative Predictive Value (NPV)**	100%		79.6%		100%		76.5%		82%		80.8%	

Results are presented for artificially inoculation conducted under glasshouse conditions, as well as naturally infected samples analysed collected in Indonesia, Comoros, and Vietnam. Parameter description: Sensitivity (SE) = TP / (TP + FN): proportion of true positives correctly detected by LAMP. Specificity (SP) = TN / (TN + FP): proportion of true negatives correctly identified. Positive Predictive Value (PPV) = TP / (TP + FP): probability that a positive LAMP result is a true positive. Negative Predictive Value (NPV) = TN / (TN + FN): probability that a negative LAMP result is a true negative. Accuracy (AC) = (TP + TN) / (TP + TN + FP + FN): overall proportion of correctly classified samples. Cohen's Kappa ≤ 0 corresponds to no agreement, 0.01–0.20 is "none to slight", 0.21–0.40 is "fair", 0.41– 0.60 is "moderate", 0.61–0.80 is "substantial", and 0.81–1.00 is "near-perfect". A McNemar's p

value below 0.05 was taken as evidence to reject the null hypothesis of equal marginal proportions, indicating a statistically significant difference in detection outcomes between the two methods compared.

Discussion

Foc-TR4 continues to expand globally, with latest detections reported in Latin America (Colombia, Peru and Venezuela) [14][14] and Africa (Mayotte [53], [53] and Comoros islands [54] [54]). The continued spread poses a significant threat to the global banana industry due to the lack of effective curative measures against the disease once established. Consequently, there is an urgent need for a rapid and reliable diagnostic tool for early detection to implement timely containment strategies and mitigate the impact of the disease. In this study, we present a new real-time LAMP assay for Foc-TR4 VCG 01213/16 identification, which were robustly designed through comparative genomic approach and specificity testing on a diverse collection of 148 Foc-TR4 genomes, and 146 non-target genomes, including all known Foc VCGs and other closely *F. oxysporum* as well as other genera strains. This assay can be applied directly in-field and therefore, offer an improved tool that can be used for early detection in surveillance efforts or in containment strategies on-farm.

In this study the K-mer Elimination by Cross-reference (KEC) method [38] [35] was considered for robust identification of a Foc TR4 specific sequence region. The KEC method has previously been used to develop LAMP assays in plant pathogenic bacteria and fungi [55, 56][55,56], but not yet on *Fusarium oxysporum*. The KEC method enables unbiased, genome-wide identification of TR4-specific sequences by operating directly at the k-mer level, independently of gene annotation or pre-defined loci, thereby reducing reference bias compared with gene- or reference-based approaches. In addition, KEC allows the direct selection of candidate regions of a predefined minimum length, which is essential for LAMP primer design, where recommended target sizes range between 120 and 180 nucleotides [33][33]. This contrasts with other approaches used so far to develop Foc TR4 diagnostic assays, such as DArTseq sequencing which implies genome complexity reduction [21, 37][21,40], or the identification of polymorphisms in known *SIX* genes [50, 57][48,57], which are biased towards enzyme restrictions sites or genes, and yield short candidate sequences. These sequences often require to be extended to be able to design LAMP primers, at the risk of losing the specificity. Several

alternative marker discovery strategies were evaluated in this study, including ortho-group filtering and Illumina read mapping and filtering against a TR4 reference genome, but these approaches yielded few candidates and lacked sufficient specificity or inclusivity. In contrast, the KEC method generated multiple candidate regions, enabling downstream filtering and experimental validation to select a robust marker. Notwithstanding this advantage, the KEC method may also be limited by the quality of the input genome assemblies used, which might include poor genomic sequences such as high GC content regions, not appropriate for primer design. To overcome these limitations the reference genome (referred as “master” genome in KEC protocol) used was a high quality long-read assembly of an Foc-TR4 isolate. The repetitive element-masked version of the genome was used to avoid the selection of candidates within the repeat-rich regions of the genome which could potentially be unstable. One disadvantage of the KEC approach employed however, was that not all the predicted sequences were target specific, as BLAST searches showed matches in non-target genomes. The initial version (v1.0) of the KEC software was used in this analysis, in which the *include* mode applied less stringent criteria for sequence uniqueness than in later releases, potentially allowing retention of sequences not exclusively specific to the target. This limitation was nevertheless addressed by the addition of internal and external BLAST steps improved specificity, but full inclusivity on all the target Foc-TR4 genomes was still not achieved. This was probably due to the incompleteness of some Illumina assemblies, as (regions with low read depth and quality were excluded during the assembly process) [58][58]. Adding the Illumina read mapping step allowed to identify genuine genomic absences on certain Foc-TR4 isolates in some of the candidates, leading the selection of the Candidate 23, which was present in all 120 target genomes and absent in all non-target genomes representing all known *Foc* VCGs, as well as non-pathogenic and endophytic *F. oxysporum* species associated with banana. This multi-step validation demonstrated that the KEC-based strategy, combined with downstream filtering, provided a clear improvement in marker specificity and inclusivity compared to alternative approaches cited above. This was demonstrated experimentally when tested on a large specificity panel comprising Foc-TR4 isolates representatives from all known

other *Foc* VCGs, additional isolates belonging to the *F. oxysporum* species complex and fungal endophytes isolates from banana environment

The selected Candidate 23 is located within a unique intergenic region on Chromosome 2 of the reference II5 (GCA 031834405). The sequence was characterized by the presence of repetitive nucleotides. Additionally, read coverage was low, with a pronounced decrease observed in one part of the sequence. Due to the presence of repetitive nucleotides in the Candidate 23 target sequence, the design of LAMP primers with primer design software was not possible. This issue was resolved through the manual design of a loop primer and Swarm primers, a lesser-known alternative to loop primers that enhanced the LAMP reaction [52, 59]~~[52,59]~~. This allowed the development of a LAMP system that worked and provided a clear single annealing peak.

This study also analysed the specificity of other already published Foc-TR4 LAMP assays using the *in silico* approach. Amplicon sequences from Li *et al.* (2013) [19] showed good inclusivity in terms of BLAST and alignment on all Foc-TR4 Illumina genomes, but also had high (95-100%) identity and full alignment with 43 and 25 non-target genomes respectively, including 100% BLAST identity on three VCG 0121 genomes. A similar result was obtained with the LAMP assay designed by Canare *et al*. (2024) [50], consistent with the fact that this LAMP targets the *SIX8a* homologue gene, which is present in all Race 4 isolates [28, 60] ~~[28,60]~~. In contrast, the Ordóñez *et al.* (2019) LAMP assay [37]was not fully inclusive, not picking up two Foc TR4 genomes (both of Chinese origin). Whether this was a genuine lack or could have been caused by sequencing problems of these genomes, could not be validated on DNA; however, the correlation between the *in silico* and *in vitro* was good in the sixty-two isolates for which there was data and DNA. Also, similar cases of missing alignments on genomes for Candidate 5 were validated experimentally on seven DNA isolates. On the other hand, this LAMP system was not fully exclusive, showing high sequence homology to an *F. oxysporum* endophyte, and positives on non-target DNA isolates. The latter result stresses the importance of

performing thorough specificity testing especially considering endophytic, non-pathogenic isolates which might amplify as false positives. This is particularly challenging in fungal species with a broad host range such as *F. oxysporum* given the genetic similarity between non-pathogenic and endophytic isolates with pathogenic isolates [61] ~~[61]~~.

The sensitivity of our LAMP assay obtained a consistent LOD of up to 100 copies per~~/~~ reaction with plasmid dilutions, which is the same dilution as reported in other existing LAMP systems for Foc-TR4 [40]. Interestingly a better LOD of 50 copies~~/~~ per reaction was reached when testing the plasmid dilutions in a plant tissue background extraction. Plant tissue providing a matrix which would help plasmid DNA precipitation and binding, preventing degradation and DNA loss could possibly explain this difference with the tests carried out on simple water plasmid dilutions [62, 63]~~[62,63]~~. Nevertheless, this corroborates the fact that non-target DNA presence does not affect the assay performance.

The LAMP assay designed in this study was evaluated under glasshouse and field conditions across three countries (Indonesia, Vietnam, and Comoros), and it performed reliably using both commercial and simplified DNA extraction protocols. The tool's portability was also demonstrated through successful implementation in non-laboratory settings, highlighting its potential for use in resource-limited settings.

In comparison with the qPCR assays tested in parallel, our LAMP assay showed strong correlation; however, qPCR still proved to remain as the gold standard in terms of sensitivity. Nevertheless, this came with the trade-off of higher requirements in terms of laboratory, extraction equipment and time.

Conclusion

Extensive regulatory measures have been implemented to prevent the introduction and establishment of Foc-TR4 in unaffected areas. The effectiveness of this surveillance relies heavily on having specific and sensitive detection protocols. Although molecular-based methods are available, many have demonstrated issues with specificity. A novel highly specific LAMP assay was developed in this study for in-field detection of Foc-TR4, which was validated by comparative genomics approach. This approach accounts for the known genetic diversity within Foc and FOSC, improving upon the specificity of previously published Foc-TR4 detection assays to date. The LAMP assay was shown to be highly effective in detecting FocTR4 in infected banana plants, particularly in non-laboratory environments, offering a promising tool for improving disease management strategies. Implementing this technique regularly in diagnostic processes will allow early identification of infected banana plants, facilitating timely intervention to prevent widespread outbreaks. The Cand-23 LAMP assay is now commercialized in a ready-to-use "LAMP Foc-TR4 kit" produced by QUALIPLANTE SAS (Lavérune, France. https://www.qualiplante.eu), in an all-inclusive format containing the lyophilized primer mix, the lyophilized enzyme, a resuspensions buffer, and positive and negative controls.

Acknowledgements

~~This study was funded by the Projet France Relance (PFR) CIRAD-QUALIPLANTE (2022-2024), and by the European Union Horizon 2020 Research and Innovation Program Marie Sklodowska-Curie Fellowship, through the INDICANTS project grant nr 890856.~~ We thank Natalya Saveljeva, Veronique Roussel and Christine Pages for technical assistance, Lorène Milliex for the project management, Caroline Chatillon for assistance in Qualiplante, Megan Matthews for help in extractions and assistance. We also thank Nguyen Thi Thô for assistance and extractions, and Mauricio Soto-Suarez (Agrosavia) for having shared Foc-TR4 genome assemblies and long-reads ONT data from Colombia.~~.~~

References

Formatted: Font: 18 pt, Bold

Formatted: Normal, Line spacing: Double, Border: Top: (No border), Bottom: (No border), Left: (No border), Right: (No border), Between : (No border)

Formatted: Indent: Left: 0", Hanging: 0.39", Line spacing: Double

1. FAO. Together, we can prevent the spread of Tropical Race 4 (TR4). Food Agric Organ United Nations2020. p. 1-8.
2. Viljoen A, Ma L-J, Molina AB. CHAPTER 8: Fusarium Wilt (Panama Disease) and Monoculture in Banana Production: Resurgence of a Century-Old Disease. In: Ristaino JB, Records A, editors. Emerging Plant Diseases and Global Food Security. Saint Paul, MN, USA: APS Press; 2020. p. 159-84.
3. Munhoz T, Vargas J, Teixeira L, Staver C, Dita M. Fusarium Tropical Race 4 in Latin America and the Caribbean: status and global research advances towards disease management. Frontiers in Plant Science [Internet]. 2024 2024-July-16; 15: 1397617. Available from: https://www.frontiersin.org/journals/plant-science/articles/10.3389/fpls.2024.1397617/pdf.
4. Stover RH. Fusarial wilt (panama disease) of bananas and other Musa species: Commonwealth Mycological Institute; 1962. 117 p.
5. Ploetz RC. Fusarium Wilt of Banana Is Caused by Several Pathogens Referred to as *Fusarium oxysporum* f. sp. *cubense*. Phytopathology. 2006;96(6):653-6. doi: 10.1094/PHYTO-96-0653. PubMed PMID: 18943184.
6. Lescot T. Inquiétudes sur l'évolution géographique de la race 4 tropicale (TR4) de la maladie de Panama [Concerns over the geographic spread of tropical race 4 (TR4) of Panama disease]. Fruitrop. 2015;(231):68. PubMed PMID: Agritrop:581003; PubMed Central PMCID: PMCAxe 4 (2014-2018) - Santé des animaux et des plantes.
7. Dawson C, Loeillet D, Lescot T, Sanchez C. Recueil statistique Banane 2023. Document technique et de recherche. Montpellier, France: CIRAD, 2024 Contract No.: Agritrop:611936.
8. Voora V, Larrea C, Bermúdez S. Global Market Report: Bananas. International Institute for Sustainable Development, 2020.

9. Drenth A, Kema G. The Vulnerability of Bananas to Globally Emerging Disease Threats. Phytopathology. 2021;111(12):2146-61. doi: 10.1094/phyto-07-20-0311-rvw. PubMed PMID: 34231377.

10. Ploetz RC. Panama disease: Return of the first banana menace. Int J Pest Manage. 1994;40(4):326-36. doi: 10.1080/09670879409371908.

11. Dita M, Barquero M, Heck D, Mizubuti ESG, Staver CP. Fusarium Wilt of Banana: Current Knowledge on Epidemiology and Research Needs Toward Sustainable Disease Management. Frontiers in Plant Science [Internet]. 2018 Oct 19; 9: 1468. Available from: https//doi.org/10.3389/fpls.2018.01468.

12. Waraczewski R, Sołowiej BG. May bananas go extinct? The epidemic of Panama disease. A review. Agronomy Science. 2022;77(3):139-48. doi: 10.24326/as.2022.3.10.

13. Viljoen A, Mostert D, Chiconela T, Beukes I, Fraser C, Dwyer J, et al. Occurrence and spread of the banana fungus *Fusarium oxysporum* f. sp. *cubense* TR4 in Mozambique. S Afr J Sci [Internet]. 2020 11/26; 116(11/12). Available from: https://sajs.co.za/article/view/8608.

14. Martínez G, Olivares BO, Rey JC, Rojas J, Cardenas J, Muentes C, et al. The Advance of Fusarium Wilt Tropical Race 4 in Musaceae of Latin America and the Caribbean: Current Situation. Pathogens [Internet]. 2023 Feb; 12(2): 277. Available from: https://10.3390/pathogens12020277.

15. Maymon M, Sela N, Shpatz U, Galpaz N, Freeman S. The origin and current situation of *Fusarium oxysporum* f. sp. cubense tropical race 4 in Israel and the Middle East. Sci Rep [Internet]. 2020 Jan 31; 10(1): 1590. Available from: https://www.nature.com/articles/s41598-020-58378-9.pdf.

16. Sharrock S, Frison E. Musa production around the world - trends, varieties and regional importance. Focus Paper 2. Montpellier, France: INIBAP, 1998.

17. Pegg KG, Coates LM, O’Neill WT, Turner DW. The Epidemiology of Fusarium Wilt of Banana. Frontiers in Plant Science [Internet]. 2019; 10: 1395. Available from: https://doi.org/10.3389/fpls.2019.01395.

18. Dita MA, Waalwijk C, Buddenhagen IW, Souza MT, Kema GHJ. A molecular diagnostic for tropical race 4 of the banana fusarium wilt pathogen. Plant Pathology. 2010;59(2):348-57. doi: 10.1111/j.1365-3059.2009.02221.x. PubMed PMID: WOS:000275107800015.

19. Li BJ, Du JL, Lan CZ, Liu PQ, Weng QY, Chen QH. Development of a loop-mediated isothermal amplification assay for rapid and sensitive detection of *Fusarium oxysporum* f. sp c*ubense* race 4. Eur J Plant Pathol. 2013;135(4):903-11. doi: 10.1007/s10658-012-0136-9. PubMed PMID: WOS:000315601500026.

20. Aguayo J, Mostert D, Fourrier-Jeandel C, Cerf-Wendling I, Hostachy B, Viljoen A, et al. Development of a hydrolysis probe-based real-time assay for the detection of tropical strains of *Fusarium oxysporum* f. sp. *cubense* race 4. Plos One [Internet]. 2017; 12(2): e0171767. Available from: https://www.ncbi.nlm.nih.gov/pmc/articles/PMC5298334/pdf/pone.0171767.pdf.

21. Matthews MC, Mostert D, Ndayihanzamaso P, Rose LJ, Viljoen A. Quantitative detection of economically important *Fusarium oxysporum* f. sp. *cubense* strains in Africa in plants, soil and water. Plos One [Internet]. 2020; 15(7): e0236110. Available from: https://journals.plos.org/plosone/article/file?id=10.1371/journal.pone.0236110&type=printable.

22. Roberts JM, Carvalhais LC, O'Dwyer C, Rincón-Flórez VA, Drenth A. Diagnostics of Fusarium wilt in banana: Current status and challenges. Plant Pathology. 2024;73(4):760-76. doi: https://doi.org/10.1111/ppa.13863.

23. Ma LJ, van der Does HC, Borkovich KA, Coleman JJ, Daboussi MJ, Di Pietro A, et al. Comparative genomics reveals mobile pathogenicity chromosomes in Fusarium. Nature

[Internet]. 2010 Mar 18 PMC3048781]; 464(7287). Available from: https://www.nature.com/articles/nature08850.pdf.

24. Magdama F, Monserrate-Maggi L, Serrano L, Sosa D, Geiser DM, Jimenez-Gasco MDM. Comparative analysis uncovers the limitations of current molecular detection methods for *Fusarium oxysporum* f. sp. *cubense* race 4 strains. Plos One [Internet]. 2019; 14(9): e0222727. Available from: https://journals.plos.org/plosone/article/file?id=10.1371/journal.pone.0222727&type=printable.

25. O'Donnell K, Sutton DA, Rinaldi MG, Magnon KC, Cox PA, Revankar SG, et al. Genetic diversity of human pathogenic members of the *Fusarium oxysporum* complex inferred from multilocus DNA sequence data and amplified fragment length polymorphism analyses: evidence for the recent dispersion of a geographically widespread clonal lineage and nosocomial origin. J Clin Microbiol. 2004;42(11):5109-20. Epub 2004/11/06. doi: 10.1128/JCM.42.11.5109-5120.2004. PubMed PMID: 15528703; PubMed Central PMCID: PMCPMC525153.

26. O'Donnell K, Kistler HC, Cigelnik E, Ploetz RC. Multiple evolutionary origins of the fungus causing Panama disease of banana: concordant evidence from nuclear and mitochondrial gene genealogies. Proc Natl Acad Sci U S A. 1998;95(5):2044-9. PubMed PMID: 9482835; PubMed Central PMCID: PMCPMC19243.

27. Mostert D, Wicker E, de Jager MM, Al Kaabi SM, O’Neill WT, Perry S, et al. A Polyphasic Approach Reveals Novel Genotypes and Updates the Genetic Structure of the Banana Fusarium Wilt Pathogen. Microorganisms [Internet]. 2022; 10(2): 269. Available from: https://www.mdpi.com/2076-2607/10/2/269.

28. Czislowski E, Fraser-Smith S, Zander M, O'Neill WT, Meldrum RA, Tran-Nguyen LTT, et al. Investigation of the diversity of effector genes in the banana pathogen, *Fusarium oxysporum* f.

sp. *cubense*, reveals evidence of horizontal gene transfer. Mol Plant Pathol. 2018;19(5):1155-71. doi: 10.1111/mpp.12594. PubMed PMID: 28802020.

29. Zheng S-J, García-Bastidas F, Li X, Zeng L, Bai T, Xu S, et al. New Geographical Insights of the Latest Expansion of *Fusarium oxysporum* f.sp. *cubense* Tropical Race 4 Into the Greater Mekong Subregion. Front Plant Sci [Internet]. 2018; 9: 457. Available from: https://doi.org/10.3389/fpls.2018.00457.

30. Torres Bedoya E, Bebber DP, Studholme DJ. Taxonomic Revision of the Banana Fusarium Wilt TR4 Pathogen Is Premature. Phytopathology. 2021;111(12):2141-5. doi: 10.1094/phyto-03-21-0089-le. PubMed PMID: 34100303.

31. Maryani N, Lombard L, Poerba YS, Subandiyah S, Crous PW, Kema GHJ. Phylogeny and genetic diversity of the banana Fusarium wilt pathogen *Fusarium oxysporum* f. sp. *cubense* in the Indonesian centre of origin. Stud Mycol [Internet]. 2019 Mar; 92. Available from: https://www.ncbi.nlm.nih.gov/pmc/articles/PMC6086327/pdf/main.pdf.

32. EFSA PoPH, Bragard C, Baptista P, Chatzivassiliou E, Di Serio F, Gonthier P, et al. Pest categorisation of *Fusarium oxysporum* f. sp. *cubense* Tropical Race 4. EFSA Journal [Internet]. 2022; 20(1): e07092. Available from: https://efsa.onlinelibrary.wiley.com/doi/abs/10.2903/j.efsa.2022.7092.

33. Yang L, Sun Y, Sun L, Wang Z, Feng J, Liang Y. Application of Loop-Mediated Isothermal Amplification in Plant Pathogen Detection. Phytopathology. 2025;115(1):6-13. doi: 10.1094/phyto-10-23-0391-kc. PubMed PMID: 39348563.

34. Németh MZ, Kovács GM. A comprehensive guide to loop-mediated isothermal amplification, an emerging diagnostic tool for plant pathogenic fungi. Frontiers in Plant Science [Internet]. 2025 2025-May-22; 16: 1568657. Available from: https://www.frontiersin.org/journals/plant-science/articles/10.3389/fpls.2025.1568657.

35. Peng J, Zhang H, Chen F, Zhang X, Xie Y, Hou X, et al. Rapid and quantitative detection of *Fusarium oxysporum* f. sp. *cubense* race 4 in soil by real-time fluorescence loop-mediated

isothermal amplification. J Appl Microbiol. 2014;117(6):1740-9. doi: 10.1111/jam.12645. PubMed PMID: 25200557.

36. Zhang X, Zhang H, Pu JJ, Qi YX, Yu QF, Xie YX, et al. Development of a Real-Time Fluorescence Loop-Mediated Isothermal Amplification Assay for Rapid and Quantitative Detection of *Fusarium oxysporum* f. sp *cubense* Tropical Race 4 In Soil. Plos One [Internet]. 2013 Dec 20; 8(12): e82841. Available from: https://doi.org/10.1371/journal.pone.0082841.

37. Ordóñez N, Salacinas M, Mendes O, Seidl MF, Meijer HJG, Schoen CD, et al. A loop-mediated isothermal amplification (LAMP) assay based on unique markers derived from genotyping by sequencing data for rapid in planta diagnosis of Panama disease caused by Tropical Race 4 in banana. Plant Pathology. 2019;68(9):1682-93. doi: 10.1111/ppa.13093.

38. Beran P, Stehlíková D, Cohen SP, Čurn V. KEC: unique sequence search by K-mer exclusion. Bioinformatics [Internet]. 2021; 37(19): btab196. Available from: https://doi.org/10.1093/bioinformatics/btab196.

39. Bache S. Analysis of the genomic structures of several strains of the fungal plant pathogen *Fusarium oxysporum* f.sp. *cubense* [Master]. Montpellier, Strasbourg: Université de Strasbourg; 2022.

40. Mestiri Y. Outils d'annotation et de visualisation de structure génomique pour l'annotation et l'exploration du pangénome fongique. Application à *Fusarium oxysporum* f.sp. *cubense*, agent causal de la fusariose du bananier. Montpellier: Université de Montpellier; 2023.

41. Jackman SD, Vandervalk BP, Mohamadi H, Chu J, Yeo S, Hammond SA, et al. ABySS 2.0: resource-efficient assembly of large genomes using a Bloom filter. Genome Research. 2017;27(5):768-77. doi: 10.1101/gr.214346.116. PubMed PMID: WOS:000400392400011.

42. Robène I, Maillot-Lebon V, Pecrix Y, Ravelomanantsoa S, Massé D, Jouen E, et al. Loop-Mediated Isothermal Amplification Assays for Rapid Detection of

the *Ralstonia solanacearum* Species Complex and Phylotype I in Solanaceous Crops. Phytofrontiers. 2023;3(3):582-93. doi: 10.1094/Phytofr-08-22-0089-R. PubMed PMID: WOS:001369256500020.

43. ANSES. Détection des souches tropicales de *Fusarium oxysporum* f. sp. *cubense* race 4 (Foc TR4) par PCR en temps réel. Méthode d'analyse en santé des végétaux: ANSES-Laboratoire de la Santé des Végétaux, Laboratoire de référence "Champignons sur toute matrice"; 2018. p. 27.

44. Kelman A. The relationship of pathogenicity in *Pseudomonas solanacearum* to colony appearance on a tetrazolium medium. Phytopathology. 1954;44:693-5.

45. Matthews MC. The global spread and detection of *Fusarium oxysporum* f. sp. *cubense* tropical Race 4 [PhD]. Stellenbosch: Stellenbosch University; 2023.

46. Matthews MC, van der Linden J, Robène I, Rozsasi S, Coetzee B, Campa M, et al. A combined recombinase polymerase amplification CRISPR/Cas12a assay for detection of *Fusarium oxysporum* f. sp. *cubense* tropical race 4. Sci Rep-Uk [Internet]. 2025 2025/01/19; 15(1): 2436. Available from: https://doi.org/10.1038/s41598-025-85633-8.

47. Wanjala BW, Ateka EM, Miano DW, Fuentes S, Perez A, Low JW, et al. Loop-Mediated Isothermal Amplification assays for on-site detection of the main sweetpotato infecting viruses. J Virol Methods [Internet]. 2021 2021/12/01; 298: 114301. Available from: https://www.sciencedirect.com/science/article/pii/S0166093421002408.

48. McHugh ML. Lessons in biostatistics, Interrater reliability: the kappa statistic. Biochem Med (Zagreb). 2012;22(3):276-82. PubMed PMID: 23092060; PubMed Central PMCID: PMCPMC3900052.

49. Cao G, Lin K, Ai J, Cai J, Zhang H, Yu Y, et al. A diagnostic accuracy study comparing RNA LAMP, direct LAMP, and rapid antigen testing from nasopharyngeal swabs. Front Microbiol [Internet]. 2022 [cited The authors declare that the research was conducted in the absence of any commercial or financial relationships that could be construed as a potential conflict of

interest. PMC9813509]; 13: 1063414. Available from: https://doi.org/10.3389/fmicb.2022.1063414.

50. Canare Perry Lorraine D, Uy Roselyn J, Konishi S, Tonami H, Alvindia Dionisio G, Nakashima C. Development of Loop-Mediated Isothermal Amplification (LAMP) Kit for the Detection of Fusarium Wilt Using *SIX8* Genes. J Phytopathol [Internet]. 2024; 172(5): e13398. Available from: https://onlinelibrary.wiley.com/doi/abs/10.1111/jph.13398.

51. Lin YH, Su CC, Chao CP, Chen CY, Chang CJ, Huang JW, et al. A molecular diagnosis method using real-time PCR for quantification and detection of *Fusarium oxysporum* f. sp *cubense* race 4. Eur J Plant Pathol. 2013;135(2):395-405. doi: 10.1007/s10658-012-0096-0. PubMed PMID: WOS:000313163400016.

52. Botella JR. Point-of-Care DNA Amplification for Disease Diagnosis and Management. Annu Rev Phytopathol [Internet]. 2022; 60(1). Available from: https://www.annualreviews.org/doi/abs/10.1146/annurev-phyto-021621-115027.

53. Aguayo J, Cerf I, Folscher AB, Fourrier-Jeandel C, Ioos R, Matthews MC, et al. First report of *Fusarium oxysporum* f. sp. *cubense* tropical race 4 (TR4) causing banana wilt in the Island of Mayotte. Plant Dis. 2021;105(1):219. Epub 2020/08/07. doi: 10.1094/PDIS-06-20-1196-PDN. PubMed PMID: 32755365.

54. Mmadi M, Azali HA, Mostert D, Robène I, Viljoen A. First Report of Fusarium Wilt of Cavendish Bananas Caused by *Fusarium oxysporum* f. sp. *cubense* Tropical Race 4 in the Grande Comoros Island. Plant Disease. 2023;107(12):4029. doi: 10.1094/pdis-07-23-1288-pdn. PubMed PMID: 37526488.

55. Beran P, Stehlikova D, Cohen SP, Rost M, Beranova K, Curn V. Utilization of a New Hundred-Genomes Pipeline to Design a Rapid Duplex LAMP Detection Assay for Xanthomonas euvesicatoria and X. vesicatoria in Tomato. Plant Dis. 2023;107(6):1822-8. Epub 20230601. doi: 10.1094/PDIS-05-22-1098-RE. PubMed PMID: 36428255.

56. Wang Z, Guo L, Tan X, Deng J, Gong S, Li D, et al. Development of Loop-Mediated Isothermal Amplification Assays for the Rapid and Accurate Diagnosis of *Exserohilum turcicum* for Field Applications. Plant Disease. 2024;108(6):1461-9. doi: 10.1094/pdis-10-23-2101-sr. PubMed PMID: 38240714.

57. Carvalhais LC, Henderson J, Rincon-Florez VA, O'Dwyer C, Czislowski E, Aitken EAB, et al. Molecular Diagnostics of Banana Fusarium Wilt Targeting Secreted-in-Xylem Genes. Front Plant Sci [Internet]. 2019 2019/06/20 PMC6554419]; 10: 547. Available from: https://www.ncbi.nlm.nih.gov/pmc/articles/PMC6554419/pdf/fpls-10-00547.pdf.

58. Lischer HEL, Shimizu KK. Reference-guided *de novo* assembly approach improves genome reconstruction for related species. BMC Bioinformatics [Internet]. 2017 Nov 10; 18(1): 474. Available from: https://link.springer.com/content/pdf/10.1186/s12859-017-1911-6.pdf.

59. Martineau RL, Murray SA, Ci S, Gao W, Chao S-h, Meldrum DR. Improved Performance of Loop-Mediated Isothermal Amplification Assays via Swarm Priming. Analytical Chemistry. 2017;89(1):625-32. doi: 10.1021/acs.analchem.6b02578.

60. Fraser-Smith S, Czislowski E, Meldrum RA, Zander M, O'Neill W, Balali GR, et al. Sequence variation in the putative effector gene SIX8 facilitates molecular differentiation of *Fusarium oxysporum* f. sp *cubense*. Plant Pathology. 2014;63(5):1044-52. doi: 10.1111/ppa.12184. PubMed PMID: WOS:000342808300009.

61. Lievens B, Rep M, Thomma BP. Recent developments in the molecular discrimination of *formae speciales* of *Fusarium oxysporum*. Pest Manag Sci. 2008;64(8):781-8. doi: 10.1002/ps.1564. PubMed PMID: 18335459.

62. Zhu B. Degradation of plasmid and plant DNA in water microcosms monitored by natural transformation and real-time polymerase chain reaction (PCR). Water Res. 2006;40(17):3231-8. Epub 20060901. doi: 10.1016/j.watres.2006.06.040. PubMed PMID: 16945402.

63. Si NW, Li J, Gao HF, Li YJ, Zhai SS, Xiao F, et al. Effect of the Matrix and Target on the Accurate Quantification of Genomic and Plasmid DNA by Digital Polymerase Chain Reaction.

Agriculture-Basel [Internet]. 2023 Jan; 13(1): 127. Available from: https://doi.org/10.3390/agriculture13010127.

Formatted: Indent: Left: 0", Hanging: 0.39"

Supporting information

S1 Methods. DNA extraction protocol.

S1 Fig: Plasmid diagram

S2 Fig: Primer ~~optimization~~optimisation recipe comparison test

S3 Fig: Sensitivity results on conidia dilutions-spiked plant tissue

S1 Table: Detailed information of the genomic data used in this study.

S2 Table: Detailed parameters of the KEC candidates and their derived primers.

S3 Table: Detailed information of the DNA isolates used in this study.

S4 Table: S4_Table. Detailed informations on the LAMP candidates as compared with LAMP and PCR/qPCR assays already published.

S5 Table: Detailed Specificity Results of our LAMP candidates compared with qPCRs and LAMP references.

S6 Table: Detailed Sensitivity Results of our LAMP candidates compared with qPCRs and LAMP references.

S7 Table: Detailed TR4 Diagnostic results of test done on plants of our LAMP candidates compared with qPCR and LAMP references.

Formatted: Font: Bold
Field Code Changed
Formatted: Font: Bold
Field Code Changed
Formatted: Font: Bold
Formatted: Font: Bold
Formatted: Font: Bold
Field Code Changed
Field Code Changed
Formatted: Font: Bold
Field Code Changed
Formatted: Font: Bold
Field Code Changed
Formatted: Font: Bold
Formatted: Font: Bold
Field Code Changed
Field Code Changed
Formatted: Font: Bold
Field Code Changed
Formatted: Font: Bold

Response to reviewers [PONE-D-25-47570]

1. Journal requirements

1. Please ensure that your manuscript meets PLOS ONE's style requirements, including those for file naming. The PLOS ONE style templates can be found at
https://journals.plos.org/plosone/s/file?id=wjVg/PLOSOne_formatting_sample_main_body.pdf and
https://journals.plos.org/plosone/s/file?id=ba62/PLOSOne_formatting_sample_title_authors_affiliations.pdf

Response :

Thank you for these precise guidelines. We carefully checked them, and edited the text accordingly. As an example, Table and Figure references have been reformatted. Similarly, the authors affiliations have been edited accordingly. Meanwhile, we kept the ORCID affiliations on the first page, as the Guidelines did not require to erase them.

2. Thank you for stating the following in the Acknowledgments Section of your manuscript:
"This study was funded by the Projet France-Relance (PFR) CIRAD-QUALIPLANTE (2022-2024), and by the European Union Horizon 2020 Research and Innovation Program Marie Sklodowska Curie Fellowship, through the INDICANTS project grant nr 890856."
We note that you have provided funding information that is not currently declared in your Funding Statement. However, funding information should not appear in the Acknowledgments section or other areas of your manuscript. We will only publish funding information present in the Funding Statement section of the online submission form.
Please remove any funding-related text from the manuscript and let us know how you would like to update your Funding Statement. Currently, your Funding Statement reads as follows: …

Response :

The Acknowledgements have been modified, and the Financial Disclosure Statement has been precised following your comments.

3. We note that the grant information you provided in the 'Funding Information' and 'Financial Disclosure' sections do not match.

When you resubmit, please ensure that you provide the correct grant numbers for the awards you received for your study in the 'Funding Information' section.

Response :

As precised above, this section has been checked, and edited for better precision.

4. Thank you for stating the following financial disclosure: "This study was funded by the Projet

France-Relance (PFR) CIRAD-QUALIPLANTE (2022-2024), and by the European Union Horizon 2020 Research and Innovation Program Marie Sklodowska-Curie Fellowship, through the INDICANTS project grant nr 890856.”
Please state what role the funders took in the study. If the funders had no role, please state: "The funders had no role in study design, data collection and analysis, decision to publish, or preparation of the manuscript."
If this statement is not correct you must amend it as needed.
Please include this amended Role of Funder statement in your cover letter; we will change the online submission form on your behalf.

Response :

Thank you for this comment. As precised above, this section has been edited for better precision. As you mentioned, funders had no role in the study design and the scientific strategy.

5. Thank you for stating the following financial disclosure:

“This study was funded by the Projet France-Relance (PFR) CIRAD-QUALIPLANTE (2022-2024), and by the European Union Horizon 2020 Research and Innovation Program Marie Sklodowska-Curie Fellowship, through the INDICANTS project grant nr 890856.”

We note that one or more of the authors is affiliated with the funding organization, indicating the funder may have had some role in the design, data collection, analysis or preparation of your manuscript for publication; in other words, the funder played an indirect role through the participation of the co-authors. If the funding organization did not play a role in the study design, data collection and analysis, decision to publish, or preparation of the manuscript and only provided financial support in the form of authors' salaries and/or research materials, please do the following:
1. Review your statements relating to the author contributions, and ensure you have specifically and accurately indicated the role(s) that these authors had in your study. These amendments should be made in the online form.

2. Confirm in your cover letter that you agree with the following statement, and we will change the online submission form on your behalf:

“The funder provided support in the form of salaries for authors [insert relevant initials], but did not have any additional role in the study design, data collection and analysis, decision to publish, or preparation of the manuscript. The specific roles of these authors are articulated in the ‘author contributions’ section.

Response :

Following your comments, the financial disclosure statement has been completed, expliciting the support to the authors. **The new paragraph, to be included in the cover letter and the revision, is :**

This study was funded by the ANR-funded Projet France-Relance (PFR) CIRAD-QUALIPLANTE (2022-2024), the European Union Horizon 2020 Research and Innovation Program Marie Sklodowska-Curie Fellowship, through the INDICANTS project grant nr 890856, and co-funded by the European Union, the French Government and the « Région Réunion : L' Europe s'engage à La Réunion avec le FEADER », project grant REU77071-1-000011.

PFR provided the salary of MA, INDICANTS provided the salary of IR and contributed to the salary of MA, while FEADER provided the salaries of VML and BF.

The funders had no role in study design, data collection and analysis, decision to publish, or preparation of the manuscript.

6. We note that you have included the phrase "data not shown" in your manuscript. Unfortunately, this does not meet our data sharing requirements. PLOS does not permit references to inaccessible data. We require that authors provide all relevant data within the paper, Supporting Information files, or in an acceptable, public repository. Please add a citation to support this phrase or upload the data that corresponds with these findings to a stable repository (such as Figshare or Dryad) and provide and URLs, DOIs, or accession numbers that may be used to access these data. **Or, if the data are not a core part of the research being presented in your study, we ask that you remove the phrase that refers to these data.**

Response :

Thank you for this remark. We modified the text accordingly at the four occurrences. The phrase has been removed in two cases, while the sentence has been reworded in the two other cases.

7. Please include your full ethics statement in the 'Methods' section of your manuscript file. In your statement, please include the full name of the IRB or ethics committee who approved or waived your study, as well as whether or not you obtained informed written or verbal consent. If consent was waived for your study, please include this information in your statement as well.

Response :

The Methods section has been completed according to your comment, with the following sentence :
« Field samplings were done in Java, Indonesia, as granted by the National Research and Innovation Agency BRIN (Badan Riset dan Inovasi Nasional, https://www.brin.go.id). »

8. Please ensure that you refer to Figure 3 in your text as, if accepted, production will need this reference to link the reader to the figure.

Response :

The Figure 3 is mentionned in the main text, line 450 (Page 22) of the revised version.

9. We note you have included a table to which you do not refer in the text of your

manuscript. Please ensure that you refer to Table 4 in your text; if accepted, production will need this reference to link the reader to the Table.

Response :

Thank you for this comment. The Table 4 is cited in the main text, line 462 (p. 23) of the revised version.

10. Thank you for providing your underlying data as Supporting Information.

We note that the data set contains text or data that is not in English. Please note that PLOS is an English-language publisher, so we require data sets to be provided in English as well. Please upload an English-language version of your data set.
This will also allow us to determine if your data follows PLOS standards per our Data Availability policy here: https://journals.plos.org/plosone/s/data-availability

Response :

We thank you for this comment and apologize for these errors. All Supplementary Figures and Tables have been checked, and edited accordingly, notably on some Country names and the sample references in S7 Table.

11. If the reviewer comments include a recommendation to cite specific previously published works, please review and evaluate these publications to determine whether they are relevant and should be cited. There is no requirement to cite these works unless the editor has indicated otherwise.

Response :

The relevant literature has been reviewed and discussed both in introduction and discussion. The reviewers did not request any addition of publications.

2. Reviewer #1:

- **Table 1 and Table 4: Error in writing (f.sp.) should be corrected as f.sp.**

Response :

These tables were corrected following your comment, for Foc.

- **In Table 1 and Table 4 under race column many rows are blank which need to be filled. If anyone is not known is to be given as unknown**

Response :

These tables were corrected following your comment, for Foc.

- **Line 279: Define 10^0 clearly. is it possible to prepare serial dilution till to get nil conidia? Or it is mere control.**

Response :

Thank you for this comment. 10^0 corresponds to 1 conidia per mL, which can be reachable using serial dilution.

- **In the section "In-field deployment and detection from naturally infested plant material" (line 303), further details are required. Specifically, was internal severity of Foc assessed for all the samples? Additionally, please indicate whether the varieties grown were Cavendish or non-Cavendish, and specifically their genome.**

Response :

The plant samples considered carried clear Fusarium wilt symptoms. Internal symptoms severity was assessed for all samples by visual inspection of vascular discoloration after transverse sectioning of the pseudostem or by opening a small window in the pseudostem to avoid destroying the plant.

As detailed in the S7 Table, cultivars included both Cavendish and non-Cavendish (Pisang Kepok, Pisang Barangan, etc…), of various genomic formulas (AAA, AAB, ABB).

- **Is this LAMP assay able to give inoculum load in soil? If yes, whether it was validated with spore count?**

Response :

This LAMP assay was primarily designed for point-of-care diagnostic on plants, and has not been tested yet on soil samples.

- **All the supplementary files could not be accessed which need to be given in easy accessible form.**

Response :

We are sorry for that ; Supplemental Figures and Tables were yet all provided to the publisher website.

- **Legends for tables and figures may be arranged at the end of the manuscript.**

Response :

Thank you for this comment. Legends for tables and Figures have been edited following Plos One guidelines.

3. Reviewer #2:

- **The manuscript should emphasize how the KEC-based marker identification improves upon previous marker discovery methods and, where possible, quantify the comparative improvement in specificity and sensitivity.**

Response:

Thank you for this comment. Several marker discovery strategies were evaluated prior to selecting the K-mer Elimination by Cross-reference (KEC) approach. First, an ortho-group filtering and mapping strategy targeting putative TR4-specific genes resulted in only a single candidate gene, and the corresponding LAMP primers lacked sufficient specificity. Second, an Illumina read mapping and filtering approach against a Foc-TR4 reference genome was tested; however, this method was computationally intensive and not fully inclusive, likely due to incomplete coverage and assembly gaps in some genomes.

In contrast, the KEC strategy proved to be the most powerful and flexible approach, as it allowed the identification of multiple candidate genomic regions without relying on gene annotation or predefined loci. This enabled subsequent validation steps to assess both inclusivity and specificity across a large and diverse genome panel. Compared with other previously used approaches such as DArTseq-based marker discovery or polymorphism detection in known effector genes (e.g. SIX genes), which are inherently biased toward reduced genome representations or specific gene families, KEC enabled unbiased, genome-wide identification of candidate regions of sufficient length for LAMP primer design.

Although the initial KEC output included some non-specific candidates, subsequent BLAST filtering and Illumina read mapping allowed us to select a single marker (Candidate 23) that was present in all 120 Foc-TR4 genomes tested and absent from all non-target genomes, including all other known Foc VCGs and related Fusarium species. This resulted in a marked improvement in both specificity and inclusivity compared to alternative strategies, as demonstrated experimentally using an extensive specificity panel. These comparative advantages are now more clearly emphasized in the revised Discussion section.

- **Table 1 is excessively detailed for the main text; consider moving the comprehensive genome list to the Supplementary Material.**

Response :

Following your comment, the Table has been shortened and lightened.

- **Remove the period (".") preceding units (e.g., μL^{-1}, mL) throughout the text.**

Response :

The text has been checked and edited following your comment.

- **Line 34: The sentence appears incomplete; please revise for clarity.**

Response :

Following your comment, the sentence has been changed to : « The high genetic diversity present among Foc isolates further complicates the identification of unique genomic regions suitable for diagnosis. »

- **Line 54: Replace “The causing agent is Fusarium oxysporum f. sp. cubense” with “The causal agent is Fusarium oxysporum f. sp. cubense.”**

Response :

The text has been edited following your comment.

- **Line 59: Replace “export” with “exports.”**

Response :

The text has been edited following your comment.

- **Lines 79–81: Rephrase for clarity and readability.**

Response :

The sentence about the use of core-genes has been rephrased following your comment.

- **Line 249: Replace “grinded” with “ground.”**

Response :

The text has been edited following your comment.

- **Line 272: Replace "eluded" with "eluted."**

Response :

The text has been edited following your comment.

- **Line 298: Replace "sensibility" with "sensitivity."**

Response :

The text has been edited following your comment.

- **Line 359: The statement is unclear; please revise to improve clarity.**

Response :

The sentence has been rephrased following your comment. We hope that it is clearer.

- **Ensure consistent use of µL^{-1} (superscript minus) or "per µL" throughout the text.**

Response :

The text has been edited following your comment.

- **Standardize spelling conventions (e.g., visualized vs visualised, optimized vs optimised) and apply consistently.**

Response :

We chose the British english spelling. The text has been checked and edited following your comment.

- **Italicize all scientific names (e.g., F. oxysporum, F. cubense).**

Response :

The text, tables and figures have been edited following your comment.

- **Use en-dashes consistently in numerical ranges (e.g., “30–40°C” instead of “30-40°C”).**

Response :

The text has been checked and edited following your comment.

- **A thorough proofreading is recommended to correct typographical and grammatical errors throughout the manuscript.**

Response :

The text has been carefully checked and edited when necessary, following your comment.

We thank the two reviewers for their careful review and comments.
