# Supplementary material for: Comparative genomics-based development of a LAMP assay for rapid and reliable in-field detection of Fusarium oxysporum f. sp. cubense tropical race 4
Source: PLoS One. 2026 Apr 29;21(4):e0347645. doi: 10.1371/journal.pone.0347645 (PMC13127957; doi:10.1371/journal.pone.0347645)
Supplement: Attachment — Submitted filename: PONE-D-25-47570_Response to reviewers_VF.docx [file pone.0347645.s015.docx]

**Response to reviewers [PONE-D-25-47570]**

1. **Journal requirements**

**1. Please ensure that your manuscript meets PLOS ONE's style requirements, including those for file naming. The PLOS ONE style templates can be found at** 
<https://journals.plos.org/plosone/s/file?id=wjVg/PLOSOne_formatting_sample_main_body.pdf> and 
<https://journals.plos.org/plosone/s/file?id=ba62/PLOSOne_formatting_sample_title_authors_affiliations.pdf>

**Response :**

Thank you for these precise guidelines. We carefully checked them, and edited the text accordingly. As an example, Table and Figure references have been reformatted. Similarly, the authors affiliations have been edited accordingly. Meanwhile, we kept the ORCID affiliations on the first page, as the Guidelines did not require to erase them.

**2. Thank you for stating the following in the Acknowledgments Section of your manuscript:** 
“This study was funded by the Projet France-Relance (PFR) CIRAD-QUALIPLANTE (2022-2024), and by the European Union Horizon 2020 Research and Innovation Program Marie Sklodowska Curie Fellowship, through the INDICANTS project grant nr 890856.”
We note that you have provided funding information that is not currently declared in your Funding Statement. However, funding information should not appear in the Acknowledgments section or other areas of your manuscript. We will only publish funding information present in the Funding Statement section of the online submission form. 
Please remove any funding-related text from the manuscript and let us know how you would like to update your Funding Statement. Currently, your Funding Statement reads as follows: …

**Response :**

The Acknowledgements have been modified, and the Financial Disclosure Statement has been precised following your comments.

**3. We note that the grant information you provided in the ‘Funding Information’ and ‘Financial Disclosure’ sections do not match.**

When you resubmit, please ensure that you provide the correct grant numbers for the awards you received for your study in the ‘Funding Information’ section.

**Response :**

As precised above, this section has been checked, and edited for better precision.

**4. Thank you for stating the following financial disclosure:** “This study was funded by the Projet France-Relance (PFR) CIRAD-QUALIPLANTE (2022-2024), and by the European Union Horizon 2020 Research and Innovation Program Marie Sklodowska-Curie Fellowship, through the INDICANTS project grant nr 890856.”

Please state what role the funders took in the study.  If the funders had no role, please state: "The funders had no role in study design, data collection and analysis, decision to publish, or preparation of the manuscript."

If this statement is not correct you must amend it as needed.

Please include this amended Role of Funder statement in your cover letter; we will change the online submission form on your behalf.

**Response :**

Thank you for this comment. As precised above, this section has been edited for better precision. As you mentioned, funders had no role in the study design and the scientific strategy.

**5. Thank you for stating the following financial disclosure:**

“This study was funded by the Projet France-Relance (PFR) CIRAD-QUALIPLANTE (2022-2024), and by the European Union Horizon 2020 Research and Innovation Program Marie Sklodowska-Curie Fellowship, through the INDICANTS project grant nr 890856.”

We note that one or more of the authors is affiliated with the funding organization, indicating the funder may have had some role in the design, data collection, analysis or preparation of your manuscript for publication; in other words, the funder played an indirect role through the participation of the co-authors. If the funding organization did not play a role in the study design, data collection and analysis, decision to publish, or preparation of the manuscript and only provided financial support in the form of authors' salaries and/or research materials, please do the following:

1. Review your statements relating to the author contributions, and ensure you have specifically and accurately indicated the role(s) that these authors had in your study. These amendments should be made in the online form.

2. Confirm in your cover letter that you agree with the following statement, and we will change the online submission form on your behalf:

“The funder provided support in the form of salaries for authors [insert relevant initials], but did not have any additional role in the study design, data collection and analysis, decision to publish, or preparation of the manuscript. The specific roles of these authors are articulated in the ‘author contributions’ section.

**Response :**

Following your comments, the financial disclosure statement has been completed, expliciting the support to the authors. **The new paragraph, to be included in the cover letter and the revision, is :**

**This study was funded by the ANR-funded Projet France-Relance (PFR) CIRAD-QUALIPLANTE (2022-2024), the European Union Horizon 2020 Research and Innovation Program Marie Sklodowska-Curie Fellowship, through the INDICANTS project grant nr 890856, and co-funded by the European Union, the French Government and the « Région Réunion : L' Europe s'engage à La Réunion avec le FEADER », project grant REU77071-1-000011.**

**PFR provided the salary of MA, INDICANTS provided the salary of IR and contributed to the salary of MA, while FEADER provided the salaries of VML and BF.**

**The funders had no role in study design, data collection and analysis, decision to publish, or preparation of the manuscript.**

**6. We note that you have included the phrase “data not shown” in your manuscript. Unfortunately, this does not meet our data sharing requirements.** PLOS does not permit references to inaccessible data. We require that authors provide all relevant data within the paper, Supporting Information files, or in an acceptable, public repository. Please add a citation to support this phrase or upload the data that corresponds with these findings to a stable repository (such as Figshare or Dryad) and provide and URLs, DOIs, or accession numbers that may be used to access these data. **Or, if the data are not a core part of the research being presented in your study, we ask that you remove the phrase that refers to these data.**

**Response :**

Thank you for this remark. We modified the text accordingly at the four occurrences. The phrase has been removed in two cases, while the sentence has been reworded in the two other cases.

**7. Please include your full ethics statement in the ‘Methods’ section of your manuscript file. In your statement, please include the full name of the IRB or ethics committee who approved or waived your study, as well as whether or not you obtained informed written or verbal consent. If consent was waived for your study, please include this information in your statement as well.**

**Response :**

The Methods section has been completed according to your comment, with the following sentence :

« Field samplings were done in Java, Indonesia, as granted by the National Research

and Innovation Agency BRIN (Badan Riset dan Inovasi Nasional, <https://www.brin.go.id>). »

**8. Please ensure that you refer to Figure 3 in your text as, if accepted, production will need this reference to link the reader to the figure.**

**Response :**

The Figure 3 is mentionned in the main text, line 450 (Page 22) of the revised version.

**9. We note you have included a table to which you do not refer in the text of your manuscript. Please ensure that you refer to Table 4 in your text; if accepted, production will need this reference to link the reader to the Table.**

**Response :**

Thank you for this comment. The Table 4 is cited in the main text, line 462 (p. 23) of the revised version.

**10. Thank you for providing your underlying data as Supporting Information.**

We note that the data set contains text or data that is not in English. Please note that PLOS is an English-language publisher, so we require data sets to be provided in English as well. Please upload an English-language version of your data set.

This will also allow us to determine if your data follows PLOS standards per our Data Availability policy here: https://journals.plos.org/plosone/s/data-availability

**Response :**

We thank you for this comment and apologize for these errors. All Supplementary Figures and Tables have been checked, and edited accordingly, notably on some Country names and the sample references in S7 Table.

**11. If the reviewer comments include a recommendation to cite specific previously published works, please review and evaluate these publications to determine whether they are relevant and should be cited. There is no requirement to cite these works unless the editor has indicated otherwise.**

**Response :**

The relevant literature has been reviewed and discussed both in introduction and discussion. The reviewers did not request any addition of publications.

2. **Reviewer #1:**

- **Table 1 and Table 4: Error in writing (f.sp.) should be corrected as f.sp.**

**Response :**

These tables were corrected following your comment, for Foc.

- **In Table 1 and Table 4 under race column many rows are blank which need to be filled. If anyone is not known is to be given as unknown**

**Response :**

These tables were corrected following your comment, for Foc.

- **Line 279: Define 10^0 clearly. is it possible to prepare serial dilution till to get nil conidia? Or it is mere control.**

**Response :**

Thank you for this comment. 10^0^ corresponds to 1 conidia per mL, which can be reachable using serial dilution.

- **In the section “In-field deployment and detection from naturally infested plant material” (line 303), further details are required. Specifically, was internal severity of Foc assessed for all the samples? Additionally, please indicate whether the varieties grown were Cavendish or non-Cavendish, and specifically their genome.**

**Response :**

The plant samples considered carried clear Fusarium wilt symptoms. Internal symptoms severity was assessed for all samples by visual inspection of vascular discoloration after transverse sectioning of the pseudostem or by opening a small window in the pseudostem to avoid destroying the plant.

As detailed in the S7 Table, cultivars included both Cavendish and non-Cavendish (Pisang Kepok, Pisang Barangan, etc…), of various genomic formulas (AAA, AAB, ABB).

- **Is this LAMP assay able to give inoculum load in soil? If yes, whether it was validated with spore count?**

**Response :**

This LAMP assay was primarily designed for point-of-care diagnostic on plants, and has not been tested yet on soil samples.

- **All the supplementary files could not be accessed which need to be given in easy accessible form.**

**Response :**

We are sorry for that ; Supplemental Figures and Tables were yet all provided to the publisher website.

- **Legends for tables and figures may be arranged at the end of the manuscript.**

**Response :**

Thank you for this comment. Legends for tables and Figures have been edited following Plos One guidelines.

3. **Reviewer #2:**

- **The manuscript should emphasize how the KEC-based marker identification improves upon previous marker discovery methods and, where possible, quantify the comparative improvement in specificity and sensitivity.**

**Response:**

Thank you for this comment. Several marker discovery strategies were evaluated prior to selecting the K-mer Elimination by Cross-reference (KEC) approach. First, an ortho-group filtering and mapping strategy targeting putative TR4-specific genes resulted in only a single candidate gene, and the corresponding LAMP primers lacked sufficient specificity. Second, an Illumina read mapping and filtering approach against a Foc-TR4 reference genome was tested; however, this method was computationally intensive and not fully inclusive, likely due to incomplete coverage and assembly gaps in some genomes.

In contrast, the KEC strategy proved to be the most powerful and flexible approach, as it allowed the identification of multiple candidate genomic regions without relying on gene annotation or predefined loci. This enabled subsequent validation steps to assess both inclusivity and specificity across a large and diverse genome panel. Compared with other previously used approaches such as DArTseq-based marker discovery or polymorphism detection in known effector genes (e.g. SIX genes), which are inherently biased toward reduced genome representations or specific gene families, KEC enabled unbiased, genome-wide identification of candidate regions of sufficient length for LAMP primer design.

Although the initial KEC output included some non-specific candidates, subsequent BLAST filtering and Illumina read mapping allowed us to select a single marker (Candidate 23) that was present in all 120 Foc-TR4 genomes tested and absent from all non-target genomes, including all other known Foc VCGs and related Fusarium species. This resulted in a marked improvement in both specificity and inclusivity compared to alternative strategies, as demonstrated experimentally using an extensive specificity panel. These comparative advantages are now more clearly emphasized in the revised Discussion section.

- **Table 1 is excessively detailed for the main text; consider moving the comprehensive genome list to the Supplementary Material.**

**Response :**

Following your comment, the Table has been shortened and lightened.

- **Remove the period (".") preceding units (e.g., µL⁻¹, mL) throughout the text.**

**Response :**

The text has been checked and edited following your comment.

- **Line 34: The sentence appears incomplete; please revise for clarity.**

**Response :**

Following your comment, the sentence has been changed to : « The high genetic diversity present among Foc isolates further complicates the identification of unique genomic regions suitable for diagnosis. »

- **Line 54: Replace “The causing agent is Fusarium oxysporum f. sp. cubense” with “The causal agent is Fusarium oxysporum f. sp. cubense.”**

**Response :**

The text has been edited following your comment.

- **Line 59: Replace “export” with “exports.”**

**Response :**

The text has been edited following your comment.

- **Lines 79–81: Rephrase for clarity and readability.**

**Response :**

The sentence about the use of core-genes has been rephrased following your comment.

- **Line 249: Replace “grinded” with “ground.”**

**Response :**

The text has been edited following your comment.

- **Line 272: Replace “eluded” with “eluted.”**

**Response :**

The text has been edited following your comment.

- **Line 298: Replace “sensibility” with “sensitivity.”**

**Response :**

The text has been edited following your comment.

- **Line 359: The statement is unclear; please revise to improve clarity.**

**Response :**

The sentence has been rephrased following your comment. We hope that it is clearer.

- **Ensure consistent use of µL⁻¹ (superscript minus) or “per µL” throughout the text.**

**Response :**

The text has been edited following your comment.

- **Standardize spelling conventions (e.g., visualized vs visualised, optimized vs optimised) and apply consistently.**

**Response :**

We chose the British english spelling. The text has been checked and edited following your comment.

- **Italicize all scientific names (e.g., F. oxysporum, F. cubense).**

**Response :**

The text, tables and figures have been edited following your comment.

- **Use en-dashes consistently in numerical ranges (e.g., “30–40°C” instead of “30-40°C”).**

**Response :**

The text has been checked and edited following your comment.

- **A thorough proofreading is recommended to correct typographical and grammatical errors throughout the manuscript.**

**Response :**

The text has been carefully checked and edited when necessary, following your comment.

We thank the two reviewers for their careful review and comments.
