# Supplementary material for: Comparative genomics-based development of a LAMP assay for rapid and reliable in-field detection of Fusarium oxysporum f. sp. cubense tropical race 4
Source: PLoS One. 2026 Apr 29;21(4):e0347645. doi: 10.1371/journal.pone.0347645 (PMC13127957; doi:10.1371/journal.pone.0347645)
Supplement: Attachment — Submitted filename: PONE-D-25-47570R1_Response to reviewers.docx [file pone.0347645.s016.docx]

**[PONE-D-25-47570R1] - [EMID:6c2c8749d70cf63e] : Response to reviewers**

**Reviewer #1: MS "Comparative genomics-based development of a LAMP assay for rapid and reliable in-field detection of Fusarium oxysporum f. sp. cubense tropical race 4" has been improvised. However the minor annotations (italics, typo errors etc) as noted in the MS need to be taken care.**

Thank you for acknowledging the improvement of the MN.

Following these general and not specific comments, we carefully checked, in a second round, the main text, Tables and Figures, and Supplemental Material.

Doing this we identified some typo errors, that we edited following the Authors guidelines. We also changed some formulations to gain in clarity (see for example lines 334-335, 340, 398,426, etc..). The literature section was also carefully checked.
